# *Dmc1* is a candidate for temperature tolerance during wheat meiosis

**DOI:** 10.1007/s00122-019-03508-9

**Published:** 2019-12-18

**Authors:** Tracie Draeger, Azahara C. Martin, Abdul Kader Alabdullah, Ali Pendle, María-Dolores Rey, Peter Shaw, Graham Moore

**Affiliations:** 1 John Innes Centre, Norwich Research Park, Norwich, NR4 7UH UK; 2 Agroforestry and Plant Biochemistry, Proteomics and Systems Biology, Department of Biochemistry and Molecular Biology, University of Cordoba, Cordoba, Spain

## Abstract

**Key message:**

The meiotic recombination gene *Dmc1* on wheat chromosome 5D has been identified as a candidate for the maintenance of normal chromosome synapsis and crossover at low and possibly high temperatures.

**Abstract:**

We initially assessed the effects of low temperature on meiotic chromosome synapsis and crossover formation in the hexaploid wheat (*Triticum aestivum* L.) variety ‘Chinese Spring’. At low temperatures, asynapsis and chromosome univalence have been observed before in Chinese Spring lines lacking the long arm of chromosome 5D (5DL), which led to the proposal that 5DL carries a gene (*Ltp1*) that stabilises wheat chromosome pairing at low temperatures. In the current study, Chinese Spring wild type and 5DL interstitial deletion mutant plants were exposed to low temperature in a controlled environment room during a period from premeiotic interphase to early meiosis I. A 5DL deletion mutant was identified whose meiotic chromosomes exhibit extremely high levels of asynapsis and chromosome univalence at metaphase I after 7 days at 13 °C, suggesting that *Ltp1* is deleted in this mutant. Immunolocalisation of the meiotic proteins ASY1 and ZYP1 on *ltp1* mutants showed that low temperature results in a failure to complete synapsis at pachytene. KASP genotyping revealed that the *ltp1* mutant has a 4-Mb deletion in 5DL. Of 41 genes within this deletion region, the strongest candidate for the stabilisation of chromosome pairing at low temperatures is the meiotic recombination gene *Dmc1.* The *ltp1* mutants were subsequently treated at 30 °C for 24 h during meiosis and exhibited a reduced number of crossovers and increased univalence, though to a lesser extent than at 13 °C. We therefore renamed our *ltp1* mutant ‘*ttmei1*’ (*t*emperature-*t*olerant *mei*osis *1*) to reflect this additional loss of high temperature tolerance.

**Electronic supplementary material:**

The online version of this article (10.1007/s00122-019-03508-9) contains supplementary material, which is available to authorized users.

## Introduction

In plants, male reproductive development is highly sensitive to adverse environmental conditions including high and low temperatures (De Storme and Geelen 2014). In wheat, the reproductive phase is more sensitive to high temperatures than the vegetative phase (Fischer and Maurer 1976). This is also the case for low temperatures, with meiosis I identified as the most sensitive stage (Thakur et al. 2010). High temperatures during reproductive development can have a negative impact on grain yield (Fischer and Maurer 1976; Fischer 1985; Wardlaw et al. 1989), and even short periods of a moderately high temperature (20–24 h at 30 °C) during meiosis can reduce grain number (Saini and Aspinall 1982; Draeger and Moore 2017). Grain yield is also reduced when low temperatures occur during the booting stage (Ji et al. 2017), which broadly corresponds to meiosis (Barber et al. 2015).

In cereal crops such as wheat, where grains are an important yield factor, stress-induced male sterility generally has a negative effect on crop yield and performance (De Storme and Geelen 2014). In the UK and France in the mid-1980s, cold and wet weather during floral development in the wheat variety ‘Moulin’ caused significant sterility, resulting in a reduction in grain yield of more than 70% (Law 1999). The dramatic falls in yield were thought to be a result of reduced fertility caused by the occurrence of low temperatures at meiosis. It was suggested that Moulin might carry specific alleles from diverse sources that made the wheat more sensitive to cold weather during meiosis.

Meiosis is essential for gamete formation in sexually reproducing organisms. In early meiosis, homologous chromosomes align next to each other as pairs, and then, during synapsis, the pairs of homologs become linked tightly with each other through the polymerisation of a protein structure called the synaptonemal complex (SC), which assembles between the paired chromosomes (Page and Hawley 2004). The SC has a ladder-like structure consisting of two chromosome axes and a central region. Its assembly can be tracked by immunolocalisation of the meiotic proteins ASY1, which interacts with the chromosome axes (Boden et al. 2009), and ZYP1, which is associated with the central region (Higgins et al. 2005; Khoo et al. 2012). During zygotene, the axes of the two homologs begin to become connected by the assembly of the central region between them. The axes are now called lateral elements. At pachytene, the central structure links the homologs along their entire lengths and synapsis is completed. SC assembly is a highly temperature-sensitive process (Bilgir et al. 2013).

The SC is thought to provide the structural framework for meiotic recombination to take place. During recombination, at least one crossover (CO) must form between each pair of homologs, to forge a physical connection between the chromosomes. These physical connections can be seen cytologically and are called chiasmata. COs enable genetic information to be exchanged between chromosomes and are also needed for accurate chromosome segregation and balanced gametes in the daughter cells. Once COs have fully formed (at pachytene), the SC is disassembled (at diplotene), at which point the homologs are only connected via their chiasmata. They remain connected until the chromosomes segregate at anaphase I.

In bread wheat, which is an allopolyploid with three homeologous (related) diploid genomes (AABBDD), COs only form between homologs and not between homeologs, ensuring that the three genomes behave as diploids during meiosis. Normally, at metaphase I, only ring bivalents (with two chiasmata) and occasional rod bivalents (one chiasma) are present. At metaphase I, the 21 bivalents align on the equatorial plate before segregating at anaphase I.

Correct pairing and segregation of homologs is vital for maintaining the stability and fertility of the genome. Errors in meiosis can lead to aneuploidy or infertility. High and low temperatures can induce a variety of meiotic aberrations in plants including changes in the frequency of chiasma formation (CO frequency) (Elliott 1955; Dowrick 1957; Bayliss and Riley 1972a; Higgins et al. 2012). Reduction in chiasma formation at extremes of temperature is linked to disruption of chromosome synapsis. This can result in unpaired univalent chromosomes that segregate randomly during meiosis I or are lost completely (Bomblies et al. 2015). Temperature-associated synapsis failure has been reported in plants at both low and high temperatures, but the temperature at which meiosis fails varies in different species (reviewed in Bomblies et al. 2015). These temperature thresholds can also vary within a species where there are genotypic differences (Riley et al. 1966).

Chinese Spring is one of the more heat-sensitive wheat cultivars (Qin et al. 2008) and has been widely used to study the frequency of chiasma formation at low and high temperatures because different Chinese Spring genotypes respond differently to temperature extremes. At low temperatures, a reduction in chiasma frequency and an increase in chromosome univalence have been observed in Chinese Spring nullisomic 5D-tetrasomic 5B (N5DT5B) plants, which lack chromosome 5D and have two extra copies of 5B (Riley 1966; Bayliss and Riley 1972a). Wheat has an optimum temperature range of around 17–23 °C over the course of a growing season (Porter and Gawith 1999). In N5DT5B plants, the frequency of chiasma formation is progressively reduced as the temperature rises above or falls below the optimum range (Bayliss and Riley 1972a). At 15 °C, chiasma frequency is greatly reduced in N5DT5B plants (Riley 1966), and they exhibit pronounced chromosome pairing failure at 12 °C, which leads to complete male sterility (Hayter and Riley 1967). The reduced chiasma frequencies seen in N5DT5B plants at low temperatures occur because the chromosomes fail to pair during zygotene, though the temperature-sensitive phase was found to be during premeiotic interphase, prior to DNA synthesis (Bayliss and Riley 1972b).

The asynapsis observed in N5DT5B is also seen in plants that are nullisomic for 5D (40 chromosomes), but in plants that are tetrasomic for 5B (44 chromosomes) synapsis is normal (Riley et al. 1966), so it was inferred that the asynapsis in N5DT5B could not be a result of the extra dosage of 5B, but that there must be a gene on chromosome 5D that stabilises chromosome pairing at low temperatures. This gene was named *low-temperature pairing* (*Ltp*) by Hayter and Riley (1967). *Ltp* was further defined to the long arm of chromosome 5D (Hayter 1969) and later renamed *Ltp1* (Queiroz et al. 1991), but its exact location has never been mapped. It has been suggested that chiasma frequency also falls progressively in N5DT5B plants at temperatures of 30 °C and above (Bayliss and Riley 1972a), indicating that chromosome 5D may also be associated with high temperature tolerance. However, this suggestion was based on the scoring of only a few cells because exposure of the plants to high temperatures for 3 days made the chromosomes too sticky to score accurately. Grain number is also reduced much more in N5DT5B plants than in the wild type after exposure to 30 °C during premeiosis and leptotene (Draeger and Moore 2017).

The main aim of this study was to define *Ltp1* to a small enough region on 5DL to enable the identification of a candidate gene for the low temperature pairing phenotype. The strategy for mapping *Ltp1* was similar to the one used to map the *Ph1* locus (Roberts et al. 1999; Griffiths et al. 2006) a major locus that promotes homolog synapsis and regulates crossover formation in wheat (Martín et al. 2014, 2017). This had involved screening for gamma irradiation-induced deletions on specific chromosomes in large populations of wheat. The work on *Ph1* established the dose of gamma irradiation likely to give a good rate of deletion discovery. This strategy was applied in the current study to identify deletions of chromosome 5DL using chromosome-specific markers. Recent development of resources such as the Chinese Spring IWGSC RefSeq v1.0 genome assembly (International Wheat Genome Sequencing Consortium (IWGSC) 2018), the Wheat 820 K Axiom^®^ Breeders’ Array probe set (Winfield et al. 2016) and the Ensembl Plants database (Bolser et al. 2016), facilitated the processes of mapping and candidate gene identification. Following the identification of a candidate gene for the low-temperature phenotype, additional experiments were carried out to determine whether tolerance to both high and low temperatures could be controlled by the same locus.

## Materials and methods

### Plant materials

Deletion lines were generated in bread wheat (*Triticum aestivum* L., 2*n* = 6*x* = 42) var. ‘Chinese Spring’ by gamma irradiation of 1000 seeds at the International Atomic Energy Agency, Vienna (500 seeds irradiated at 250 Gy and 500 at 300 Gy). M_1_ deletion lines were self-fertilised. M_2_ lines were genotyped using Kompetitive allele-specific PCR (KASP) to identify plants with interstitial deletions of 5DL. These deletion mutants were exposed to 13 °C for 7 days to identify plants with asynaptic chromosomes at metaphase I of meiosis. Two other Chinese Spring-derived genotypes were used as controls for the KASP genotyping and in the temperature treatment experiments, the standard euploid (wild type) form, (AABBDD) and the nullisomic 5D-tetrasomic 5B (N5DT5B) genotype, which lacks chromosome 5D. Chinese Spring lines with homozygous *terminal* deletions of chromosome 5DL were obtained from TR Endo, Kyoto University, Japan. These were 5DL-1, 5DL-2, 5DL-5, 5DL-9 and 5DL-13 (Endo and Gill 1996).

### DNA extractions

Plants were initially grown in modular trays in a controlled environment room (CER) at 20 °C (day) and 15 °C (night) with a 16-h photoperiod (lights on between 10:00 and 02:00) and 70% humidity. Wheat seedlings were grown to the 2–3 leaf stage, and approximately 5 cm of leaf material was harvested into 1.2-ml collection tubes containing 3-mm tungsten-carbide beads in a 96-well format on dry ice. DNA was extracted using a method based on the protocol available at https://www.ars.usda.gov/ARSUserFiles/60701500/SmallGrainsGenotypingLaboratory/DNAextractionprotocol_ERSGGL.pdf (original reference in Pallotta et al. 2003) except that leaf material was ground in a Geno/Grinder (Spex) at 1500 rpm for 2 min. Extracted DNA was diluted with dH_2_0 so that final DNA template concentrations were between 15 and 30 ng.

### Genotyping 5DL terminal deletion lines

**Fig. 1:**
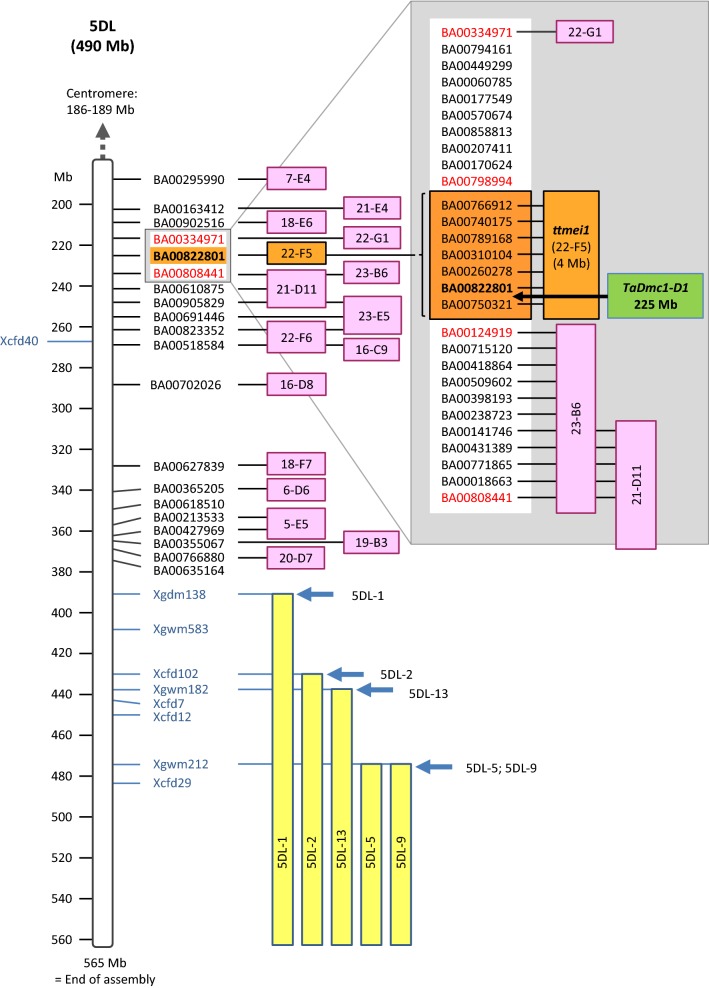
Map of wheat chromosome arm 5DL showing locations of deletions detected using 5DL-specific KASP markers (black or red text with BA prefix) or microsatellite markers (blue); markers are aligned with positions on the Chinese Spring IWGSC RefSeq v1.0 genome assembly (shown in Mb); yellow boxes mark the extent of the 5DL terminal deletions in lines with normal pairing at 13 °C; breakpoints of terminal deletion lines are indicated by blue arrows; pink boxes show positions of 5DL interstitial deletions in lines with normal pairing at 13 °C; orange boxes show the location of the 5DL deletion in the mutant line 22-F5 (*ttmei1*), which exhibits asynapsis at 13 °C; small grey box shows the *ttmei1* deletion region and its flanking markers (in red) as detected in the initial KASP genotyping analysis with the only marker deleted in *ttmei1* (BA00822801) shown in bold text; larger grey inset box shows fine mapping between flanking markers BA00334971 and BA00808441 (red text) using BA00822801 and 25 additional KASP markers. Markers shown in orange boxes are deleted in the *ttmei1* mutant. The position of the candidate gene *TaDmc1*-*D1* (green box) at 225 Mb is indicated with a black arrow

Nine chromosome 5D-specific microsatellite markers (Somers et al. 2004), from the GrainGenes database https://wheat.pw.usda.gov/GG3/, were used to map the breakpoints of the five Chinese Spring 5DL terminal deletion lines (Fig. 1). Following PCR amplification, products were separated by agarose gel electrophoresis.

### KASP genotyping of M_2_ plants

KASP genotyping of M_2_ plants was performed using SNP (single nucleotide polymorphism) markers from the Wheat Breeders’ 820 K Axiom^®^ array (Winfield et al. 2016) available at www.cerealsdb.uk.net. This data set was aligned with the Chinese Spring reference sequence assembly, IWGSC RefSeq v1.0 (IWGSC 2018), using Python Data Matcher https://github.com/wingenl/python_data_matcher, which merges files with common features. The BioMart data mining tool from Ensembl Plants (Kinsella et al. 2011), available at http://plants.ensembl.org/biomart/martview/, was used as a source for the RefSeq v1.0 assembly coordinates.

Initially, KASP markers used for genotyping were selected at 15-Mb intervals along chromosome 5DL, between the centromere and the breakpoint of the most proximal Chinese Spring terminal deletion line. Later, more KASP markers were used to increase the density across regions of interest (Fig. 1). Selected KASP Primers were 5D chromosome specific and had homeologous SNPs at the 3′ end. The allele-specific forward primers and common reverse primers were synthesised by Sigma-Aldrich. Allele-specific primers were synthesised with standard FAM or VIC compatible tails at their 5′ ends (FAM tail: 5′ GAAGGTGACCAAGTTCATGCT 3′; VIC tail: 5′ GAAGGTCGGAGTCAACGGATT 3′). Twenty primer sets were used in the first round of screening but following the identification of the *ttmei1* 5DL deletion mutant, 25 more KASP markers were selected between markers BA00334971 and BA00808441 to fine map the deletion (Fig. 1).

### KASP reaction and PCR conditions

The KASP reaction and its components were as recommended by LGC Genomics Ltd and described at https://www.biosearchtech.com/support/education/kasp-genotyping-reagents/how-does-kasp-work.

Assays were set up as 5 μl reactions in a 384-well format and included 2.5 μl genomic DNA template (15–30 ng of DNA), 2.5 μl of KASP 2 × Master Mix (LGC Genomics), and 0.07 μl primer mix. Primer mix consisted of 12 μl of each tailed primer (100 μM), 30 μl common primer (100 μM) and 46 μl dH_2_O. PCR amplification was performed using an Eppendorf Mastercycler Pro 384 thermal cycler (Eppendorf, UK) using the following programme: hot start at 94 °C for 15 min, followed by ten touchdown cycles (94 °C for 20 s; touchdown from 65 to 57 °C for 1 min, decreasing by 0.8 °C per cycle) and then 30 cycles of amplification (94 °C for 20 s; 57 °C for 1 min). Fluorescent signals from PCR products were read in a PHERAstar microplate reader (BMG LABTECH Ltd.). If tight genotyping clusters were not obtained, an additional 5 cycles (94 °C for 20 s; 57 °C for 1 min) were performed. Genotyping data were analysed using KlusterCaller software (LGC Genomics).

### Low- and high-temperature treatments

Plants were initially grown in a CER at 20 °C (day) and 15 °C (night) with a 16-h photoperiod (lights on between 10:00 and 02:00) and 70% humidity until Zadoks growth stage 39 (Zadoks et al. 1974; Tottman 1987) when the flag leaf ligule is just visible. They were then transferred to plant growth cabinets under continuous light and exposed to low temperatures (13 °C) for 7 days (with 70% humidity) or high temperatures (30 °C) for 24 h (75% humidity). At 20 °C, meiosis takes around 24 h to complete (Bennett et al. 1971, 1973). However, high temperatures speed up meiosis and low temperatures slow it down (Bennett et al. 1972), so the different lengths of the high- and low-temperature treatments ensured that plants would be exposed to temperature treatments during the period from premeiotic interphase to early meiosis I, when they are most sensitive to temperature stress. It also ensured that the anthers to be sampled would have pollen mother cells (PMCs) containing chromosomes at metaphase I at the end of the treatment period so that synapsis and CO formation could be scored. At 30 °C, 24 h is long enough to ensure meiosis progresses to metaphase I but minimises the time available for adverse effects of high temperature on the plants. Longer periods of high-temperature treatment would have made scoring PMCs more difficult as the chromosomes become sticky (Bayliss and Riley 1972a). To prevent dehydration during high-temperature treatment, plant pots were kept in trays of water. For the high-temperature treatments, plants were placed into the treatment cabinets at approximately the same time of day, between 10.00 and 11.45 am.

### Preparation of PMCs for phenotyping

After temperature treatment, anthers were collected from spikes estimated to be undergoing meiosis (when the flag leaf had fully emerged, and the spike length was 4–6 cm long). Anthers were sampled from the first (oldest) 5 tillers only. Using an M80 stereo microscope (Leica Microsystems Ltd., Milton Keynes, UK), anthers were dissected from the two largest florets in each spikelet. Only anthers at metaphase I were scored, so, to determine the meiotic stage, one anther from each floret was stained with acetocarmine and squashed under a cover slip to extrude the PMCs, which were then examined using a DM2000 light microscope (Leica Microsystems). The three anthers within any floret are synchronised in meiotic development, so when PMCs with metaphase I chromosomes were identified, the two remaining anthers from the same floret were fixed in 3:1 (v/v) 100% ethanol/acetic acid, for cytological analysis. Anthers were incubated for at least 24 h at 4 °C before being transferred to 70% ethanol. Fixed anthers were washed with 0.1% sodium dodecyl sulphate for 3–5 min and then hydrolysed with 1 M hydrochloric acid for 10 min at 60 °C. They were then Feulgen-stained with Schiff’s reagent and squashed in 45% acetic acid. This allowed the chromosomes to be spread more widely to facilitate scoring of crossover. Images of metaphase I chromosomes were captured using a DM2000 microscope equipped with a DFC450 camera and controlled by LAS v4.4 system software (Leica Microsystems). For each cell, images were captured in up to 8 different focal planes to aid scoring.

### Cytological analysis of chromosome crossover

**Fig. 2:**
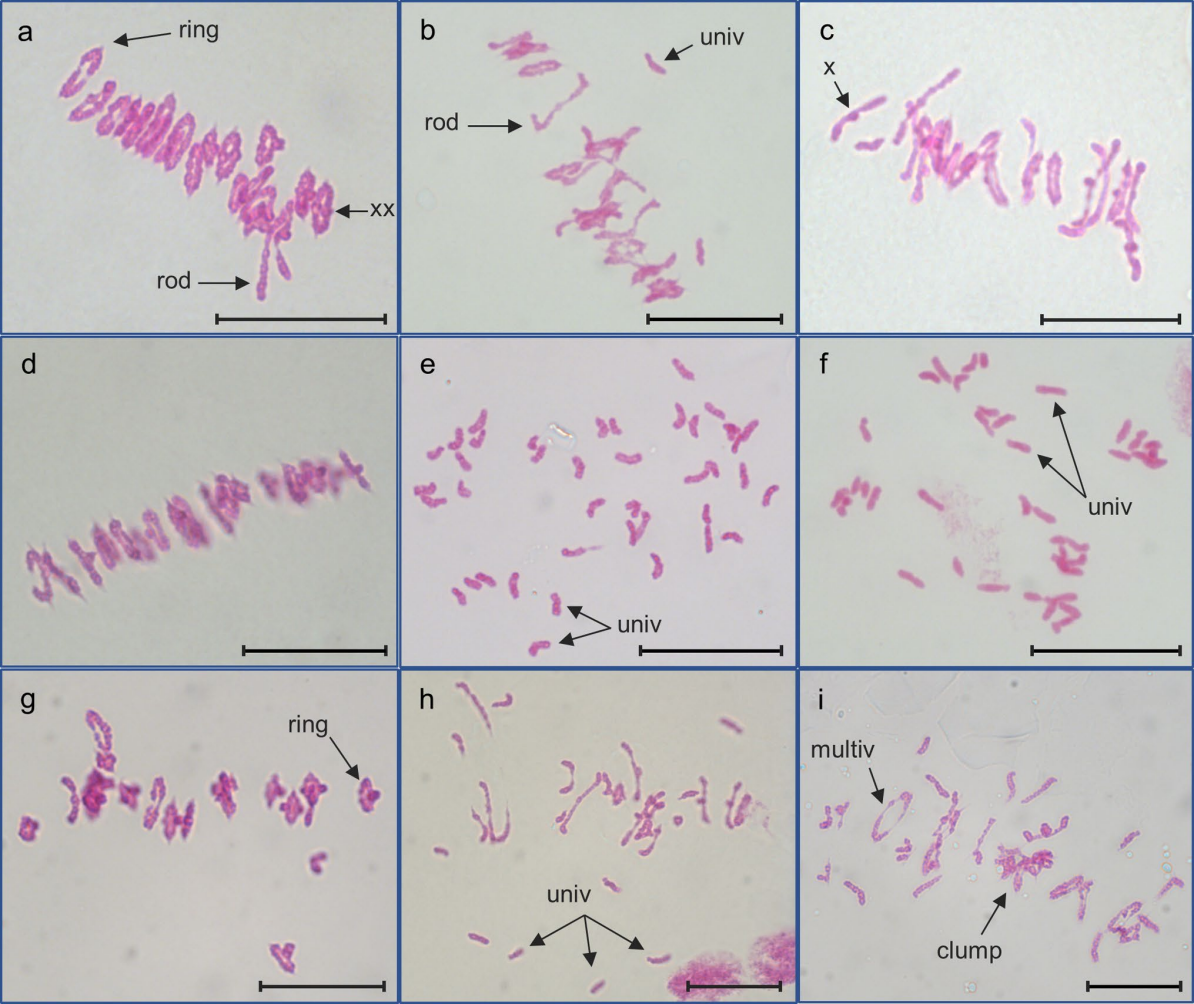
Feulgen-stained metaphase I chromosomes from PMCs of wild-type Chinese Spring, 22-F5 (*ttmei1*) mutant and N5DT5B plants treated at different temperatures. **a** wild type, **b***ttmei1* and **c** N5DT5B at normal temperatures; **d** wild type, **e***ttmei1* and **f** N5DT5B after 7 days at 13 °C; **g** wild type and **h** and **i***ttmei1* after 24 h at 30 °C. Examples of univalent chromosomes (univ), rod bivalents (rod), ring bivalents (ring), multivalents (multiv), single chiasma (X), double chiasmata (XX) and sticky, clumping chromosomes (clump) are indicated with arrows; note complete univalence in *ttmei1* and N5DT5B after treatment at 13 °C. Scale bars, 10 μm

For each plant, 20–30 PMCs were blind scored from digital images. For each cell, the different meiotic chromosome configurations were counted. These were unpaired univalents (0 chiasmata), rod bivalents (1 chiasma), ring bivalents (2 chiasmata), trivalents (2–3 chiasmata), tetravalents (3 chiasmata) and pentavalents (4 chiasmata). Chiasma frequency per PMC was calculated separately for single and double chiasmata (see Fig. 2 for examples of the scored structures).

Statistical analyses were performed using STATISTIX 10.0 software (Analytical Software, Tallahassee, FL, USA). All treatments were analysed by the Kruskal–Wallis test (nonparametric one-way analysis of variance). Means were separated using the Dunn’s test with a probability level of 0.05. Statistical analysis was carried out between temperatures and between genotypes. Bar charts were plotted using Microsoft Excel (2016).

### Immunolocalisation of meiotic proteins ASY1 and ZYP1

To determine when meiosis is disrupted in the low-temperature experiments, PMCs from three wild-type and three *ttmei1* mutant plants were immunolabelled with antibodies against the meiotic proteins ASY1 and ZYP1. PMCs from wild-type Chinese Spring and the *ttmei1* mutant were embedded in acrylamide pads to preserve their 3D structure, and immunolocalisation of ASY1 and ZYP1 was performed as described previously (Martín et al. 2014, 2017). Anti-TaASY1 (Boden et al. 2009) raised in rabbit was used at a dilution of 1:250, and anti-HvZYP1 (Colas et al. 2016) raised in rat was used at a dilution of 1:200. Anti-rabbit Alexa Fluor^®^ 488 and anti-rat Alexa Fluor^®^ 568 (Invitrogen) were used as secondary antibodies.

### Image acquisition and analysis

Images were taken of 30 PMCs from each of the Chinese Spring wild-type and *ttmei1* replicates. Polyacrylamide-embedded PMCs were optically sectioned using a DM5500B microscope (Leica Microsystems) equipped with a Hamamatsu ORCA-FLASH4.0 camera and controlled by Leica LAS-X software v2.0. Z-stacks were deconvolved using Leica LAS-X software. Images were processed using Fiji, which is an implementation of ImageJ, a public domain program available from http://rsb.info.nih.gov/ij/ (Schneider et al. 2012).

### *Dmc1* sequence analysis

Multiple sequence alignment was carried out using ClustalX2 software (Thompson et al. 1997; Larkin et al. 2007). BLAST searches of the *Dmc1* sequences from other plant species against the Chinese Spring IWGSC RefSeq v1.0 sequence assembly (IWGSC 2018) revealed that there are three homeologs of the *Dmc1* gene in hexaploid wheat: TraesCS5A02G133000 (*TaDmc1*-*A1*) on chromosome 5A, TraesCS5B02G131900 (*TaDmc1*-*B1*) on 5B and TraesCS5D02G141200 (*TaDmc1*-*D1*) on 5D. The nucleotide sequences of the DNA, the coding sequences (CDs) and the promoter regions (including 1500 nucleotides downstream of the start codon) of the three *TaDmc1* homeologs were compared.

Multiple DMC1 amino-acid sequence alignment was also carried out between hexaploid wheat (Chinese Spring), barley (*Hordeum vulgare*) and rice (*Oryza sativa* Japonica). Gene IDs from Ensembl Plants are, for barley: HORVU5Hr1G040730.3 (*HvDMC1*) and for rice: Os12g0143800 (*OsDMC1A*) and Os11g0146800 (*OsDMC1B*).

The *Dmc1* sequences from hexaploid wheat (*T. aestivum*) were also compared with those of its diploid and tetraploid progenitors, *T. urartu* (the A-genome donor), *Aegilops tauschii* (the D-genome donor) and *T. dicoccoides* (the AABB tetraploid progenitor). Gene IDs from Ensembl Plants are for *T. urartu* (TuDMC1): TRIUR3_13472; for *Ae. tauschii* (AetDMC1-D): AET5Gv20357200; for the *T. dicoccoides* A copy (TdDMC1-A): TRIDC5AG022500 and for the *T. dicoccoides* B copy (TdDMC1-B): TRIDC5BG023380.

### KASP genotyping of M_3_ plants

KASP genotyping of Chinese Spring wild type and *ttmei1* M_3_ mutant plants was carried out to assess whether the gamma irradiation of Chinese Spring seeds had resulted in a duplication or insertion of *Dmc1*-*D1* in other parts of the genome. Two different sets of *Dmc1*-*D1* chromosome-specific primers were used to genotype three wild type and nine *ttmei1* mutant plants. Primer set 1 consisted of two allele-specific primers: 5′ GAAGGTCGGAGTCAACGGATTcctgtcatgaaaccctgactC 3′ (including HEX tail [wild type]) and 5′ GAAGGTGACCAAGTTCATGCTcctgtcatgaaaccctgactT 3′ (including FAM tail [mutant]), and a common reverse primer: 5′ CcatctgtgctatGcgatAgaA 3′. Primer set 2 consisted of allele-specific primers: 5′ GAAGGTCGGAGTCAACGGATTgtagcttagctcctaaacctCaC 3′ (with HEX tail [wild type]) and 5′ GAAGGTGACCAAGTTCATGCTgtagcttagctcctaaacctCaT 3′ (with FAM tail [mutant]), and common reverse primer: 5′ GcgatAgaAtcttctgaaGtttgtG 3′.

## Results

### Phenotyping 5DL terminal deletion lines at low temperatures

To locate the gene(s) responsible for the *Ltp1* phenotype, we first needed to narrow down our region of interest on chromosome 5DL. To facilitate this, we exposed five Chinese Spring lines with homozygous terminal deletions of 5DL to 13 °C for 7 days. All five had normal chromosome pairing at metaphase I. The breakpoints of these deletion lines were mapped using nine 5D-specific microsatellite markers (Fig. 1). The mapping results were consistent with the order of the breakpoints on the C-banding maps produced by Endo and Gill (1996). Line 5DL-1 had the largest terminal deletion. The breakpoint of this deletion lies between the markers Xcfd40 and Xgdm138, the latter being the most proximal marker to be deleted in this line. We inferred from this that the gene responsible for the *Ltp1* phenotype must lie proximal to Xgdm138. BLAST searches for the Xgdm138 primer sequence against the Chinese Spring IWGSC RefSeq v1.0 sequence assembly revealed its location to be at approximately 391 Mb. The length of chromosome 5D is estimated to be around 565 Mb and the centromere lies at 185.6–188.7 Mb (IWGSC 2018), so we can infer that the length of 5DL is around 376 Mb. The length of the chromosome distal to Xgdm138 is 174 Mb, so almost half of the chromosome arm could be eliminated from our search to find the gene responsible for the *Ltp1* phenotype.

### Identification of a 5DL deletion line with abnormal crossover frequency

To generate deletion lines, 1000 Chinese Spring seeds were gamma irradiated at either 250 or 300 Gy. Of the 500 seeds irradiated at 250 Gy, 409 plants (82%) germinated and 378 plants (76%) were fertile. Of the 500 seeds irradiated at 300 Gy, 417 plants (83%) germinated and 372 (74%) were fertile. M_1_ plants were self-fertilised (four ears per plant where possible), and 1–2 M_2_ seeds from each ear were genotyped. A total of 2444 Chinese Spring M_2_ plants were genotyped, initially using 20 KASP markers located in the proximal half of 5DL. This identified 16 plants with deletions in 5DL (Fig. 1). These deletion lines were all derived from seed irradiated with the higher dose (300 Gy) of gamma irradiation. The largest of these deletions (in the mutant line 21-D11) was estimated to be between 16 and 23 Mb.

**Fig. 3:**
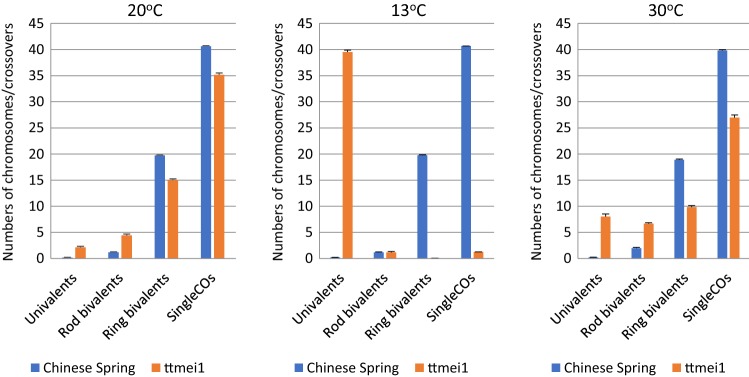
Bar charts showing genotypic effects on meiotic metaphase I chromosomes of Chinese Spring (CS) wild-type and 22-F5 (*ttmei1*) mutant plants after treatment at 20 °C, 13 °C and 30 °C. The numbers of univalents, ring and rod bivalents and single crossovers are shown. Multivalents and double crossovers are not shown

All 16 deletion mutants were exposed to 13 °C for 7 days during premeiotic interphase to early meiosis I. Fifteen plants had normal bivalent formation at metaphase I, but one deletion mutant, 22-F5, exhibited almost complete crossover failure after low-temperature treatment (Fig. 2e; Table 1; Fig. 3, Supplementary Table 1). This is a very similar phenotype to that described for the *Ltp1* locus, so it suggests that *Ltp1* is amongst the genes that have been deleted in the 22-F5 mutant.

**Table 1:** Genotypic effects on meiotic metaphase I chromosomes of Chinese Spring (CS) wild-type and 22-F5 (*ttmei1*) mutant plants after treatment at 20 °C, 13 °C and 30 °C

Genotype	Temperature (°C)	Univalent Mean ± SE	Bivalent (rod) Mean ± SE	Bivalent (ring) Mean ± SE	Trivalent Mean ± SE	Tetravalent Mean ± SE	Pentavalent Mean ± SE	Single CO Mean ± SE	Double CO Mean ± SE
CS wild-type	20	0.17 ± 0.12	1.18 ± 0.19	19.73 ± 0.21	0	0	0	40.66 ± 0.24	43.65 ± 0.32
*ttmeil* mutant	20	2.04 ± 0.38	4.38 ± 0.37	15.04 ± 0.44	0.23 ± 0.08	0.07 ± 0.03	0.03 ± 0.02	35.25 ± 0.51	37.24 ± 0.59
*p* value		0	0	0	0	0	0.0247	0	0
CS wild-type	13	0.24 ± 0.10	1.16 ± 0.25	19.74 ± 0.26	0	0	0	40.65 ± 0.28	43.62 ± 0.38
*ttmeil* mutant	13	39.48 ± 0.34	1.21 ± 0.17	0.05 ± 0.02	0	0	0	1.31 ± 0.18	1.37 ± 0.20
*p* value		0	0.1567	0	–	–	–	0	0
CS wild-type	30	0.21 ± 0.11	2.00 ± 0.25	18.90 ± 0.26	0	0	0	39.79 ± 0.29	42.44 ± 0.34
*ttmeil* mutant	30	7.85 ± 0.66	6.58 ± 0.43	9.98 ± 0.44	0.28 ± 0.08	0.04 ± 0.02	0.01 ± 0.01	27.22 ± 0.55	29.47 ± 0.56
*p* value		0	0	0	0	0.0254	0.3248	0	0

The mean numbers of univalents, ring and rod bivalents and multivalents were scored along with chiasma frequency scored as single and double crossovers (CO). *P* values < 0.05 indicate significant differences

Given the effects of low temperature on the 22-F5 mutant, we assessed whether it also showed reduced tolerance to high temperature. After 24 h at 30 °C, the 22-F5 deletion mutant did indeed exhibit a reduced number of crossovers and increased univalence, but to a lesser extent than at 13 °C (Table 1; Fig. 3; Suppl. Table 1). Therefore, we renamed the 22-F5 deletion mutant ‘*ttmei1*’ to reflect the fact that it has a deletion of an unknown gene ‘*TTmei1*’ (*T*emperature*-T*olerant *mei*osis *1*).

### Crossover in *ttmei1* mutants at normal temperatures

The *ttmei1* mutant plant was self-fertilised and was able to produce M_3_ seed, although seed numbers appeared to be lower than that produced by wild-type plants. Further phenotypic analysis was carried out on Chinese Spring wild type and *ttmei1* M_3_ plants, which were grown under normal temperature conditions or were exposed to 13 °C for 7 days. Five plants were used for each treatment group, and 20–30 PMCs were scored for each plant. Statistical analysis identified differences in synapsis and crossover at different temperatures and between the two different genotypes.

At 20 °C, in wild-type plants, all chromosomes lined up on the metaphase plate as normal, pairing as bivalents, mostly as rings but with the occasional rod bivalent (Fig. 2a). However, at the same temperature, the numbers of univalents, rod bivalents and multivalents were significantly higher in the *ttmei1* mutant when compared with the wild type, and the numbers of ring bivalents and both single and double crossovers were significantly lower (Table 1). Although the proportion of ring and rod bivalents was significantly different in *ttmei1*, the mean number of bivalents (rings + rods) was around 19 as opposed to 21 in the wild type, and the chromosomes were still able to align on the metaphase plate with the exception of the occasional one or two univalent chromosomes (Fig. 2b). At 20 °C, *ttmei1* chromosomes had similar conformations to those observed in N5DT5B plants (Fig. 2c).

### Crossover failure under low temperatures in *ttmei1* mutants

**Fig. 4:**
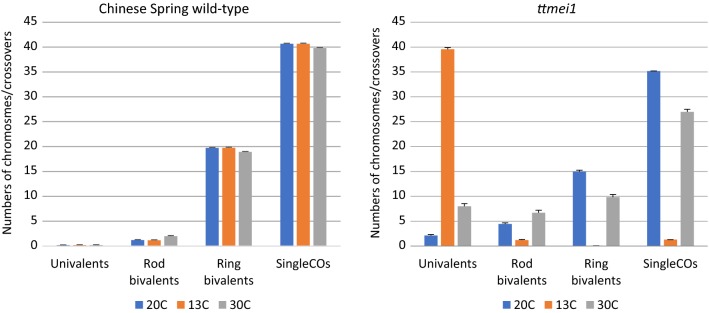
Bar charts showing the effects of three different temperature treatments (20 °C, 13 °C and 30 °C) on meiotic metaphase I chromosomes of Chinese Spring (CS) wild-type and 22-F5 (*ttmei1*) mutant plants. The numbers of univalents, ring and rod bivalents and single crossovers are shown. Multivalents and double crossovers are not shown

The 13 °C treatment had no significant effect on wild-type metaphase I chromosomes (Table 2), which paired mainly as ring bivalents and lined up on the metaphase plate as for normal temperatures (Fig. 2a, d). However, in the *ttmei1* mutant, there were significantly higher numbers of univalent chromosomes at 13 °C than at 20 °C and significantly lower numbers of ring bivalents and crossovers (Table 2; Fig. 4). In *ttmei1* mutant plants at 13 °C, the mean number of univalents per cell was almost 40, whereas in wild-type plants at the same temperature the mean number of univalents was less than one (Table 1; Fig. 3). In fact, in 60% of *ttmei1* PMCs treated at 13 °C, *all* chromosomes were univalent, with 24–41 univalent chromosomes in the remaining 40% of PMCs (data not shown). Univalents appeared more condensed than other chromosomes and were unable to align on the metaphase plate (Fig. 2e). The low chiasma frequency and high numbers of univalent chromosomes in *ttmei1* at low temperatures are very similar in effect to that described when the whole of chromosome 5D is deleted in N5DT5B, as shown in Fig. 2f.

**Table 2:** The effects of three different temperature treatments on meiotic metaphase I chromosomes of Chinese Spring (CS) wild-type and 22-F5 (*ttmei1*) mutant plants

Genotype	Temperature (°C)	Univalent Mean ± SE	Bivalent (rod) Mean ± SE	Bivalent (ring) Mean ± SE	Trivalent Mean ± SE	Tetravalent Mean ± SE	Pentavalent Mean ± SE	Single CO Mean ± SE	Double CO Mean ± SE
CS wild-type	20	0.17 ± 0.12	1.18 ± 0.19^b^	19.73 ± 0.21^a^	0	0	0	40.66 ± 0.24^a^	43.65 ± 0.32^a^
CS wild-type	13	0.24 ± 0.10	1.16 ± 0.25^b^	19.74 ± 0.26^a^	0	0	0	40.65 ± 0.28^a^	43.62 ± 0.38^a^
CS wild-type	30	0.21 ± 0.11	2.00 ± 0.25^a^	18.90 ± 0.26^b^	0	0	0	39.79 ± 0.29^b^	42.44 ± 0.34^b^
*p* value		0.9964	0	0	–	–	–	0	0
*ttmeil* mutant	20	2.04 ± 0.38^c^	4.38 ± 0.37^b^	15.04 ± 0.44^a^	0.23 ± 0.08^a^	0.07 ± 0.03^a^	0.03 ± 0.02	35.25 ± 0.51^a^	37.24 ± 0.59^a^
*ttmeil* mutant	13	39.48 ± 0.34^a^	1.21 ± 0.17^c^	0.05 ± 0.02^c^	0^b^	0^c^	0	1.31 ± 0.18^c^	1.37 ± 0.20^c^
*ttmeil* mutant	30	7.85 ± 0.66^b^	6.58 ± 0.43^a^	9.98 ± 0.44^b^	0.28 ± 0.08^a^	0.04 ± 0.02^b^	0.01 ± 0.01	27.22 ± 0.55^b^	29.47 ± 0.56^b^
*p* value		0	0	0	0	0.0247	0.1121	0	0

The numbers of univalents, ring and rod bivalents and multivalents were scored along with chiasma frequency scored as single and double crossovers (CO). *P* values < 0.05 indicate where there are significant differences in the scores between different temperature treatments. Superscript letters a, b and c indicate where the significant differences lie. For scores with the same letter, the difference between the means is not statistically significant. If the scores have different letters, they are significantly different

### Immunolabelling of PMCs at low temperatures

**Fig. 5:**
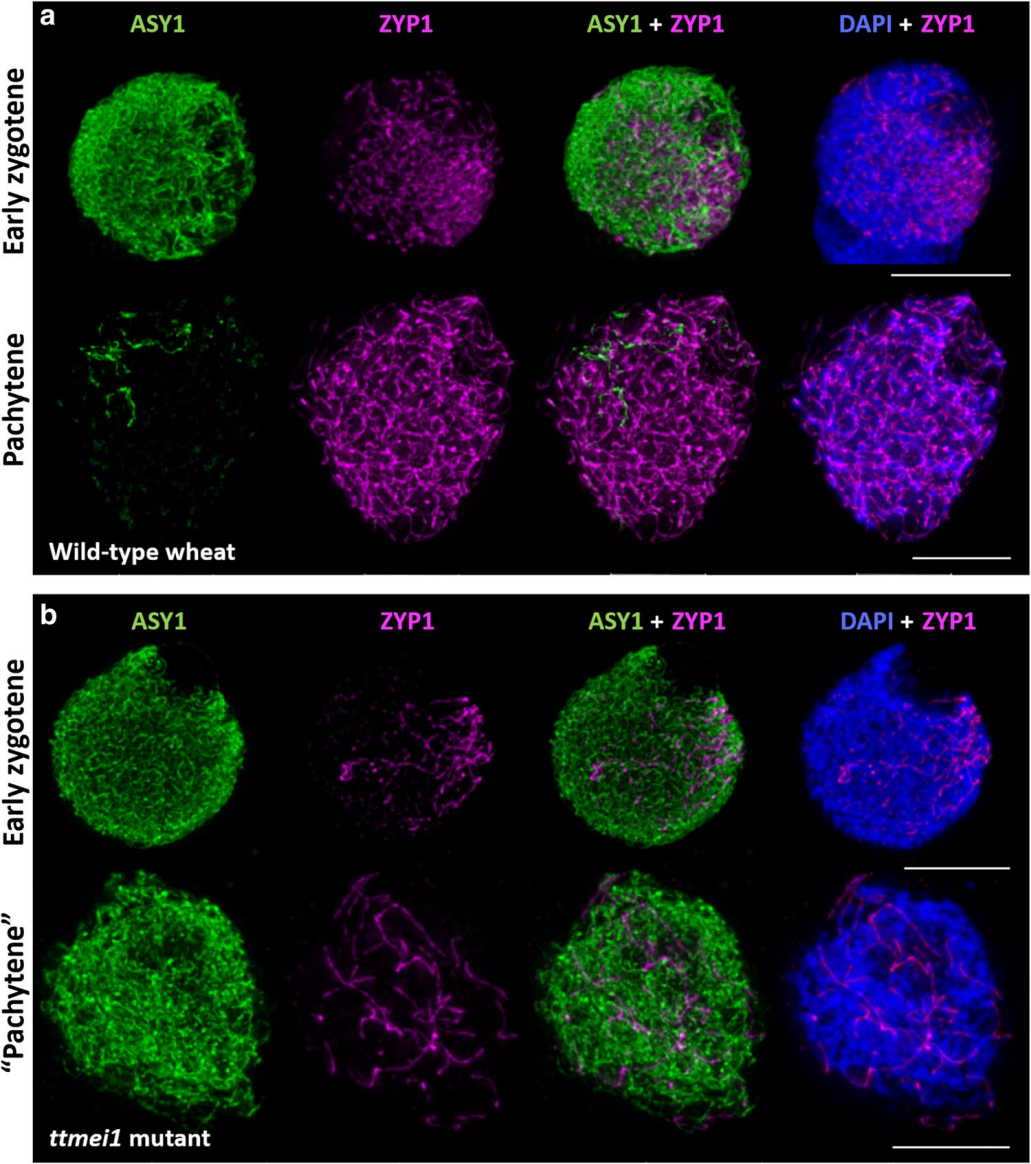
Immunolocalisation of meiotic proteins ASY1 (green) and ZYP1 (magenta) in PMCs from wheat Chinese Spring wild type (**a**) and *ttmei1* mutant (**b**), both under low-temperature conditions. **a** During early zygotene, synapsis starts from the telomeres at one pole of the nucleus in wild-type wheat. During pachytene, all chromosomes have synapsed. ASY1 labelling in green shows that a very small proportion of chromatin has not yet synapsed. **b** In *ttmei1* mutants, synapsis initiates normally at one pole of the nucleus; however, synapsis is soon compromised and is not completed. Therefore, there is no normal pachytene in the *ttmei1* mutant. DAPI staining in blue. Scale bar, 10 μm

To investigate when meiosis was being disrupted in the low-temperature experiments, wild-type and *ttmei1* mutant PMCs were immunolabelled with antibodies against the meiotic proteins ASY1 and ZYP1 to follow the progression of synapsis (Fig. 5). ASY1 is part of the lateral elements of the SC and first appears during premeiotic interphase, before synapsis begins (Armstrong et al. 2002; Boden et al. 2009). ZYP1 is part of the central region of the SC, which assembles between the lateral elements (Higgins et al. 2005; Khoo et al. 2012), and it is present only where chromosomes are synapsed. Immunolabelling therefore gives an indication of the level of synapsis achieved at pachytene when the SC is fully established, which enables tracking of the progression of synapsis. Clear differences were detected when comparing ASY1 (green) and ZYP1 (magenta) signals in the wild type and *ttmei1* mutant at low temperatures. In wild-type wheat (Fig. 5a), synapsis proceeds in the same way as it does at normal temperatures: It starts from the telomeres at one pole of the nucleus during early zygotene, and, by pachytene, all chromosomes have synapsed. A very small amount of ASY1 labelling is still visible at pachytene, indicating the small amount of chromatin that has not yet synapsed. In *ttmei1* mutants (Fig. 5b), at early zygotene, ASY1 localises to the chromosome axes in a pattern similar to that observed in wild-type wheat, and synapsis initiates normally at one pole of the nucleus. After early zygotene, chromatin staining with DAPI (4′,6-diamidino-2-phenylindole) suggests that the *ttmei1* PMCs are at pachytene (Fig. 5b), but the amount of ZYP1 labelling is much lower than in pachytene in the wild type, indicating that synapsis has been compromised and is not completed. None of the PMCs had a completed synapsis in the *ttmei1* mutants.

### Gene content in the *ttmei1* deletion region

The *ttmei1* deletion was initially mapped to a 16-Mb region of 5DL, between the flanking markers BA00334971 and BA00808441 (Fig. 1). In this initial analysis, only one KASP marker, BA00822801, was deleted in *ttmei1*. KASP genotyping of *ttmei1* using 25 more markers between BA00334971 and BA00808441 fine-mapped the deletion to a 4-Mb region between BA00798994 and BA00124919 (Fig. 1). Seven markers were deleted in this region.

The gene content within the 4-Mb deletion regions in *ttmei1* was revealed using data derived from the hexaploid wheat gene annotation v1.1 (IWGSC 2018) available from Ensembl Plants. Functional annotations of the genes were retrieved from the file ‘FunctionalAnnotation.rds’ in https://opendata.earlham.ac.uk/wheat/under_license/toronto/Ramirez-Gonzalez_etal_2018-06025-Transcriptome-Landscape/data/TablesForExploration/FunctionalAnnotation.rds (Ramírez-González et al. 2018). Expression patterns of all the genes within the *ttmei1* deletion region were investigated based on 876 RNASeq samples from different tissue types available in the wheat expression browser http://www.wheat-expression.com. A total of 41 genes (16 high confidence and 25 low confidence genes) were identified in the 4-Mb deletion region. Eighteen of the 41 genes were expressed during meiosis (Table 3). We considered a gene to be expressed when its expression level was > 0.5 transcripts per million (TPM) in at least one meiotic sample (Alabdullah et al. 2019). Twelve of these eighteen genes are high confidence genes. Detailed expression data (TPM) for all genes in the *ttmei1* deletion region are shown in Supplementary Table 2.

**Table 3:** Genes expressed during meiosis within the 4-Mb deletion region of *ttmei1*. *TaDmc1*-*D1* is shown in bold

Gene ID	Gene position		Average expression (TPM)					Gene annotation
	Start	End	Meiosis (*n* = 17)	Spike (*n* = 278)	Grain (*n* = 119)	Leaves/Shoots (*n* = 403)	Roots (*n* = 50)	
TraesCS5D02G140400	223955756	223961791	13.3	8.0	5.6	6.5	10.4	L-galactono-1,4-lactone dehydrogenase
TraesCS5D02G140600	224313206	224316322	2.7	2.5	0.5	1.3	2.4	Embryogenesis transmembrane protein-like
TraesCS5D02G140800	224350841	224352008	1.2	1.0	0.5	0.5	0.7	DTW domain containing protein
TraesCS5D02G140900	224760012	224761795	0.7	3.0	0.2	3.9	2.6	Glycosyltransferase
TraesCS5D02G211900LC	224760203	224761141	0.6	1.5	0.1	1.9	0.7	mRNA decapping complex subunit 2
TraesCS5D02G141000	224869609	224873145	5.0	3.8	2.0	1.4	2.1	Set1/Ash2 histone methyltransferase complex subunit ASH2
TraesCS5D02G141100	224941459	224944119	41.8	69.4	49.0	50.4	41.8	Ubiquitin-like protein 5
**TraesCS5D02G141200**	**224946636**	**224950905**	26.9	4.7	2.2	2.7	1.7	**Disrupted meiotic cDNA 1 protein (TaDMC1-D1)**
TraesCS5D02G141300	225245032	225260448	12.3	13.9	6.6	18.2	11.0	Long-Chain Acyl-CoA Synthetase
TraesCS5D02G212200LC	225256993	225257817	0.3	0.7	0.3	0.5	0.2	Retrotransposon protein, putative, LINE subclass
TraesCS5D02G212300LC	225257900	225258319	0.5	1.0	0.6	0.9	0.5	RNA-directed DNA polymerase (reverse transcriptase)-related family protein
TraesCS5D02G141400	225581642	225584961	11.1	16.7	12.2	15.5	17.6	R3H domain-containing protein 4
TraesCS5D02G141600	226939574	226942436	7.6	9.2	3.8	3.1	5.9	Heavy metal transport/detoxification superfamily protein
TraesCS5D02G213200LC	226939602	226942394	5.7	5.4	2.8	3.0	8.0	ACP-SH:acetate ligase
TraesCS5D02G141800	227703274	227705102	8.1	0.0	0.0	0.0	0.0	Actin cross-linking protein (DUF569)
TraesCS5D02G141900	227736852	227737403	0.6	5.3	0.9	6.6	9.8	Calmodulin-like family protein
TraesCS5D02G213600LC	227771092	227771409	9.5	8.7	2.4	3.7	9.6	Pentatricopeptide repeat (PPR-like) superfamily protein
TraesCS5D02G213610LC	228053904	228057422	3.1	2.8	1.3	1.4	2.2	Protein FAR 1 -RELATED SEQUENCE 5

Both high confidence and low confidence (LC) genes are included. A total of 867 RNASeq samples from different tissue types, including 17 meiotic anther samples, were used to determine the expression pattern of the genes. A gene was defined as being expressed during meiosis when its expression level > 0.5 TPM (transcripts per million) in at least one meiotic anther sample. Gene position is according to the Chinese Spring IWGSC RefSeq v1.1 gene annotation and was retrieved from Ensembl Plants

### *TaDmc1*-*D1* is a candidate for the *Ltp1* phenotype

Of the 18 genes expressed during meiosis in the *ttmei1* deletion interval, the strongest candidate for the *TTmei1* phenotype is TraesCS5D02G141200, which is the D-genome homeolog of *Dmc1* (*Disrupted meiotic cDNA 1*), which has a known meiotic phenotype, playing a central role in homologous recombination. It is a high confidence gene, and its expression level is around ten times higher in meiotically active tissues compared with non-meiotic tissues (averages 26.9 TPM and 2.8 TPM, respectively [Suppl. Fig. 2]). The other 17 genes in the region are much less likely than *TaDmc1* to be candidates for the *TTmei1* phenotype because none of them could be attributed to any known meiotic function, and although they are expressed during meiosis, expression is proportionally higher in other tissues.

To assess whether gamma irradiation of the Chinese Spring seeds had resulted in a duplication or insertion of *Dmc1*-*D1* in other parts of the genome, three Chinese Spring wild-type and nine *ttmei1* M_3_ mutant plants were KASP-genotyped using two different sets of *Dmc1*-*D1-*specific primers. As expected, with both primer sets, none of the *ttmei1* mutant plants showed any amplification when compared with the wild-type plants. This indicates very clearly that *Dmc1*-*D1* is completely deleted from the *ttmei1* deletion line and has not been duplicated or inserted elsewhere in the genome as a result of the gamma irradiation.

BLAST searches against the Chinese Spring IWGSC RefSeq v1.0 sequence assembly (IWGSC 2018) revealed that there are three homeologs of the *Dmc1* gene in hexaploid wheat: TraesCS5A02G133000 (*TaDmc1*-*A1*) on chromosome 5A, TraesCS5B02G131900 (*TaDmc1*-*B1*) on 5B and TraesCS5D02G141200 (*TaDmc1*-*D1*) on 5D. There are differences in gene expression levels between the three *Dmc1* homeologs in meiotic anthers: *TaDmc1*-*A1* has the lowest gene expression levels and *TaDmc1*-*D1* the highest (Suppl. Fig. 1).

### *Dmc1* sequence analysis

The nucleotide sequences of the DNA, the coding sequences (CDs) and the promoter regions of the three *TaDmc1* homeologs were compared. The CDs of the A, B and D homeologs are highly conserved (Suppl. Fig. 2) with sequence identity ranging between 98.1 and 98.7% (Suppl. Table 3a). The DNA sequences are less similar (Suppl. Fig. 3), with sequence identity ranging from 82.1% (between A and B homeologs) to 89.0% (between A and D homeologs) (Suppl. Table 3a). Comparing the promoter regions of each of the three *TaDmc1* homeologs showed a large insertion mutation (163 nt in size) at position 430 downstream of the start codon of *TaDmc1*-*B1* (Suppl. Fig. 4).

**Fig. 6:**
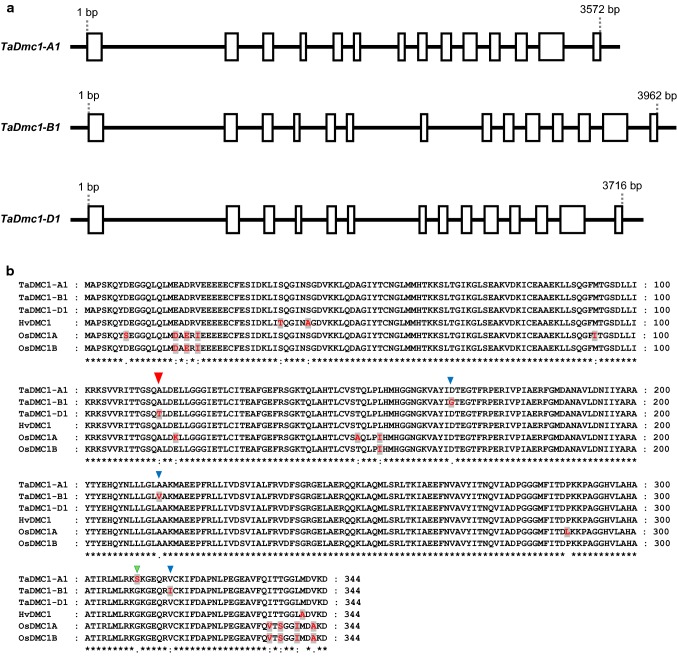
*TaDmc1* gene structure and amino-acid sequence alignment. **a** Exon–intron structure of the three *TaDmc1* homeologs. **b** Multiple sequence alignment of amino acids from DMC1 proteins of different cereal plants: hexaploid wheat (*Triticum aestivum* cv Chinese Spring; Ta), barley (*Hordeum vulgare*; Hv) and rice (*Oryza sativa* Japonica; Os). SNPs are shown in red text on a grey background. Symbols below each position in the sequence indicate the amount of conservation (asterisk ‘*’: identical residues; colon ‘:’: conserved substitution; period ‘.’: semi-conserved substitution; and space ‘ ’: not conserved). A single amino-acid substitution in TaDMC1-D1 at position 114 (indicated by a large red triangle) may confer low temperature tolerance in wheat. Small blue triangles show the positions of three other amino-acid substitutions in TaDMC1-B1 at positions 167, 214 and 316, and a small green triangle shows the position of a substitution in TaDMC1-A1 at position 310

The exon–intron structure of the three *TaDmc1* homeologs (Fig. 6a) is conserved in terms of number of exons (14 exons each); however, there is a variation in intron length between the three homeologs due to large indel mutations mainly in introns 3, 6 and 7 (data not shown). *TaDmc1* homeologs have no splice variants. In all three *TaDmc1* homeologs, there is an open reading frame of 1035 bp (Suppl. Fig. 2), which encodes a predicted protein of 344 amino acids (Fig. 6b). This protein is highly conserved between the three homeologs. There are five single amino-acid substitutions between the wheat homeologs, but only one single amino-acid substitution (at position 114) in TaDMC1-D1 (threonine) when compared with TaDMC1-A1 and TaDMC1-B1 (alanine) (Fig. 6b).

Multiple DMC1 amino-acid sequence alignment was also carried out between the three hexaploid wheat homeologs and barley (*Hordeum vulgare*) and rice (*Oryza sativa* Japonica). There is a high level of amino-acid sequence conservation between the wheat, barley and rice proteins (Fig. 6b) with sequence identity ranging between 95.3 and 99.4% (Suppl. Table 3b). This conservation of amino-acid sequence between the wheat, barley and rice DMC1 proteins has also been reported by Colas et al. (2019).

*Dmc1* sequence comparisons were also made between hexaploid wheat (*T. aestivum*) and its diploid and tetraploid progenitors, *T. urartu* (the A-genome donor), *Ae. tauschii* (the D-genome donor) and *T. dicoccoides* (the AABB tetraploid progenitor) (Suppl. Fig. 5). The *Dmc1* sequences of *T. aestivum* are quite conserved when comparing each gene copy with its ancestral genome copy. For the DNA sequences, the highest percentage of homology is between the D copy of *T. aestivum* and the ancestral gene of *Ae. tauschii* (99.8%), whilst the lowest homology is between the B copy of *T. aestivum* and the B-genome copy in *T. dicoccoides* (92.2%) (Suppl. Table 3c), due to a large insertion of 294 bp in intron 7 (Suppl. Fig. 5, Suppl. Fig. 6). The percentages of homology in *TaDmc1*-*A1* versus *T. dicoccoides*_A and *TaDmc1*-*A1* versus *T. urartu* are 98.6% and 98.4%, respectively (Suppl. Table 3c).

Comparisons were also made between the amino-acid sequences of DMC1 proteins from *T. aestivum* and those of its diploid and tetraploid progenitors (Suppl. Fig. 7). Unlike those of *T. aestivum*, most of the *Dmc1* gene copies in the wheat ancestors have multiple transcripts due to alternative splicing variants (as predicted by Ensembl Plants). *TdDmc1*-*A* (*T. dicoccoides* A copy) has 3 transcripts; *TdDmc1*-*B* (*T. dicoccoides* B copy) has 9 transcripts; and *AetDmc1*-*D* (*Ae. tauschii*) has 16 transcripts. *TuDmc1*-*A* (*T. urartu*, TRIUR3_13472) has a single transcript, but of all the ancestral DMC1 proteins in our comparisons, the encoded protein sequence of TuDMC1 from *T. urartu* is the one that diverges most from that of *T. aestivum* (Suppl. Table 3d). Of the amino-acid differences between the A-, B- and D-genome copies of DMC1 in *T. aestivum*, the single amino-acid substitution in the D-genome copy of *T. aestivum* at position 114 (Fig. 6) is also present in the D-genome ancestor, and the A-genome substitution at position 310 is also present in the A-genome ancestor, whereas the B-genome substitutions at positions 167, 214 and 316 are different to all of the ancestral copies in *T. dicoccoides* (Suppl. Fig. 7).

### Crossover failure under high temperatures in *ttmei1* mutants

Five wild-type and 11 *ttmei1* M_3_ plants were exposed to 30 °C for 24 h. A higher number of mutant plants were treated because of the wide variation in the data for this group. Chromosome stickiness and clumping were observed in many of the cells at this temperature (Fig. 2i), which made scoring more difficult than at normal or low temperatures. In wild-type plants, high temperature resulted in significantly higher numbers of rod bivalents and significantly lower numbers of ring bivalents and crossovers than had been observed at 20 °C (Figs. 2g, 4; Table 2). However, these differences were much more pronounced in the *ttmei1* mutant. In *ttmei1* mutant plants, there were significantly higher numbers of univalents, rod bivalents and tetravalents at 30 °C than at normal temperatures and significantly fewer ring bivalents and crossovers although the level of univalence was not nearly as pronounced as that at 13 °C (Table 2; Fig. 4). There were also significant differences between the two genotypes at 30 °C. For example, the mean number of univalents was 7.85 in *ttmei1* mutants but only 0.21 in wild-type plants (Table 1; Fig. 3). It was also observed that many *ttmei1* chromosomes did not align correctly on the metaphase plate (Fig. 2h, i).

## Discussion

Chinese Spring wild-type plants and mutant plants with interstitial deletions within chromosome 5DL were exposed to low temperature (13 °C) for 7 days during a period lasting from premeiotic interphase to early meiosis I. Microscopic examination of PMCs from these plants enabled us to identify a 5DL deletion mutant whose meiotic chromosomes exhibit extremely high levels of asynapsis and chromosome univalence. Within the deleted region of this mutant, we identified a candidate gene on 5DL (*TaDmc1*-*D1*) that is probably responsible for the *Ltp1* phenotype. Subsequent exposure of this *ltp1* deletion mutant to 24 h of high temperature (30 °C) during the same developmental period led to a reduced number of crossovers and increased univalence, though to a lesser extent than at 13 °C. As this deletion clearly has an effect on chromosome pairing at 30 °C as well as at 13 °C, the mutant line was renamed *ttmei1* to reflect both its reduced high temperature tolerance and its loss of *Ltp1*.

By exploiting a series of wheat 5DL terminal deletion lines, the *Ltp1* locus was initially delimited to the proximal half of chromosome 5DL. Using terminal deletion lines to narrow down our region of interest proved to be a good strategy as we were able to eliminate almost the entire distal half of the chromosome from our investigations. The deletion mapping was refined by screening a population of around 2500 gamma-irradiated plants using 5DL-specific KASP markers. Of these, sixteen plants had 5DL deletions. The resources now available, including the Chinese Spring RefSeq v1.0 genomic sequence assembly, KASP primers from www.cerealsdb.uk.net and the Ensembl Plants database, made the screening and mapping processes much easier and quicker than when a similar approach was used to map the *Ph1* locus (Griffiths et al. 2006).

Exposure of the 5DL mutant plants to 13 °C for 7 days identified a deletion mutant, *ttmei1*, that exhibits extremely high levels of chromosome univalence at low temperatures. This is very similar to the *Ltp1* phenotype previously described in N5DT5B plants where the whole of chromosome 5D is missing. When grown under normal conditions, *ttmei1* plants are not sterile and are able to produce seed, however, seed numbers appeared to be lower than in the wild type. In *ttmei1* mutants, chromosome synapsis and crossover are only slightly disrupted at 20 °C, but multivalents were observed in four of the *ttmei1* mutants at this temperature, which could indicate the presence of additional rearrangements that might alter chromosome pairing. However, in one mutant plant, *ttmei1*-*4*, no multivalents were observed (Suppl. Table 1), suggesting that the *ttmei1* deletion is not necessarily linked to the occurrence of multivalents. The presence of high numbers of univalents and more rod bivalents than normal in *ttmei1* mutants under low temperatures suggests a major problem with CO formation. Consistent with this, the *ttmei1* line exhibits significant abnormalities of synapsis at low temperatures.

The *ttmei1* mutant carries a 4-Mb deletion of 5DL. Within this deletion interval, there are 41 genes, 18 of which are expressed during meiosis. Amongst these genes, the strongest candidate for the low-temperature pairing phenotype is the meiotic recombination gene *TaDmc1*-*D1*. None of the other genes are known to have any meiotic function. Moreover, *TaDmc1* is expressed most highly during early prophase I (Suppl. Table 2), which, in wheat, is when synapsis is initiated at the ‘telomere bouquet’ stage (Martín et al. 2017). *TaDmc1*-*D1* is therefore a strong candidate for temperature tolerance at low temperatures.

DMC1 plays a central role in homologous recombination, SC formation and cell-cycle progression. It is a meiosis-specific protein, structurally similar to the bacterial strand exchange protein RECA (Bishop et al. 1992). The control of DNA strand exchange during meiotic recombination is vital for sexual reproduction. Homologous recombination is initiated by the programmed formation of DNA double-strand breaks (DSBs) at leptotene. DMC1 and RAD51 (another RECA homolog) form filaments on the single-strand DNA overhangs at DSBs. These filaments facilitate homology search and catalyse strand invasion and strand exchange between homologous chromosomes (Neale and Keeney 2006). Repair of these interhomolog invasion events results in crossovers or non-crossovers (Reviewed in Lambing et al. 2017).

*Dmc1* homologs are found in a wide variety of organisms. In yeast, *dmc1* mutants have defects in reciprocal recombination, fail to form normal SCs, accumulate DSB recombination intermediates and the cells arrest in late meiotic prophase (Bishop et al. 1992). In mice, *dmc1* mutants have defective synapsis which leads to severe sterility also due to prophase arrest (Pittman et al. 1998; Yoshida et al. 1998; Bannister et al. 2007). In our wheat experiments, immunolocalisation of ASY1 and ZYP1 showed that at 13 °C synapsis was compromised in *ttmei1* mutants and was not completed, with meiosis appearing to arrest before pachytene in late prophase. This is similar to the phenotypes observed in yeast and mice at ambient temperatures.

In most diploid plant species, deletion of *Dmc1* also leads to sterility (Devisetty 2010). However, in *Arabidopsis thaliana*, *atdmc1* mutants have a disrupted synapsis with high levels of univalence, resulting in abnormal pollen grain formation and reduced fertility, but plants are not completely sterile because random chromosome segregation allows enough PMCs to reach maturity (Couteau et al. 1999). The Arabidopsis genome contains one copy of the *Dmc1* gene. In rice (*Oryza sativa*), there are two *Dmc1* homologs, *OsDMC1A* and *OsDMC1B,* that probably arose through chromosome duplication (Ding et al. 2001). A mutation in one or other of these homologs does not cause problems in meiosis, but *Osdmc1a Osdmc1b* double mutants exhibit serious CO defects, abnormal synapsis, high numbers of univalents at metaphase and are sterile (Wang et al. 2016). Barley (*Hordeum vulgare*) carries a single *Dmc1* homolog, *HvDMC1*, and mutations in this gene lead to abnormal synapsis, multiple univalents and chromosome mis-segregation (Colas et al. 2019; Szurman-Zubrzycka et al. 2019). Thus, it appears that disruption of the barley orthologue of *Dmc1* at normal temperatures leads to a phenotype similar to that of *ttmei1* at low temperatures.

Previous evidence from animal studies supports our suggestion that *Dmc1* is involved in the maintenance of normal chromosome synapsis at low temperatures. In the Japanese red-bellied newt, *Cynops pyrrhogaster,* which is a poikilothermic amphibian, low temperatures during meiosis impair synapsis/recombination, which results in the production of abnormal spermatozoa (Yazawa et al. 2003). In newts incubated at 8 °C, spermatogenesis does not proceed beyond meiotic metaphase I, and univalent chromosomes are observed, indicating chromosome pairing failure as a result of asynapsis. At 12 °C, meiotic chromosomes have similar, though less severe, abnormalities. Under the same low-temperature conditions (8 °C or 12 °C), expression of the DMC1 protein decreases, leading to the suggestion that its low expression level contributes to the temperature-dependent abnormalities in spermatogenesis.

In hexaploid wheat, there are three copies of *Dmc1*: *TaDmc1*-*A1* on 5A, *TaDmc1*-*B1* on 5B and *TaDmc1*-*D1* on 5D, and differences in the abilities of these three genes to stabilise the genome at low temperatures could also be related to differences in gene expression. In the hexaploid wheat variety ‘Highbury’, there are differences in the levels of gene expression between the three *Dmc1* homeologs during premeiosis and early meiosis, which suggests that they might contribute to meiotic recombination to differing extents (Devisetty et al. 2010). This is reflected in our own study in Chinese Spring, where, in meiotic anthers, gene expression levels are highest in *TaDmc1*-*D1*, intermediate in *TaDmc1*-*B1* and lowest in *TaDmc1*-*A1* (Suppl. Fig. 1). In Chinese Spring, deletion of the region containing *TaDmc1*-*D1* results in asynapsis at 13 °C, and it seems highly likely that this gene promotes low temperature tolerance. However, different dosages of the *TaDmc1* alleles can affect the stability of synapsis at low temperatures. When chromosome 5A is present as a single dose in N5DT5B plants, it cannot compensate for the lack of 5D and is unable to stabilise synapsis at low temperatures, but when present as a double dose in N5DT5A plants it can compensate for the 5D deficiency and chromosome pairing at 12 °C is normal (Riley et al. 1966). The A-genome homeolog, *TaDmc1*-*A1,* has the lowest expression of the three copies of *Dmc1* in Chinese Spring. This suggests that when *Dmc1*-*A1* is present at a double dose, an increase in its expression compensates for the loss of *Dmc1*-*D1* to provide low temperature tolerance. There is only one amino-acid difference between the TaDMC1-A1 and TaDMC1-D1 proteins (Fig. 6b), a substitution of alanine (a non-polar amino acid) in TaDMC1-A1 (and TaDMC1-B1) by threonine (a polar amino acid) in TaDMC1-D1 at position 114. The threonine residue is also present in the ancestral genome of *Ae. tauschii* (Suppl. Fig. 7). The fact that TaDMC1-A1 can compensate for TaDMC1-D1 suggests that this amino-acid difference is not so important for low temperature tolerance.

The A- and D-genome copies of *TaDmc1* are highly conserved with their ancestral homeologs, which suggests that these copies are functional. The B-genome copy is less well conserved due to a large insertion of 294 bp in intron 7 (Suppl. Fig. 5; Suppl. Fig. 6). *Dmc1*-*B1* has intermediate gene expression in Chinese Spring, higher than *Dmc1*-*A1*, but lower than *Dmc1*-*D1*, but it cannot compensate for the loss of the D copy at low temperatures even when present as a double dose. The inability of *Dmc1*-*B1* to stabilise low-temperature pairing may arise from three amino-acid differences in TaDMC-B1 compared to the A- and D-genome copies, or from the 163-bp insertion in the promoter region of *TaDmc1*-*B1*. Alternatively, the 294-bp insertion in intron 7 of *TaDmc1*-*B1* could be affecting gene expression, because although intronic sequences do not encode protein products, they can still have an effect on gene expression and gene functionality (Mattick and Makunin 2006; Rearick et al. 2011). It is important that breeders are aware that there could be *TaDmc1* alleles in their wheat germplasm that cannot stabilise wheat synapsis at extremes of temperature and that the effects of these alleles may be masked by the temperature-stabilising actions of TaDMC1-D1.

In tetraploid wheats (AABB), synapsis at 12 °C is normal despite the absence of chromosome 5D (Riley et al. 1966). This may be due to the presence of a dominant *Ltp* allele on chromosome 5A that duplicates the chromosome stabilising activity of 5D in hexaploid wheat (Hayter and Riley 1967). Interestingly, a previous study has shown that some other varieties and subspecies of wheat differ from Chinese Spring in that the gene that stabilises chromosome pairing at low temperatures is located on a chromosome other than 5D (Chapman and Miller 1981). In the wheat variety ‘Hope’ and the subspecies *spelta*, the dominant *Ltp* gene is located on chromosome 5A rather than 5D.

It has also been postulated that there are additional *Ltp* genes on chromosomes 5AS (*Ltp2*) and 5BS (*Ltp3*) that also have a stabilising effect on homologous pairing and crossover at low temperatures (Queiroz et al. 1991), but high levels of univalence and low chiasma frequencies were only seen after 20 days of cold treatment and at a slightly lower temperature (10 °C).

To determine whether *TaDmc1*-*D1* is the gene responsible for stabilising the low-temperature asynapsis observed in Chinese Spring plants with 5D chromosomal deletions, the next step will be to either disrupt or delete the gene and then assess the resulting mutant plants for low-temperature asynapsis. This could potentially be done either using Targeting Induced Local Lesions IN Genomes (TILLING) populations or by CRISPR/Cas9 genome editing. TILLING lines are available in the hexaploid wheat variety ‘Cadenza’ but are not yet available for Chinese Spring. Moreover, results from recent experiments to edit a gene within the *Ph1* locus suggest that CRISPR may be a better choice for deleting the *TaDmc1*-*D1* gene. Like *Ltp1,* the *Ph1* locus, on wheat chromosome 5B, is scored by its deletion phenotype. This locus, which affects both chromosome pairing and crossover, contains the major wheat meiotic crossover gene *ZIP4*. When *zip4* CRISPR deletion mutants were compared with the *ph1b* deletion mutant (Sears 1977), they exhibited a similarly high level of homeologous crossover and a similar effect on homologous pairing (Rey et al. 2018). Whereas, when Cadenza *zip4* TILLING mutants were compared to the *ph1b* mutant, although they exhibited a similarly high level of homeologous crossover, only some exhibited a similar effect on homologous pairing (Rey et al. 2017). Therefore, we are currently investigating the possibility of generating *TaDmc1*-*D1* Chinese Spring deletion mutants using CRISPR/Cas9 genome editing.

In *ttmei1* mutants, aberrant chiasma formation was also observed after 24 h at 30 °C, though this was not as pronounced as in the low-temperature phenotype. So, if *TaDmc1*-*D1* is the gene responsible for the observed low temperature tolerance, it may well also have a stabilising effect at high temperatures, though to a lesser extent than at low temperatures. There is high variation in the scores for chiasma frequency between the 11 *ttmei1* plants treated at 30 °C. One explanation for this variation could be that the faster progression of meiosis at high temperature may make it harder to target the stages most sensitive to high temperatures. Plants were placed into the 30 °C treatment cabinets at slightly different times of day (10.00–11.45 am), so that high-temperature treatment may have coincided with temperature-sensitive stages of meiosis or premeiosis to a greater or lesser extent.

However, it is also likely that *ttemi1* mutants carry many duplications and deletions in addition to those we have identified on 5DL, particularly since the *ttmei1* mutant plants were derived from seed irradiated with the higher dose (300 Gy) of gamma irradiation. We have determined through KASP genotyping that *Dmc1*-*D1* has not been duplicated or inserted elsewhere in the genome, but it is possible that *ttmei1* mutants carry a background deletion that has not yet been detected that contains a different meiotic recombination gene affecting high temperature tolerance that is segregating with the *ttmei1* deletion. However, all 11 of the *ttemi1* mutants scored at high temperatures exhibited reduced crossover, suggesting that the *ttemi1* deletion is associated with reduced high temperature tolerance. We are currently conducting further experiments to understand this variation at high temperature. We are also backcrossing *ttmei1* mutants with Chinese Spring wild-type plants to remove background deletions.

### Implications for plant breeding

In the future, breeders face the challenge of producing crops with increasing resilience to environmental stress whilst producing ever higher yields (Steinmeyer et al. 2013). Global climate models predict that temperatures will continue to increase over the twenty-first century and that high-temperature extremes such as heat waves are likely to occur with a higher frequency and duration (Collins et al. 2013). It has been estimated that if global temperatures increase by just one degree (°C), wheat yields will decrease by 6% (Asseng et al. 2015). In the main European wheat-growing areas, the occurrence of adverse weather conditions might substantially increase by 2060, resulting in more frequent crop failure (Trnka et al. 2014). This could threaten global food security since Europe produces almost a third of the world’s wheat.

Moreover, the probability of multiple adverse events occurring within one season is projected to increase sharply by mid-century. Cold stress at meiosis could become more common in future years because climate change is expected to result in mild winters and warm springs, which are likely to speed up plant development prematurely, resulting in exposure of vulnerable plant tissues and organs to subsequent late season frosts. This occurred in 2007 when severe low temperatures following a period of above average temperatures caused widespread damage to agriculture in the USA (Gu et al. 2008). Models also predict an increase in frequency of heat stress at meiosis (Semenov et al. 2014). Our experiments have shown that even a relatively short period of high temperature at a critical stage of wheat development is sufficient for meiosis to be significantly affected. If current weather trends continue, it will become increasingly important to cultivate both heat-tolerant varieties and cold-tolerant varieties.

Heat tolerance is thought to be a complex trait in plants and is likely to be under the control of multiple genes (Barnabás et al. 2008), but we still know relatively little about the role of individual genes controlling temperature tolerance in wheat (Mullarkey and Jones 2000). Identification of *TaDmc1* as a candidate gene that can stabilise chromosome synapsis against extremes of temperature may allow wheat breeders to exploit this information and can provide markers that will allow them to identify and select hexaploid wheat genotypes that carry low (and high) temperature tolerance alleles at this locus.

## Electronic supplementary material

Below is the link to the electronic supplementary material.

Suppl. Fig. 1 Gene expression patterns of the three hexaploid wheat *TaDmc1* homeologs, TraesCS5A02G133000 (*TaDmc1*-*A1*) on chromosome 5A, TraesCS5B02G131900 (*TaDmc1*-*B1*) on 5B and TraesCS5D02G141200 (*TaDmc1*-*D1*) on 5D in different tissue types, based on the 876 RNASeq samples available in the wheat expression browser (http://www.wheat-expression.com). *TaDmc1* expression levels are higher in meiotically active tissues than non-meiotic tissues. In meiotic anthers, expression levels of *TaDmc1*-*D1* are higher than those of *TaDmc1*-*A1* and *TaDmc1*-*B1* (PDF 484 kb)

Suppl. Fig. 2 Multiple alignment of the coding sequences (CDs) of the three hexaploid wheat *Dmc1* homeologs: TraesCS5A02G133000 (*TaDmc1*-*A1*) on chromosome 5A, TraesCS5B02G131900 (*TaDmc1*-*B1*) on 5B and TraesCS5D02G141200 (*TaDmc1*-*D1*) on 5D. SNPs are shown in red text on a grey background (PDF 103 kb)

Suppl. Fig. 3 Multiple alignment of the DNA sequences of the three hexaploid wheat *Dmc1* homeologs: TraesCS5A02G133000 (*TaDmc1*-*A1*) on chromosome 5A, TraesCS5B02G131900 (*TaDmc1*-*B1*) on 5B and TraesCS5D02G141200 (*TaDmc1*-*D1*) on 5D (DOCX 21 kb)

Suppl. Fig. 4 Multiple sequence alignment of the promoter regions of the three hexaploid wheat *Dmc1* homeologs: TraesCS5A02G133000 (*TaDmc1*-*A1*) on chromosome 5A, TraesCS5B02G131900 (*TaDmc1*-*B1*) on 5B and TraesCS5D02G141200 (*TaDmc1*-*D1*) on 5D. The promoter regions include the 1500 nucleotides downstream of the start codon of each of the *TaDmc1* homeologs. Note the large insertion mutation (163 nt in size) at position 430 downstream of the start codon of *TaDmc1*-*B1* (PDF 165 kb)

Suppl. Fig. 5 Multiple alignment of *Dmc1* DNA sequences from *T. aestivum* and its diploid and tetraploid ancestors, *T. urartu* (AA), *Ae. tauschii* (DD) and *T. dicoccoides* (AABB); note the large insertion of 294 bp in the B-genome copy of the *T. aestivum* gene (DOCX 33 kb)

Suppl. Fig. 6 DMC1 transcripts in *T. aestivum* and its diploid and tetraploid ancestors (as predicted in Ensembl Plants) (DOCX 982 kb)

Suppl. Fig. 7 Multiple alignment of DMC1 amino-acid sequences from *T. aestivum* (Ta) and its diploid and tetraploid ancestors: the A-genome donor, *T. urartu* (Tu_A), the D-genome donor, *Ae. tauschii* (Aet_D) and the AABB genome progenitor, *T. dicoccoides* (Td_A and Td_B). *Ae tauschii* and *T. dicoccoides* have multiple transcripts of their *Dmc1* genes due to alternative splicing variants, but this alignment only includes transcripts that are most similar to those of *T. aestivum*. Numbers after the dots refer to the transcript number. Large red triangle indicates the single amino-acid substitution in TaDMC1-D1 that may confer low temperature tolerance in wheat. Note the conservation of this substitution in the ancestral D-genome of *Ae. tauschii*. Small blue triangles show the positions of three other amino-acid substitutions in TaDMC1-B1, and a small green triangle shows the position of a substitution in TaDMC1-A1 that is also conserved in the ancestral copies (RTF 101 kb)

Suppl. Table 1 The effects of three different temperature treatments on meiotic metaphase I chromosomes of individual Chinese Spring (CS) wild-type and 22-F5 (*ttmei1*) mutant plants. The mean numbers of univalents, ring and rod bivalents, trivalents, tetravalents and pentavalents were scored along with chiasma frequency scored as single or double crossovers. Standard deviation (SD) and standard error (SE) are shown, as well as the maximum (max) and minimum (min) number of chromosomes with a particular conformation (PDF 675 kb)

Suppl. Table 2 Expression values (TPM) of all genes inside the *Dmc1* deletion region in 876 samples from different tissue types (XLSX 246 kb)

Suppl. Table 3 *Dmc1* sequence identity matrices. a) Percentages of nucleotide sequence identity for the three *TaDmc1* homeologs: TraesCS5A02G133000 (*TaDmc1*-*A1*) on chromosome 5A, TraesCS5B02G131900 (*TaDmc1*-*B1*) on 5B and TraesCS5D02G141200 (*TaDmc1*-*D1*) on 5D; b) percentages of amino-acid sequence identity for DMC1 proteins in wheat (TaDMC1-A1, TaDMC1-B1 and TaDMC1-D1), barley (HvDMC1) and rice (OsDMC1A and OsDMC1B); c) percentages of nucleotide sequence identity for *Dmc1* homeologs in *T. aestivum* (*TaDmc1*) and its progenitor genomes: *T. urartu* (*TuDmc1*-*A*), *Ae. tauschii* (*AetDmc1*-*D*) and *T. dicoccoides* A genome (*TdDmc1*-*A*) and B genome (*TdDmc1*-*B*); d) percentages of amino-acid sequence identity for DMC1 proteins in *T. aestivum* (TaDMC1) and its progenitor genomes: *T. urartu* (TuDMC1-A), *Ae. tauschii* (AetDMC1-D) and *T. dicoccoides* A genome (TdDMC1-A) and B genome (TdDMC1-B). Two different splice variants each are shown for TdDMC1-A, TdDMC1-B and AetDMC1-D. Note that of all the amino-acid sequences of *T. aestivum* and its progenitors, *T. urartu* is the most divergent (XLSX 14 kb)
