# Supplementary figures and images for: Dmc1 is a candidate for temperature tolerance during wheat meiosis
Source: Theor Appl Genet. 2019 Dec 18;133(3):809–28. doi: 10.1007/s00122-019-03508-9 (PMC7021665; doi:10.1007/s00122-019-03508-9)

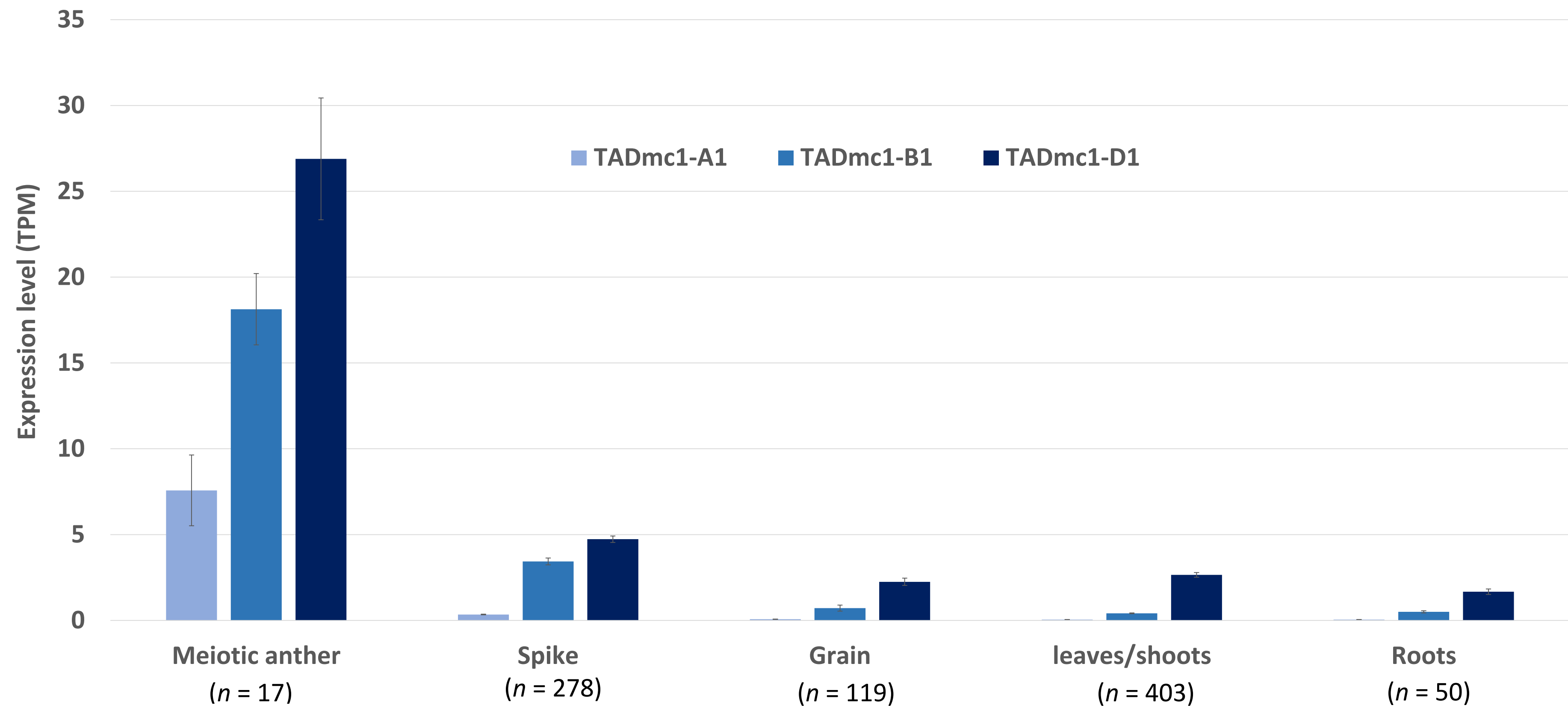

Supplement: Supplementary file 1 — Gene expression patterns of the three hexaploid wheat TaDmc1 homeologs, TraesCS5A02G133000 (TaDmc1-A1) on chromosome 5A, TraesCS5B02G131900 (TaDmc1-B1) on 5B and TraesCS5D02G141200 (TaDmc1-D1) on 5D in different tissue types, based on the 876 RNASeq samples available in the wheat expression browser (http://www.wheat-expression.com). TaDmc1 expression levels are higher in meiotically active tissues than non-meiotic tissues. In meiotic anthers, expression levels of TaDmc1-D1 are higher than those of TaDmc1-A1 and TaDmc1-B1 (PDF 484 kb) [file 122_2019_3508_MOESM1_ESM.pdf]
