# Supplementary material for: Dmc1 is a candidate for temperature tolerance during wheat meiosis
Source: Theor Appl Genet. 2019 Dec 18;133(3):809–28. doi: 10.1007/s00122-019-03508-9 (PMC7021665; doi:10.1007/s00122-019-03508-9)
Supplement: Supplementary file 2 — Multiple alignment of the coding sequences (CDs) of the three hexaploid wheat Dmc1 homeologs: TraesCS5A02G133000 (TaDmc1-A1) on chromosome 5A, TraesCS5B02G131900 (TaDmc1-B1) on 5B and TraesCS5D02G141200 (TaDmc1-D1) on 5D. SNPs are shown in red text on a grey background (PDF 103 kb) [file 122_2019_3508_MOESM2_ESM.pdf]

```
                        *        20         *        40         *        60         *        80         *       100         *       120         *       140         *
TaDmc1-A1 : ATGGCGCCGTCCAAGCAGTACGACGAGGGCGGGCAGCTCCAGCTCATGGAGGCCGACCGGGTCGAGGAGGAGGAGGAGTGCTTCGAGTCCATCGACAAGTTGATCTCGCAGGGAATAAACTCAGGAGACGTGAAGAAGCTGCAGGATGCG :  150
TaDmc1-B1 : ATGGCGCCGTCCAAGCAGTACGACGAGGGCGGGCAGCTCCAGCTCATGGAGGCCGACCGGGTCGAGGAGGAGGAGGAGTGCTTCGAGTCCATCGACAAGTTGATCTCGCAGGGAATTAACTCAGGAGACGTGAAGAAGCTGCAGGATGCG :  150
TaDmc1-D1 : ATGGCGCCGTCCAAGCAGTACGACGAGGGCGGGCAGCTCCAGCTCATGGAGGCCGACCGGGTGGAGGAGGAGGAGGAGTGCTTCGAGTCCATCGACAAGTTGATCTCGCAGGGAATAAACTCAGGAGACGTGAAGAAGCTGCAGGATGCG :  150

                   160         *       180         *       200         *       220         *       240         *       260         *       280         *       300
TaDmc1-A1 : GGGATCTACACTTGCAATGGGCTGATGATGCACACCAAGAAGAGCCTTACAGGGATTAAGGGCTTGTCTGAAGCAAAGGTTGATAAGATCTGCGAGGCTGCTGAAAAACTTCTGAGTCAGGGTTTCATGACAGGAAGTGATCTCCTTATT :  300
TaDmc1-B1 : GGGATCTACACTTGCAATGGCCTGATGATGCACACCAAGAAGAGCCTTACAGGGATTAAGGGCTTGTCTGAAGCAAAGGTTGATAAGATCTGCGAGGCTGCTGAAAAACTTCTGAGTCAGGGTTTCATGACAGGAAGTGATCTCCTTATT :  300
TaDmc1-D1 : GGGATCTACACTTGCAATGGGCTGATGATGCACACCAAGAAGAGCCTTACAGGGATTAAGGGCTTGTCTGAAGCGAAGGTTGATAAGATCTGTGAGGCTGCTGAAAAACTTCTGAGTCAGGGTTTCATGACAGGAAGTGATCTCCTTATT :  300

                        *       320         *       340         *       360         *       380         *       400         *       420         *       440         *
TaDmc1-A1 : AAGCGAAAGTCTGTTGTCCGGATTACCACTGGGAGCCAAGCGCTTGATGAGCTGCTTGGAGGAGGGATTGAAACACTCTGTATCACAGAGGCATTTGGAGAGTTCCGGTCAGGGAAGACCCAGTTGGCTCATACTCTTTGTGTCTCCACT :  450
TaDmc1-B1 : AAGCGAAAGTCTGTTGTCCGGATTACCACTGGGAGCCAAGCGCTTGATGAGCTGCTTGGAGGAGGGATTGAAACACTCTGTATCACAGAGGCATTTGGAGAGTTCCGGTCAGGGAAGACCCAGTTGGCTCATACTCTTTGTGTCTCCACT :  450
TaDmc1-D1 : AAGCGAAAGTCTGTTGTCCGGATTACCACTGGGAGCCAAACGCTTGATGAGCTGCTTGGAGGAGGGATTGAAACACTCTGTATCACAGAGGCATTTGGAGAGTTCCGGTCAGGGAAGACCCAGTTGGCTCATACTCTTTGTGTCTCCACT :  450

                   460         *       480         *       500         *       520         *       540         *       560         *       580         *       600
TaDmc1-A1 : CAGCTTCCACTCCACATGCATGGTGGGAACGGGAAGGTTGCCTACATTGACACTGAGGGAACATTCCGGCCTGAACGCATTGTGCCAATTGCTGAGAGATTTGGGATGGATGCCAATGCTGTTCTTGACAATATCATATACGCTCGCGCA :  600
TaDmc1-B1 : CAGCTTCCACTCCATATGCATGGTGGGAACGGGAAGGTTGCCTACATTGGCACTGAGGGAACGTTCCGGCCTGAACGCATTGTGCCAATTGCTGAGAGATTTGGGATGGATGCCAATGCTGTTCTTGACAATATCATATACGCTCGTGCA :  600
TaDmc1-D1 : CAGCTTCCACTCCACATGCATGGTGGGAACGGGAAGGTTGCCTACATTGACACTGAGGGAACATTCCGGCCTGAACGCATTGTGCCAATTGCTGAGAGATTTGGGATGGATGCCAATGCTGTTCTTGACAATATCATATATGCTCGTGCC :  600

                        *       620         *       640         *       660         *       680         *       700         *       720         *       740         *
TaDmc1-A1 : TACACCTATGAGCACCAGTACAACTTACTCCTGGGCCTTGCTGCCAAGATGGCCGAAGAGCCTTTCAGGCTTCTGATCGTGGATTCTGTGATTGCGCTATTCCGTGTTGATTTCAGTGGTAGGGGTGAACTTGCAGAGCGTCAGCAAAAA :  750
TaDmc1-B1 : TACACCTATGAGCACCAGTACAACTTACTCCTGGGCCTTGTTGCCAAGATGGCTGAAGAGCCTTTCAGGCTTCTGATCGTGGATTCTGTGATTGCGCTATTCCGTGTTGATTTCAGTGGCAGGGGTGAACTTGCAGAGCGTCAGCAAAAA :  750
TaDmc1-D1 : TACACCTATGAGCACCAGTACAACTTACTCCTGGGCCTTGCTGCCAAGATGGCTGAAGAGCCTTTCAGGCTTCTGATCGTGGATTCTGTGATTGCGCTGTTCCGTGTTGATTTCAGTGGTAGGGGTGAACTTGCAGAGCGTCAGCAAAAG :  750

                   760         *       780         *       800         *       820         *       840         *       860         *       880         *       900
TaDmc1-A1 : CTGGCACAAATGCTGTCCCGCCTTACAAAGATTGCTGAGGAGTTCAATGTTGCAGTGTACATCACCAACCAAGTGATTGCGGACCCAGGTGGTGGTATGTTCATCACTGACCCCAAAAAGCCGGCAGGAGGCCACGTGCTGGCGCATGCA :  900
TaDmc1-B1 : CTGGCACAAATGCTTTCCCGCCTTACAAAGATTGCTGAGGAGTTCAATGTTGCAGTGTACATCACCAACCAAGTGATTGCGGACCCAGGTGGTGGTATGTTCATCACTGACCCCAAAAAGCCGGCGGGAGGCCACGTGCTGGCGCATGCA :  900
TaDmc1-D1 : CTGGCACAAATGCTGTCCCGCCTTACAAAGATTGCTGAGGAGTTCAATGTTGCAGTGTACATCACCAACCAAGTGATTGCGGACCCAGGTGGTGGTATGTTCATCACTGACCCCAAAAAGCCGGCGGGAGGCCACGTGCTGGCGCATGCA :  900

                        *       920         *       940         *       960         *       980         *      1000         *      1020         *
TaDmc1-A1 : GCCACCATCCGGTTGATGCTGAGGAAAAGCAAAGGCGAGCAGCGTGTCTGCAAGATCTTTGACGCCCCTAACCTTCCCGAGGGAGAAGCTGTTTTCCAGATTACAACAGGCGGATTGATGGATGTGAAAGACTGA : 1035
TaDmc1-B1 : GCCACCATCCGGTTGATGCTGAGGAAAGGCAAAGGCGAGCAGCGTATCTGCAAGATCTTTGACGCCCCTAACCTTCCCGAGGGAGAAGCTGTTTTCCAGATTACAACAGGTGGATTGATGGATGTGAAAGACTGA : 1035
TaDmc1-D1 : GCCACCATCCGGTTGATGCTGAGGAAAGGCAAAGGCGAGCAGCGTGTCTGCAAGATCTTTGACGCCCCTAACCTTCCCGAGGGAGAAGCTGTTTTCCAGATCACAACAGGCGGATTGATGGATGTGAAAGACTGA : 1035
```
