# Supplementary material for: Dmc1 is a candidate for temperature tolerance during wheat meiosis
Source: Theor Appl Genet. 2019 Dec 18;133(3):809–28. doi: 10.1007/s00122-019-03508-9 (PMC7021665; doi:10.1007/s00122-019-03508-9)
Supplement: Supplementary file 3 — Multiple alignment of the DNA sequences of the three hexaploid wheat Dmc1 homeologs: TraesCS5A02G133000 (TaDmc1-A1) on chromosome 5A, TraesCS5B02G131900 (TaDmc1-B1) on 5B and TraesCS5D02G141200 (TaDmc1-D1) on 5D (DOCX 21 kb) [file 122_2019_3508_MOESM3_ESM.docx]

* 20 * 40 * 60 * 80 * 100 * 120 * 140 *
***TaDmc1-A1*** : ATGGCGCCGTCCAAGCAGTACGACGAGGGCGGGCAGCTCCAGCTCATGGAGGCCGACCGGGTCGAGGAGGAGGAGGAGTGCTTCGAGTCCATCGACAAGTGTACGTTCGCCGCCTCCTACCCCTCTCCTCTCGAAACCCGCCCGCTGCCC : 150
***TaDmc1-B1*** : ATGGCGCCGTCCAAGCAGTACGACGAGGGCGGGCAGCTCCAGCTCATGGAGGCCGACCGGGTCGAGGAGGAGGAGGAGTGCTTCGAGTCCATCGACAAGTGTATGTTCGCCGCCTCCAACTCCTCTCCTCTCGAAACCCTCCCGCCGCCC : 150
***TaDmc1-D1*** : ATGGCGCCGTCCAAGCAGTACGACGAGGGCGGGCAGCTCCAGCTCATGGAGGCCGACCGGGTGGAGGAGGAGGAGGAGTGCTTCGAGTCCATCGACAAGTGTACGTTCGCCGCCTCCAACCCCTCTCCTCTCCAAACCCTCCCGCCGCCC : 150

 160 * 180 * 200 * 220 * 240 * 260 * 280 * 300
***TaDmc1-A1*** : CGTCTCCGGTGCTGCATTTACTTGCTTGTTCGTGTGCCTGCGTCGCGCGTGTGTCGGCATGTGGGGGTTAGGCCTGCTCACCGTTGCGTTCCGGGTGCTTCCGCCTCTAAGTTCGCGCGTTTCGGTCGCAATTTCGTGCTGTTTGGAGAT : 300
***TaDmc1-B1*** : CGTCTCCGGTGCTGCATTTACTTGCTTGTCCGTGTGCCTGCGTCGCGCGCGTGTCGGCGCGTGGGGGTTAGGCCTGCTCACCGTTGCGTTCCGGGTGCTTCTGCCTCTAAGTTCGCGCGTTTCGGTCACAATTTCGTGCTGTTTGGAGAT : 300
***TaDmc1-D1*** : CGTCTCCGGTGCTGCATTTACTTGCTTGTTCGTGTGCCTGCGTCGCGCGCGTGTCGGCGCGTGGGGGTTAGGCCTGCTCACCGTTGCGTCCCGGGTGCTTCCGCCTCTAAGTTCGCGCGTTTCGGTCGCAATTTCGTGCTGTTTGGAGAT : 300

 * 320 * 340 * 360 * 380 * 400 * 420 * 440 *
***TaDmc1-A1*** : GGGTTTGGTGCGGATTTCGCTTAGCCTCCACATTTGGTTGGTTTTTGGTGCGTGCAGCGCGTGGTCGCGTGTGCTCGTGTTGCGTTTGTTTTGATTTTCTCCCCTATTTCGTGCAGTCTGGGGATTAACTAGCGCCCTT-GGTAGGCTAA : 449
***TaDmc1-B1*** : GGATTTGGTGCGGATTTCGCTTAGCCTCCACATTTGGTTGGTTTTTGGTGCGTGCAGCGCGTGGTCG--TGTGCTCGTGTTGCGTTGGTTTTGATTTTCTCCCCTATTTCGTGCAATCTGGGGATTAACTAGCGCCCTTTGGTAGCCTAA : 448
***TaDmc1-D1*** : GGATTTGGTGCGGATTTCGCTTAGCCTCCACATTTGGTTGGTTTTTGGTGCGTGCAGCGCGTGGTCG--TGTGCTCCTGTTGCGTTGGTTTTGATTTTCTCCCCTATGTCGTGCAATCTGGGGATTAACTAGCGCCCTTTGGTAGCCTAA : 448

 460 * 480 * 500 * 520 * 540 * 560 * 580 * 600
***TaDmc1-A1*** : TTTCGTGCAGCTTTGCTGATTCCCCCCACTTGATTTCTCTTTACCGGTTAGTTTGTGCTAATCGCTGTGTGGTGCCGTTTTTTCTCCACGGATTAGTCTGAACTTAGCCCCAATTTTGAGCTGCCATGCGTTTAATCCGCCGTAGCGATC : 599
***TaDmc1-B1*** : TTTCGTGCGGCTTTTCTAATTCCCCCCACTTGATTTCTCTTTACCGGCTAGTTTGTGCTAATCGCTGTGTGGTGCCGTTTTTTCTCCACGGATTAGTCTGAACTTAGCCCCAATTTTGAGCTGCCATGCCTTTAATCCGCCGTATCGATC : 598
***TaDmc1-D1*** : TTTCGTGCAGCTTTGTTGATGCCCCCCACTTGATTTCTCTTTACCGGCTAGTTTGTGCTAATCGCTGTGTGGTGCCGTTTTTTCTCCACGAATTAGTCTGAATTTAGCCCCAATTTTGAGCTGCCATGCGTTTAATCTGCCGTAGCGATC : 598

 * 620 * 640 * 660 * 680 * 700 * 720 * 740 *
***TaDmc1-A1*** : ACTGTCTGTAGAATCCTTTCGAATTCGGCACTAGATACAGTTCAATCGCGTGGGTTTTCTGTGGAAACTTGGACCAATTACGGACGCGTTTTCGTCCACTTCGTGGCGTAGGGTTT-AAGCCTGTCTTCCTCATCTGTTCCACTTTTTTT : 748
***TaDmc1-B1*** : ACTGGCTGTAGAATCCTGTCGAATTCGAAACTAGATACAGTTCAATCGCGTGGGTTTTCTGTGGAAGCTTGGACCAATTACGGACGCGTTTTCGTGCACTTCGAGGCGTAGGGTTTTAAGCCTGTCGTCCTCATCTGTTCCACTTTTTTT : 748
***TaDmc1-D1*** : ACTGGCTGTAGAATCCTTTCGAATTCGAAACTAGATACAGTTCAATCGCGTGGGTTTTCTGTGGAAACTTGGACCAATTACGGACGCGTTTTCCTGCACTTCGAGGCGTAGGGTTTTAAGCCTGTCTTCCTTATCTGTTCCACTTTTTTT : 748

 760 * 780 * 800 * 820 * 840 * 860 * 880 * 900
***TaDmc1-A1*** : TTTCTGAATGTTCACCTGTTCTGTTCCATAATTACGGGTAGCTTCCGCGAGTTTAGCAAGTTTACTTTAAACATTTGTCTAGATGTGATCCGCGCTTGTGTTTTGTTTGCGTGATCCAATTGGTTCGTCGTGCACTTTGTTCATTGCCAT : 898
***TaDmc1-B1*** : TTTCTGAATGTTCACCTGTTCTGTTCCATAATCACGGGTAGCTTCCGCGAGTTTAGCAAGTTTACTTTAAACATTTGCCTAGATGTGATCCGCGCTTGTGTTTTGTTTGCGTGATCCAATTGGTTCGTCGTGCACTTTGTTCATTGCCAT : 898
***TaDmc1-D1*** : TT-CTGAATGTTCACCTGTTATGTTCCATAATTACGGGTAGCTTCCGCGAGTTTAGCAAGTTTACTTTAAACATTTGCCTAGATGTGATCCGCGCTTGTGTTTTGTTTGCGTGATCCAATTGGTTCGTCGTGCACTTTGTTCATTGCCAT : 897

 * 920 * 940 * 960 * 980 * 1000 * 1020 * 1040 *
***TaDmc1-A1*** : GAATTTGAATCCTAAGCCGTAACCTTCCTTTTAACGCTGTAACTGATCACTCGTCCAAACAGTGATCTCGCAGGGAATAAACTCAGGAGACGTGAAGAAGCTGCAGGATGCGGGGATCTACACTTGCAATGGGCTGATGATGCACACCAA : 1048
***TaDmc1-B1*** : GAATTTGAATCCTAAGCCGT------------AACGCTGTAACTAATCACTCGTCCAAACAGTGATCTCGCAGGGAATTAACTCAGGAGACGTGAAGAAGCTGCAGGATGCGGGGATCTACACTTGCAATGGCCTGATGATGCACACCAA : 1036
***TaDmc1-D1*** : GAATTTGAATCCTAAGCCGTAACATTCCTTGTAACGCTGTAACTAATCACTCGTCCAAACAGTGATCTCGCAGGGAATAAACTCAGGAGACGTGAAGAAGCTGCAGGATGCGGGGATCTACACTTGCAATGGGCTGATGATGCACACCAA : 1047

 1060 * 1080 * 1100 * 1120 * 1140 * 1160 * 1180 * 1200
***TaDmc1-A1*** : GAAGGTCCCAATCCCGTCAGAGCATAAATCTGCACGCATTCTCCCTAAAATTTGTGGTTGCATACTGAAGCTGATTTCTGGTGCACGACACATGACTGTTATTAGTTGCTTTATGTATTCAAGTTTCAACACATGATTGATGTTGTTTCA : 1198
***TaDmc1-B1*** : GAAGGTCAAACTCCCTTCAGAGCCTAAATCTGCATGCATTCTCCCTAAAATTTGTGGTTGCATACTGAAGTTGATTTCTGGTGCACGACACATGACTGTTATTAGTTGTTTTATGTATTCAAGTTTCAACACATGATTGATGTTGTTTCA : 1186
***TaDmc1-D1*** : GAAGGTCAAAATCCCTTCAGAGCCTAAATCTGCACGCATTCTCCCTAAAATTTGTGGTTGCATGCTGAAGTTGATTTCTGGTGCACGACACATGACTGTTATTAGTAGCTTTATGTATTCAAGTTTCAACACATGATTGATGTTGTTTCA : 1197

 * 1220 * 1240 * 1260 * 1280 * 1300 * 1320 * 1340 *
***TaDmc1-A1*** : GTACCAATGTATGGATTCATCTTGCAGAGCCTTACAGGGATTAAGGGCTTGTCTGAAGCAAAGGTTGATAAGATCTGCGAGGCTGCTGAAAAACTTCTGGTATGATTGTTATCATCTTTGCATTGATTCTGGTTTACAACTTCTGTGCCA : 1348
***TaDmc1-B1*** : GTATCAATGTATGGATTCATCTTGCAGAGCCTTACAGGGATTAAGGGCTTGTCTGAAGCAAAGGTTGATAAGATCTGCGAGGCTGCTGAAAAACTTCTGGTATGATTGTTATCATCTTTGCATTGATTCTGGTTTAGAACTTCTGTGCCA : 1336
***TaDmc1-D1*** : GTATCAATGTACGGATTCATCTTGCAGAGCCTTACAGGGATTAAGGGCTTGTCTGAAGCGAAGGTTGATAAGATCTGTGAGGCTGCTGAAAAACTTCTGGTATGATTGTTATCATCTTTGCATTGATTCTGGTTTAGAACTTCTGTGCCA : 1347

 1360 * 1380 * 1400 * 1420 * 1440 * 1460 * 1480 * 1500
***TaDmc1-A1*** : TATTATTCTCTTTGCATTGATTCTGGTTTACAACTTCTGTGCCATATTATTCTCTTTGCATTGATTCTGGTTTACAACTTCTGTGCCATCTGTGCTATGCGATCGAGTCTTCTGAAATTTGTATTCTATTTGATTTTCAGAGTCAGGGTT : 1498
***TaDmc1-B1*** : TA--------------------------------------------TTATTCTCTTTGCATTGATTCTGGTTTACAACTTCTGTGACATCTGTGCTATACGATCGAGTCTTCTGAAATTTGTATTCTATTTGATTTTCAGAGTCAGGGTT : 1442
***TaDmc1-D1*** : TA--------------------------------------------TTATTCTCTTTGCATTGATTCTGGTTTACAACCTCTGTGCCATCTGTGCTATGCGATAGAATCTTCTGAAGTTTGTGTACTATTTGATTTTCAGAGTCAGGGTT : 1453

 * 1520 * 1540 * 1560 * 1580 * 1600 * 1620 * 1640 *
***TaDmc1-A1*** : TCATGACAGGAAGTGATCTCCTTATTAAGGTAAGGTTTAGGGAGCTAAGCTACTGATGAAGGACGACCATACAGTTTAGTGCTGTTTTTGCAGTAGTGCTACACCGCTACTATGCTAAACTTACTGTAA----TAGTTTGTTTTTGGAAT : 1644
***TaDmc1-B1*** : TCATGACAGGAAGTGATCTCCTTATTAAGGTAAGGTTTAGGGAGCTAAGCTACTGATGAAGGACGATCATACAGTTTAGTGCTGTTTTTGCAGTAGTGCTACACTGCTACTATGCTAAACTTACTGTTA----TAGTTTGTTTTTGGAAT : 1588
***TaDmc1-D1*** : TCATGACAGGAAGTGATCTCCTTATTAAGGTGAGGTTTAGG-AGCTAAGCTACTGATTAAGGACGATCATACAGTTTAGTACTGTTTTGGCCATAGTG--------CTACTATGCTAAACTTACTGTAAATAATAGTTTGTTTTTGGAAT : 1594

 1660 * 1680 * 1700 * 1720 * 1740 * 1760 * 1780 * 1800
***TaDmc1-A1*** : CTGCTTGAGTGCTTTCATTACTCCACCTGTGTACTGCCCAGTTGTTTGATTTACTTTTTCGTATATTGAAGCGAAAGTCTGTTGTCCGGATTACCACTGGGAGCCAAGCGCTTGATGAGCTGCTTGGAGGTAACATATGTCGCCCTTGAT : 1794
***TaDmc1-B1*** : CTGCTTGAGTGCTTTCATTACTCCACCTGTGTACTGCCCGGTTGTTTGATTTACTTTTCCGTATATTGAAGCGAAAGTCTGTTGTCCGGATTACCACTGGGAGCCAAGCGCTTGATGAGCTGCTTGGAGGTAACATATGTCGTTCTTGAT : 1738
***TaDMC1-D1*** : CTGCTTGAGTGCTTTCATTACTCCACCTGTGTACTGCCCGGTTGTTTGATTTACTTTTCCGTATATTGAAGCGAAAGTCTGTTGTCCGGATTACCACTGGGAGCCAAACGCTTGATGAGCTGCTTGGAGGTAACATATGTCATCCTTGAT : 1744

 * 1820 * 1840 * 1860 * 1880 * 1900 * 1920 * 1940 *
***TaDmc1-A1*** : TCTGTTCTGATTATTTCTGATGTTATGCTCTAACCCATTCACATATTTCCATAATTTGAAGGAGGGATTGAAACACTCTGTATCACAGAGGCATTTGGAGAGTTCCGGTCAGTAAATGTTCCAGTTACCATTTTCTTCTGGATTT----- : 1939
***TaDmc1-B1*** : TCTGTTCTGATTATTTCTGATGTTATGCTCTAACCTATTCACATATTTCCATAATTTGAAGGAGGGATTGAAACACTCTGTATCACAGAGGCATTTGGAGAGTTCCGGTCAGTAAATGTTCCGGTTACCATTTTCTTCTGGATTTTT--- : 1885
***TaDmc1-D1*** : TCTGTTCTGATTATTCCTGATGTTATGCTCTAACCTATTCAAATATTTGCGTAATTTGAAGGAGGGATTGAAACACTCTGTATCACAGAGGCATTTGGAGAGTTCCGGTGAGTAAATGTTCCCGTTACCATTTTCTTCTTGGTTTCTTCT : 1894

 1960 * 1980 * 2000 * 2020 * 2040 * 2060 * 2080 * 2100
***TaDmc1-A1*** : TTTTTTGCAGGGAGCGCCTATTAGTTTTATCTCTGTTATGTATGATTTGTGCCATATTTCGCAGTAGATGTAGCAATTTTTAGTCCTAGTAATTTTTTAGT---TGGT----AAACTCTGATAAGCTGCTTTCACCTCCTTTTCTGCTGT : 2082
***TaDmc1-B1*** : TTTTTTGCAGGGGGCGCCTATTAGTTGTATCTCTGTTATGTATGATTTGTGCCATATTTGGCAGTAGATGTAACAATTTTTAGTCCTAGTAATTTTTTAGT---TGGT---AAAACTCTGATAAGCTTCTTTCACCTCCTTTTCTGCTGT : 2029
***TaDmc1-D1*** : TTTTTTGCAGGGACCGCCTATTAGTTGTAGCTCTGTTATGTATGATTTGTGCCATATTTGGCAGTAG------------CTAGTCCTAGTAATTTTTTAGTACATGGTGCTAAAACTCTCATAAGCTTCTTTCACCTCCTTTTCTACTGT : 2032

 * 2120 * 2140 * 2160 * 2180 * 2200 * 2220 * 2240 *
***TaDmc1-A1*** : CTAGTCTTCCAGAGGTATTGATGATGGTGCATAT-------------------------------------------------------------------------------------------------------------------- : 2116
***TaDmc1-B1*** : CTAGCCTTCCAGAGGTATTGATGATAGTGCATATAACAACTCCTAAGCATGGTTGCTGTGATGCTAACATTGAATACTGGATACCTGTTACATGCCAAAATTCAAACATATCTACATCAATATTATTATTCACTATTTCTCTGATGCAAC : 2179
***TaDmc1-D1*** : CTAGTCTTCCAGAGGTATTGATGGTAGTGCATATAAGAATTCGAAAGCATGGTTGCTGTGATGCTAACATTGAATACTGGATACATGTTGCATGCCAAAACTCAAACAAATCTACATCAATATTACTAT-CACTATTTCCCTGATGCAAC : 2181

 2260 * 2280 * 2300 * 2320 * 2340 * 2360 * 2380 * 2400
***TaDmc1-A1*** : ------------------------------------------------------------------------------------TCAGTTCCAACTTTCTAGCGCTAAAATCCATATTATTTACTTTGTCTTGCAGGTCAGGGAAGACCC : 2182
***TaDmc1-B1*** : ACACACATGCTCTTATTATTATTCAATGAGCACGGTTGTAATGTTGACTTCCAAAGCACATTTGTTACTCAAACATTTCTCAATTCAGTTCCAACTTTCTAGCGCTAAAATCCATATTATTTACTTTGTTTTGCAGGTCAGGGAAGACCC : 2329
***TaDmc1-D1*** : ACGCACCTGCTCTTATTATT-------GAGCACAGTTGTAATGTTGATTTCCAAAGCACATTTGTTACTCAACCATTTC-----TCAATTCCAACTTTCTAGCGTTAAAATCCATATTATTTACTTTGTCTTGCAGGTCAGGGAAGACCC : 2319

 * 2420 * 2440 * 2460 * 2480 * 2500 * 2520 * 2540 *
***TaDmc1-A1*** : AGTTGGCTCATACTCTTTGTGTCTCCACTCAGGTCCATTTCCTGCCTTGTATATTCTCGGTGAAACCTCACTACATCAGAATCCATGAATAACTCTGCTTGTTTAATCAAA--------------------------------------- : 2293
***TaDmc1-B1*** : AGTTGGCTCATACTCTTTGTGTCTCCACTCAGGTCCATTTCCTGCCTTGTAT-TTCTCGGTGAAACCTCACTACATCAGAATCCATGAATAACTCTGCTTGTTTAATCAAAGAGTGAAGTGCACCCTAGGTCCTCGAACTATTTTGAAGG : 2478
***TaDmc1-D1*** : AGTTGGCTCATACTCTTTGTGTCTCCACTCAGGTCCATTTCCTGCCTTGTATTTTATCGATGAAACCTCACTAGAACAGAATCCATGAATAACTCTCCATGTTTAATCAAA--------------------------------------- : 2430

 2560 * 2580 * 2600 * 2620 * 2640 * 2660 * 2680 * 2700
***TaDmc1-A1*** : ------------------------------------------------------------------------------------------------------------------------------------------------------ : -
***TaDmc1-B1*** : TGTCATATAGGTCCTCGAATTATGAAAAGTGTCATCCAGGTCCTCAAAAATCCTTGAAGTGCAATAAGTGTGTACGTATGTGACACATTTGAAATAGTTTAAGGACCTACATGACACCGCTAAAATAGTTCGAGGACTTGGATGACACGC : 2628
***TaDmc1-D1*** : ------------------------------------------------------------------------------------------------------------------------------------------------------ : -


 * 2720 * 2740 * 2760 * 2780 * 2800 * 2820 * 2840 *
***TaDmc1-A1*** : ---------------------------------------------------------------------------------------------------------TGTATAACAGCTTCCACTCCACATGCATGGTGGGAACGGGAAGGT : 2338
***TaDmc1-B1*** : ATATTGCACTTTGAGGACCTGAATGACCCTTTTCATAGTTCGAGGACCTACGTAACACCTTTTAAATAGTTTGAGGACCTTTGATGCACTTCACTCTTAATCAAATGTATAACAGCTTCCACTCCATATGCATGGTGGGAACGGGAAGGT : 2778
***TaDmc1-D1*** : ---------------------------------------------------------------------------------------------------------TGTATAACAGCTTCCACTCCACATGCATGGTGGGAACGGGAAGGT : 2475
 TGTATAACAGCTTCCACTCCAcATGCATGGTGGGAACGGGAAGGT

 2860 * 2880 * 2900 * 2920 * 2940 * 2960 * 2980 * 3000
***TaDmc1-A1*** : TGCCTACATTGACACTGAGGGAACATTGTATCCTTTGAATTCCTTAGTAATACCTATAGTCGATTTGTTCAATGAATTCATTTACATTCCTGTGCTTATCGAAACTATTCCCTTAAAGGAGTTATCAGCCGGCCTGAACGCATTGTGCCA : 2488
***TaDmc1-B1*** : TGCCTACATTGGCACTGAGGGAACGTTGTATCCTTTGAATTCCTTAGTAATACCTAT-----------TTAATGAATTCATTTACATTCCTGTATTTATCGAAACTGTTCCCTTAACGGAGTTATCAGCCGGCCTGAACGCATTGTGCCA : 2917
***TaDmc1-D1*** : TGCCTACATTGACACTGAGGGAACATTGTATCCTTTGAATTCCTTAGTAATACCTATAGCTGATTTGTTCGATGAATTCATTTACATTCCTGTATTTCTCGAAACTGTTCCCTTAACGGAGTTATCAGCCGGCCTGAACGCATTGTGCCA : 2625

 * 3020 * 3040 * 3060 * 3080 * 3100 * 3120 * 3140 *
***TaDmc1-A1*** : ATTGCTGAGAGATTTGGGATGGATGCCAATGCTGTTCTTGACAATGTATGGCTCCTTTTACATCTCTCTTA--ACCCATTTAAGGAAGAATAGATCAAGATCTTTGTTTAATTCGTGATCTTTCTGTTTTAGATCATATACGCTCGCGCA : 2636
***TaDmc1-B1*** : ATTGCTGAGAGATTTGGGATGGATGCCAATGCTGTTCTTGACAATGCATGGCTCCTTTTACATCTCTCTTA--ACCCATTTAAGGAAGAATAGATAAAGATCTTTGTTTAATTCGTGATCTTTCTGTTTTAGATCATATACGCTCGTGCA : 3065
***TaDmc1-D1*** : ATTGCTGAGAGATTTGGGATGGATGCCAATGCTGTTCTTGACAATGTATGGGTCCTTTTACATCTCTCTTATAACCCATTTAAGGAAGAATAGATCGAGATCTTTGTTTAATTTGTGATCTTTCTGTTTTAGATCATATATGCTCGTGCC : 2775

 3160 * 3180 * 3200 * 3220 * 3240 * 3260 * 3280 * 3300
***TaDmc1-A1*** : TACACCTATGAGCACCAGTACAACTTACTCCTGGGCCTTGCTGCCAAGATGGCCGAAGAGCCTTTCAGGCTTCTGGTACGCATGACTTTGCTGACATGTACTATCAAACTTACAAGTTGATAGATCTCAACTGTGCTCATGTGATCTTTG : 2786
***TaDmc1-B1*** : TACACCTATGAGCACCAGTACAACTTACTCCTGGGCCTTGTTGCCAAGATGGCTGAAGAGCCTTTCAGGCTTCTGGTACGCATGACTTTGCTGACATGTAGTGTTAAACTTACAAGTTGATAGATCTCAACTGTGCTCATGTAATCTTTG : 3215
***TaDmc1-D1*** : TACACCTATGAGCACCAGTACAACTTACTCCTGGGCCTTGCTGCCAAGATGGCTGAAGAGCCTTTCAGGCTTCTGGTACGCATGACTTTGCTGCCATGT------AAATTTACAAGTTGATAGATCTCAACTGTGCTCATGTGTTCTTTG : 2919

 * 3320 * 3340 * 3360 * 3380 * 3400 * 3420 * 3440 *
***TaDmc1-A1*** : TTTGGCTTGGAAATGATAGATCGTGGATTCTGTGATTGCGCTATTCCGTGTTGATTTCAGTGGTAGGGGTGAACTTGCAGAGCGTCAGGTATTCTACTGTAACTAGCTAACTATGTGAAAAAATCAAGCAACTCATGATGTAGTCGAATG : 2936
***TaDmc1-B1*** : TTTGGCTTGGAAATGATAGATCGTGGATTCTGTGATTGCGCTATTCCGTGTTGATTTCAGTGGCAGGGGTGAACTTGCAGAGCGTCAGGTATTCTACTGTAACTAGCTAACTATGTGAAAAAATCAAGCAACTCATGATGTAGTCGAATG : 3365
***TaDmc1-D1*** : TTTGACTTGGAAATGATAGATCGTGGATTCTGTGATTGCGCTGTTCCGTGTTGATTTCAGTGGTAGGGGTGAACTTGCAGAGCGTCAGGTATTCTACTGTAACTAGCTAACTACATGAAAAAATCAAGCAACTCATGAAGTAGTCGAATG : 3069

 3460 * 3480 * 3500 * 3520 * 3540 * 3560 * 3580 * 3600
***TaDmc1-A1*** : CT---TGCATTTTATACACTTGCTCTAAGTGATGTGCTCTGGAACTGCAGCAAAAACTGGCACAAATGCTGTCCCGCCTTACAAAGATTGCTGAGGAGTTCAATGTTGCAGTGTACATCACCAACCAAGGTGTGCTTT-CCAATCTATCC : 3082
***TaDmc1-B1*** : CT---TGCATTTTATACACTTGCTCTAAGTGATGTGCTCTGGAACTGCAGCAAAAACTGGCACAAATGCTTTCCCGCCTTACAAAGATTGCTGAGGAGTTCAATGTTGCAGTGTACATCACCAACCAAGGTGTGCTTTTCCAATCTATCC : 3512
***TaDmc1-D1*** : CTGCTTGCATTTTATACACTTGCTTTAAGTGATGTGCTCTGGAACTGCAGCAAAAGCTGGCACAAATGCTGTCCCGCCTTACAAAGATTGCTGAGGAGTTCAATGTTGCAGTGTACATCACCAACCAAGGTGTGCTTT-CCAATCTATCC : 3218

 * 3620 * 3640 * 3660 * 3680 * 3700 * 3720 * 3740 *
***TaDmc1-A1*** : TGTCTGTTCCAAGAAAGAGCTCCTTATATTCGTGGATCTCAAATCATATAAGTTCTTTCCTTGTTCCAGTGATTGCGGACCCAGGTGGTGGTATGTTCATCACTGACCCCAAAAAGCCGGCAGGAGGCCACGTGCTGGCGCATGCAGCCA : 3232
***TaDmc1-B1*** : TGTCTGTTCCAAGAAAGAGCTCCTTATATTCGTGGATCTCAAATCATACAAGTTCTTTCCTTGTTCCAGTGATTGCGGACCCAGGTGGTGGTATGTTCATCACTGACCCCAAAAAGCCGGCGGGAGGCCACGTGCTGGCGCATGCAGCCA : 3662
***TaDmc1-D1*** : TGTCTGTTCCAAGAAAGAGCTCCTTATATTCGTGGATCTCAAATCATATAAGTTCTTTCCTTGTTCCAGTGATTGCGGACCCAGGTGGTGGTATGTTCATCACTGACCCCAAAAAGCCGGCGGGAGGCCACGTGCTGGCGCATGCAGCCA : 3368

3760 * 3780 * 3800 * 3820 * 3840 * 3860 * 3880 * 3900
***TaDmc1-A1*** : CCATCCGGTTGATGCTGAGGAAAAGCAAAGGCGAGCAGCGTGTCTGCAAGATCTTTGACGCCCCTAACCTTCCCGAGGGAGAAGCTATATCCTTTTGCT---TACTA------CTTGTTTACTGCTTGTGC--TATTCATTGCTTTAATT : 3371
***TaDmc1-B1*** : CCATCCGGTTGATGCTGAGGAAAGGCAAAGGCGAGCAGCGTATCTGCAAGATCTTTGACGCCCCTAACCTTCCCGAGGGAGAAGCTATATCCTTTTGCTCTATACTAGGAGTACTTGTTTACTGCTTGTGC--TATTCATTCCATTAATT : 3810
***TaDmc1-D1*** : CCATCCGGTTGATGCTGAGGAAAGGCAAAGGCGAGCAGCGTGTCTGCAAGATCTTTGACGCCCCTAACCTTCCCGAGGGAGAAGCTATATCCTTTTGCTCTATACTA------CTTGTTTACTGCTTGTGCGCTATTCATTGCTTTAATT : 3512

 * 3920 * 3940 * 3960 * 3980 * 4000 * 4020 * 4040 *
***TaDmc1-A1*** : TGTTTGGTTGCTGAACTCTTGATAGGATTGTTTTGCTCAGACTTGCACAGC----TTTATGATTTAGTTCTTAGTTGTTAACCTCCTTTGCTGAAGCTTCACGTAGCACAAATTAGCAATGATCAGATGTCGAGAAATTGTTTCCCTTGA : 3517
***TaDmc1-B1*** : TGTTTGGTTGCTGAACTCTTGATAGGATTGTTTTGCTCAGACTTGCGCAGCAAAGTTTATGATTTAGTTCT-----------------------------------------------------AGTTGTTGAGAAATTGTTTCGCTTGA : 3907
***TaDmc1-D1*** : TGTTTGGTTGTTGAACTCTTGATAGGATTGTTTTGCTCAGACTTGCACAGCAAAGTTTATGATTTAGTTCT-AGTTGTTAACCTCCTTTACTGAAGCTTCACGTAGCACAAATTAGCGACGATCAGATGTCGAGAAATTGTTTCCCTTGA : 3661

 4060 * 4080 * 4100
***TaDmc1-A1*** : CCACCAACACGTTTTCCAGATTACAACAGGCGGATTGATGGATGTGAAAGACTGA : 3572
***TaDmc1-B1*** : CCACCAACACGTTTTCCAGATTACAACAGGTGGATTGATGGATGTGAAAGACTGA : 3962
***TaDmc1-D1*** : CCACCAACACGTTTTCCAGATCACAACAGGCGGATTGATGGATGTGAAAGACTGA : 3716
