# Supplementary material for: Dmc1 is a candidate for temperature tolerance during wheat meiosis
Source: Theor Appl Genet. 2019 Dec 18;133(3):809–28. doi: 10.1007/s00122-019-03508-9 (PMC7021665; doi:10.1007/s00122-019-03508-9)
Supplement: Supplementary file 4 — Multiple sequence alignment of the promoter regions of the three hexaploid wheat Dmc1 homeologs: TraesCS5A02G133000 (TaDmc1-A1) on chromosome 5A, TraesCS5B02G131900 (TaDmc1-B1) on 5B and TraesCS5D02G141200 (TaDmc1-D1) on 5D. The promoter regions include the 1500 nucleotides downstream of the start codon of each of the TaDmc1 homeologs. Note the large insertion mutation (163 nt in size) at position 430 downstream of the start codon of TaDmc1-B1 (PDF 165 kb) [file 122_2019_3508_MOESM4_ESM.pdf]

```
                 *        20         *        40         *        60         *        80         *       100         *       120         *       140         *
TaDmc1-A1 : ATATGAAAAAGTAAAAAAACGCCGTGAAACCTATCAACAAAATAAAACACTTAAAGATTCAATCCAGTTGCAAAAAAATCACACAAGGAAGGAAGAAATATGCAA-------GCCAGCACAGAAGGGAAAAA--TGCGCATAAGAAAGAC :  141
TaDmc1-B1 : ----------------------------------------------------------------------------------------------------------------------------------------TGGGCCGGACGG-- :   12
TaDmc1-D1 : -------------------CGTCGTGAAACCTATCAACGAAATAAAACAATTAAAGATTCCACCCAGTTGCACAAAAAGCACACAAGGAAGGAAGAAAAATGCAAACATGGAGCCAACACAAAAAGGGAAAAAAATGCGCACAAGAAAGGC :  131

                160          *       180         *       200         *       220         *       240         *       260         *       280         *       300
TaDmc1-A1 : AGAAA-TATGCAAATACGCAGCCAAAACAAAAAGAAAGCTAGAATTGAGCAAATTGCAGCTACCCGTGAATTGAAAC-GCAACACACCATATAAATATTCAATTGT-GATGGAGAGCGCTGCATATATCG-CAACAGAAGAGGAAAGAGT :  287
TaDmc1-B1 : AGCAGTTGTAGGAAGTCGCAGCCCGTGGGTGGGGGGAGGGGGTACC-AGC--------GCCACCAGGGAGCTGGAGCCGTCGTGGGAGGGGGGACGCCCGCACCGCCGGTGGAGACCACCGCCGCCGTAGGCACCAGATCCGGGGGAGG :  153
TaDmc1-D1 : AGAAAATATGCAAATATGCAGCCAGAACAAAAGGAA-GCTAGAATTGAGCAAATTGCAGCTAACCATGAATTGAAGC-GCAACA--GCATGCAAAGGTTCAATTGT-GATGGAGAGCCTTGCATATATCA-CAACAGAAGAGGAAAGAGT :  275

                 *       320         *       340         *       360         *       380         *       400         *       420         *       440         *
TaDmc1-A1 : AAACCTATACGGCGAGGATTTCGTGGTTAGCACACACACAACGGAAGCATGTATGCATGGACGTCCGATGTGTGCAACATTTTCATTGTATGATGTTAGTAACTCAATAGAAATGCTGCATGTATGACATTTTTATTTTCTGTATAATGC :  437
TaDmc1-B1 : GG---CGAAGGGAGAG-GGTTGGTGG--AGAAAGGCCCCGCCGCCCACCATCC---TAGGCCCCGGCGCGGCTTT--GCCGGCCCGCCCTCCGGCGATGGCAACGCGG-GGTGGCGGGAGGCGAGCGGACGAGCCGACGCCGGCGGGGCT :  291
TaDmc1-D1 : AAATCTATACGACGAG-ATTTCGTGGTTAGCACACACACAACAGAAGCATACATGCATGGACAGCCGATGTATACAACATTTTTATTGTATGATGTTAGTAACTCAATAGAAATGTTGCATATACGACATTTTTATTAATGTGTATAATGC :  424

                460          *       480         *       500         *       520         *       540         *       560         *       580         *       600
TaDmc1-A1 : TTAATATGCATTGTATACTA-ACCTTCTGTTATGGTTGATAATCTATATCTTTGCACTTATATTTGGTTTGTTTGTTGAGATATACGCACACTATAAACCTAAGGATGGTAGTCAACCGTGTCTATGCTTGATTATAGTACCGCAATAT :  586
TaDmc1-B1 : TTCCCCCGAGTTGCCCGGCACAAGCGACGCTAGGGTCGGGGGTCGGG--CGTGAGGTTCAGAGCAAGG---ACTTGTTGAGATATACGCACACTATGAACCTAAGGATGGTAGTCAACCGTGTCTATGCTTGATTATAGTACCGCAATAT :  436
TaDmc1-D1 : TTAATCTGCATTGTATACTA-ACCTTCTGTTATGGTTGATAATCTATATCTTTGCACTTATATTTGGTTTGTTTGTTGAGATATACGCACACTATAAACCTAAGGATGGCAGTCAACCATGTCTATACTGGATTATAGTACCGCAGTAT :  573

                 *       620         *       640         *       660         *       680         *       700         *       720         *       740         *
TaDmc1-A1 : GCTTTGTCAGTGACAACCTAGCACATGCTTGATTATAGTACGCATTATTCCTATTGAAAGATGTATTAAATCTGAAATATTCATTCTTCTGATAATGAAATGAAGGCGAGAAATAATACCTTGCATTGGCAGAAGAAATTATGTTTCAGT :  736
TaDmc1-B1 : GCTATGTCAGTGACAACCTAGCACATGCTTGATTATAGCACACATTATTCCTATTGAAAAATGTATCAAATTTGAAATATTCATTCTTCCGATAATGAAATGAAGGCGAGAAATAATACCTTGCATTGGCAGAAGAAATTATGTTTCAGT :  586
TaDmc1-D1 : GCTTTGTCAGTGACAACCTAGCACATGCTTGATTATAGTACACACTATTCCTATTGAAAGATGTATTAAATCTGAAATATTCATTCTTCTGATAATGAAATGAAGGCGAGAAATAATACCTTGCATTGGCAGAAGAAATTATGCTTCGGT :  723

                760          *       780         *       800         *       820         *       840         *       860         *       880         *       900
TaDmc1-A1 : AATTCCAATTTTGTTATCTTTTGTATGTCTTTTTATTCTTATCTTGAGTTTATTTTTTT-CATTTCTGTTAGAGATGATTGTCTGTAACTCTTTTT-TTAGTAGGTGATTGTCTTTAACCTAACCCGCAAATTGTACAATGCATAAGTTT :  884
TaDmc1-B1 : AATTTCAATTTTGTTATCTTTTGTATGTCTTTTTATTCTTATGTTGAGTTTATTTTTTTTCATTTCTGTTAGAGATTATTGTCTTTAACCCTTTTT---TATAGGTGATTGTCTTTAACCTAACCCGCAAATTGTACAATGCATAAGTTT :  733
TaDmc1-D1 : AATTTCAATTTTGTTATCTTTTGTATGTCTTTTTATTCCTATGTTGAGTTTATTTTTTT-CATTTCTGTTAGAGATGATTGTCTTTAACCCCTTTTCTTTGTAGGTGATTGTCTTTAACCTAACCCGCAAATTATACAATGCATAAGTTT :  872

                 *       920         *       940         *       960         *       980         *      1000         *      1020         *      1040         *
TaDmc1-A1 : GTTAGCCGTTTGCAAAGATTGGTTGGCTAAAATCACAATTTTTCTTCATGTACATTCCCATTACCTTGCATATTGGGTTT-AGGAGCGTGTGTTTTTCTCCCACTGCAACACACATGCATGTTTGCTAGTAAACCTAACGAGAGGATACA : 1033
TaDmc1-B1 : GTTAGCCGTTTGTAAAGATTGGTTGGCTAAAATCACAATTCTCGTTCATGTACATTTTCATCACCATGCATGTTGGGTTT-AGGAGCGTGTGTTTTTCTCCCATTATAACGCACATGCATGTTTGCTAGTAAATCTAATGAGAGGGTACA :  882
TaDmc1-D1 : GTTAGCCGTTTGCAAAGATTGGTTGGCCGAACTCACAATTCTCCTTCATGTACATTCCCATCACCATGCATATTGGGTTTTAGGAGCGTGTGTTTTTCTCCAATTGCAACACACATGCATGTTTGCTAGTTA-CCTAACGAGAGGGTACA : 1021

               1060          *      1080         *      1100         *      1120         *      1140         *      1160         *      1180         *      1200
TaDmc1-A1 : CTTTTATTTTAACATCAAAGTACTT----------------------------------------------------------------------------------------------------------------------------- : 1058
TaDmc1-B1 : CTTTTATTTTGACATCAAAGTACTTCCTCCGTTCTTAAATATTTGTCCTTTTAGAGATTTCAAATGGACTACCACATACCGGATGTATATAGACATATTTAGAGTGTAGATTCACTTATTTGCTCCGTATGTAGTCACTTGTTGAAAT : 1032
TaDmc1-D1 : CTTTTATTTTAACATCAAAATACTT----------------------------------------------------------------------------------------------------------------------------- : 1046

                 *      1220         *      1240         *      1260         *      1280         *      1300         *      1320         *      1340         *
TaDmc1-A1 : --------------------------------------TTTTATTGCAAGGTGATCACCCTAACAAGA----ACACCTTTTACAAAAGAGTTGGTATATCAAACGGGTTAAGAATCAAAGCTGTTGTTACTAG---ATTGCTTGATTGGC : 1163
TaDmc1-B1 : CTCTAGAAAAATAAATATTTAAGAACGAAGGGAGTACTTTTTATTGCAAGGTGATCACCCTAACAAGAGGGGACGCCTTTTACAAAAGAGTTGGTACATCAAACGGGTTAAGAACCAAAGCGGTTGTTACTAGTAGATTGCTTGATTGGC : 1182
TaDmc1-D1 : --------------------------------------TTT-ATTGCAAGGCCATCACCCTAACAAGAGGGAACACCTTTTACAAAAGAGTTGGTATATCAACCGGGTTAAGAATCAAAGCGGTTCTTACTAG---ATTGCTTGATTGGC : 1154

               1360          *      1380         *      1400         *      1420         *      1440         *      1460         *      1480         *      1500
TaDmc1-A1 : ACGTCGCCGTCCTGTTCAAAATTTTGAATTTCGAATGGGCCGTGGAAGGCTTAGCTGGCAGCAGCAACAAGTGTTGG------------GCTCTCAAGTTCCTCGCGCGAGGCCTTCAAATA-CCCCCACCTCGTCGCAGCCGCAGTGAT : 1300
TaDmc1-B1 : ACGTCGGCGTCCTGTTCAAAGTTTTGAATTTCGAATGGGCCGTGCAAGGCTTAGCTGGCAGGA---AC---------------------------AAGTTGCTCGCGCGAGGCCTTCAAATA-CCCCCACCTCGTCGCAGCCGCGGTGAT : 1301
TaDmc1-D1 : ACGTCGGCGTCCTGTTCAAAATTTTGAATTTTGAATGGGCCGTAAAAGGCTTAGCTGGCAGCAGCAACGAGCGTGGCTTGGTGTTACGGCTCACAAGTTCCTCGTGCGAGGCCTTCAAATAACCCCCACCTCGTCGCAGCCGCAGTGAT : 1304

                 *      1520         *      1540         *      1560         *      1580         *      1600         *      1620         *      1640         *
TaDmc1-A1 : CCACATTCCACCCGCACCTGCGCTTCCCAAACCTCCCCTCTCCCCCAGCCTCCGCCCCACTTCCTTCTCCTCCAGCAGCACGCGATCCTCCGGCTCGTACAGGTCTCGTCCTCCTCCACCGCTTCCACTCGCCGTCTTCGCTCGTGCGTC : 1450
TaDmc1-B1 : CCACATTCCACCCGCACCTGCGCTTCCCAAACCTCCCCTCTCCCCCAGCCTTGGCCCCACTTCCT-CTCCTCTAGCAGCACACGATCCTCTGCCTCGTACAGGTCTCGTCCTCCTCCACCGCTTCCACTCGCCGTCTTCGCTCGTGCGTC : 1450
TaDmc1-D1 : CCACATTCCACCCGCACCTGCGCTTCCCAAACCTCCCCTCTCCCCCAGCCTCCGCCCCACTTC----TCCTCCAGCAGCACGCGAACCTCCGCCTCTTACAGGTCTCGTCCTCCTCCACCGCTTCCACTCGCCGTCTTCGCTCCGCTGTC : 1450

               1660          *      1680         *      1700
TaDmc1-A1 : CCAGTGCTCGCTCCTTACACCTTCTCCTGTGACGCAGGCGCGCACGCGGGATG : 1503
TaDmc1-B1 : CCAGAGCTCGCTCCTGACACCTTTTCCTCTGACGCAGGCGGGCACGCGGGATG : 1503
TaDmc1-D1 : CCAGTGCTCGCTCCTGACACCTTCTCCTCTGACGCAGGCGGACACGCGGGATG : 1503
```
