# Supplementary material for: Dmc1 is a candidate for temperature tolerance during wheat meiosis
Source: Theor Appl Genet. 2019 Dec 18;133(3):809–28. doi: 10.1007/s00122-019-03508-9 (PMC7021665; doi:10.1007/s00122-019-03508-9)
Supplement: Supplementary file 5 — Multiple alignment of Dmc1 DNA sequences from T. aestivum and its diploid and tetraploid ancestors, T. urartu (AA), Ae. tauschii (DD) and T. dicoccoides (AABB); note the large insertion of 294 bp in the B-genome copy of the T. aestivum gene (DOCX 33 kb) [file 122_2019_3508_MOESM5_ESM.docx]

* 20 * 40 * 60 * 80 * 100 * 120 * 140 * 160
*T.aestivum*_A : ATGGCGCCGTCCAAGCAGTACGACGAGGGCGGGCAGCTCCAGCTCATGGAGGCCGACCGGGTCGAGGAGGAGGAGGAGTGCTTCGAGTCCATCGACAAGTGTACGTTCGCCGCCTCCTACCCCTCTCCTCTCGAAA--CCCGCCCGCTGCCCCGTCTCCGGTGCTGCAT : 167
*T.dicoccoides*_A : ATGGCGCCGTCCAAGCAGTACGACGAGGGCGGGCAGCTCCAGCTCATGGAGGCCGACCGGGTCGAGGAGGAGGAGGAGTGCTTCGAGTCCATCGACAAGTGTACGTTCGCCGCCTCCTACCCCTCTCCTCTCGAAA--CCCGCCCGCTGCCCCGTCTCCGGTGCTGCAT : 167
*T.urartu*_A : ATGGCGCCGTCCAAGCAGTACGACGAGGGCGGGCAGCTCCAGCTCATGGAGGCCGACCGGGTCGAGGAGGAGGAGGAGTGCTTCGAGTCCATCGACAAGTGTACGTTCGCCGCCTCCTACCCCTCTCCTCTCGAAA--CCCGCCCGCTGCCCCGTCTCCGGTGCTGCAT : 167
*T.aestivum*_D : ATGGCGCCGTCCAAGCAGTACGACGAGGGCGGGCAGCTCCAGCTCATGGAGGCCGACCGGGTGGAGGAGGAGGAGGAGTGCTTCGAGTCCATCGACAAGTGTACGTTCGCCGCCTCCAACCCCTCTCCTCTCCAAA--CCCTCCCGCCGCCCCGTCTCCGGTGCTGCAT : 167
*Ae.tauschii*_D : ATGGCGCCGTCCAAGCAGTACGACGAGGGCGGGCAGCTCCAGCTCATGGAGGCCGACCGGGTGGAGGAGGAGGAGGAGTGCTTCGAGTCCATCGACAAGTGTACGTTCGCCGCCTCCAACCCCTCTCCTCTCCAAAAACCCTCCCGCCGCCCCGTCTCCGGTGCTGCAT : 169
*T.aestivum*_B : ATGGCGCCGTCCAAGCAGTACGACGAGGGCGGGCAGCTCCAGCTCATGGAGGCCGACCGGGTCGAGGAGGAGGAGGAGTGCTTCGAGTCCATCGACAAGTGTATGTTCGCCGCCTCCAACTCCTCTCCTCTCGAAA--CCCTCCCGCCGCCCCGTCTCCGGTGCTGCAT : 167
*T.dicoccoides*_B : ATGGCGCCGTCCAAGCAGTACGACGAGGGCGGGCAGCTCCAGCTCATGGAGGCCGACCGGGTCGAGGAGGAGGAGGAGTGCTTCGAGTCCATCGACAAGTGTATGTTCGCCGCCTCCAACTCCTCTCCTCTCGAAA--CCCTCCCGCCGCCCCGTCTCCGGTGCTGCAT : 167
 ATGGCGCCGTCCAAGCAGTACGACGAGGGCGGGCAGCTCCAGCTCATGGAGGCCGACCGGGT GAGGAGGAGGAGGAGTGCTTCGAGTCCATCGACAAGTGTA GTTCGCCGCCTCC AC CCTCTCCTCTC AAA CCC CCCGC GCCCCGTCTCCGGTGCTGCAT

 * 180 * 200 * 220 * 240 * 260 * 280 * 300 * 320 * 3
*T.aestivum*_A : TTACTTGCTTGTTCGTGTGCCTGCGTCGCGCGTGTGTCGGCATGTGGGGGTTAGGCCTGCTCACCGTTGCGTTCCGGGTGCTTCCGCCTCTAAGTTCGCGCGTTTCGGTCGCAATTTCGTGCTGTTTGGAGATGGGTTTGGTGCGGATTTCGCTTAGCCTCCACATTTG : 336
*T.dicoccoides*_A : TTACTTGCTTGTTCGTGTGCCTGCGTCGCGCGTGTGTCGGCATGTGGGGGTTAGGCCTGCTCACCGTTGCGTTCCGGGTGCTTCCGCCTCTAAGTTCGCGCGTTTCGGTCGCAATTTCGTGCTGTTTGGAGATGGGTTTGGTGCGGATTTCGCTTAGCCTCCACATTTG : 336
*T.urartu*_A : TTACTTGCTTGTTCGTGTGCCTGCGTCGCGCGTGTGTCGGCATGTGGGGGTTAGGCCTGCTCACCGTTGCGTTCCGGGTGCTTCCGCCTCTAAGTTCGCGCGTTTCGGTCGCAATTTCGTGCTGTTTGGAGATGGGTTTGGTGCGGATTTCGCTTAGCCTCCACATTTG : 336
*T.aestivum*_D : TTACTTGCTTGTTCGTGTGCCTGCGTCGCGCGCGTGTCGGCGCGTGGGGGTTAGGCCTGCTCACCGTTGCGTCCCGGGTGCTTCCGCCTCTAAGTTCGCGCGTTTCGGTCGCAATTTCGTGCTGTTTGGAGATGGATTTGGTGCGGATTTCGCTTAGCCTCCACATTTG : 336
*Ae.tauschii*_D : TTACTTGCTTGTTCGTGTGCCTGCGTCGCGCGCGTGTCGGCGCGTGGGGGTTAGGCCTGCTCACCGTTGCGTCCCGGGTGCTTCCGCCTCTAAGTTCGCGCGTTTCGGTCGCAATTTCGTGCTGTTTGGAGATGGATTTGGTGCGGATTTCGCTTAGCCTCCACATTTG : 338
*T.aestivum*_B : TTACTTGCTTGTCCGTGTGCCTGCGTCGCGCGCGTGTCGGCGCGTGGGGGTTAGGCCTGCTCACCGTTGCGTTCCGGGTGCTTCTGCCTCTAAGTTCGCGCGTTTCGGTCACAATTTCGTGCTGTTTGGAGATGGATTTGGTGCGGATTTCGCTTAGCCTCCACATTTG : 336
*T.dicoccoides*_B : TTACTTGCTTGTCCGTGTGCCTGCGTCGCGCGCGTGTCGGCGCGTGGGGGTTAGGCCTGCTCACCGTTGCGTTCCGGGTGCTTCTGCCTCTAAGTTCGCGCGTTTCGGTCACAATTTCGTGCTGTTTGGAGATGGATTTGGTGCGGATTTCGCTTAGCCTCCACATTTG : 336
 TTACTTGCTTGT CGTGTGCCTGCGTCGCGCG GTGTCGGC GTGGGGGTTAGGCCTGCTCACCGTTGCGT CCGGGTGCTTC GCCTCTAAGTTCGCGCGTTTCGGTC CAATTTCGTGCTGTTTGGAGATGG TTTGGTGCGGATTTCGCTTAGCCTCCACATTTG

 40 * 360 * 380 * 400 * 420 * 440 * 460 * 480 * 500
*T.aestivum*_A : GTTGGTTTTTGGTGCGTGCAGCGCGTGGTCGCGTGTGCTCGTGTTGCGTTTGTTTTGATTTTCTCCCCTATTTCGTGCAGTCTGGGGATTAACTAGCGCCCTT-GGTAGGCTAATTTCGTGCAGCTTTGCTGATTCCCCCCACTTGATTTCTCTTTACCGGTTAGTTTG : 504
*T.dicoccoides*_A : GTTGGTTTTTGGTGCGTGCAGCGCGTGGTCGCGTGTGCTCGTGTTGCGTTTGTTTTGATTTTCTCCCCTATTTCGTGCAGTCTGGGGATTAACTAGCGCCCTT-GGTAGGCTAATTTCGTGCAGCTTTGCTGATTCCCCCCACTTGATTTCTCTTTACCGGTTAGTTTG : 504
*T.urartu*_A : GTTGGTTTTTGGTGCGTGCAGCGCGTGGTCGCGTGTGCTCGTGTTGCGTTTGTTTTGATTTTCTCCCCTATTTCTTGCAGTCTGGGGATTAACTAGCGCCCTT-GGTAGGCTAATTTCGTGCAGCTTTGCTGATTCCCCCCACTTGATTTCTCTTTACCGGTTAGTTTG : 504
*T.aestivum*_D : GTTGGTTTTTGGTGCGTGCAGCGCGTGGTCG--TGTGCTCCTGTTGCGTTGGTTTTGATTTTCTCCCCTATGTCGTGCAATCTGGGGATTAACTAGCGCCCTTTGGTAGCCTAATTTCGTGCAGCTTTGTTGATGCCCCCCACTTGATTTCTCTTTACCGGCTAGTTTG : 503
*Ae.tauschii*_D : GTTGGTTTTTGGTGCGTGCAGCGCGTGGTCG--TGTGCTCCTGTTGCGTTGGTTTTGATTTTCTCCCCTATGTCGTGCAATCTGGGGATTAACTAGCGCCCTTTGGTAGCCTAATTTCGTGCAGCTTTGTTGATGCCCCCCACTTGATTTCTCTTTACCGGCTAGTTTG : 505
*T.aestivum*_B : GTTGGTTTTTGGTGCGTGCAGCGCGTGGTCG--TGTGCTCGTGTTGCGTTGGTTTTGATTTTCTCCCCTATTTCGTGCAATCTGGGGATTAACTAGCGCCCTTTGGTAGCCTAATTTCGTGCGGCTTTTCTAATTCCCCCCACTTGATTTCTCTTTACCGGCTAGTTTG : 503
*T.dicoccoides*_B : GTTGGTTTTTGGTGCGTGCAGCGCGTGGTCG--TGTGCTCGTGTTGCGTTGGTTTTGATTTTCTCCCCTATTTCGTGCAATCTGGGGATTAACTAGCGCCCTTTGGTAGCCTAATTTCGTGCGGCTTTTCTAATTCCCCCCACTTGATTTCTCTTTACCGGCTAGTTTG : 503
 GTTGGTTTTTGGTGCGTGCAGCGCGTGGTCG TGTGCTC TGTTGCGTT GTTTTGATTTTCTCCCCTAT TCgTGCA TCTGGGGATTAACTAGCGCCCTT GGTAG CTAATTTCGTGC GCTTT T AT CCCCCCACTTGATTTCTCTTTACCGG TAGTTTG

 * 520 * 540 * 560 * 580 * 600 * 620 * 640 * 660 *
*T.aestivum*_A : TGCTAATCGCTGTGTGGTGCCGTTTTTTCTCCACGGATTAGTCTGAACTTAGCCCCAATTTTGAGCTGCCATGCGTTTAATCCGCCGTAGCGATCACTGTCTGTAGAATCCTTTCGAATTCGGCACTAGATACAGTTCAATCGCGTGGGTTTTCTGTGGAAACTTGGAC : 673
*T.dicoccoides*_A : TGCTAATCGCTGTGTGGTGCCGTTTTTTCTCCACGGATTAGTCTGAACTTAGCCCCAATTTTGAGCTGCCATGCGTTTAATCCGCCGTAGCGATCACTGTCTGTAGAATCCTTTCGAATTCGGCACTAGATACAGTTCAATCGCGTGGGTTTTCTGTGGAAACTTGGAC : 673
*T.urartu*_A : TGCTAATCGCTGTGTGGTGCCGTTTTTTCTCCACGGATTAGTCTGAACTTAGCCCCAATTTTGAGCTGCCATGCGTTTAATCCGCCGTAGCGATCACTGTCTGTAGAATCCTTTCGAATTCGGCACTAGATACAGTTCAATCGCGTGGGTTTTCTGTGGAAACTTGGAC : 673
*T.aestivum*_D : TGCTAATCGCTGTGTGGTGCCGTTTTTTCTCCACGAATTAGTCTGAATTTAGCCCCAATTTTGAGCTGCCATGCGTTTAATCTGCCGTAGCGATCACTGGCTGTAGAATCCTTTCGAATTCGAAACTAGATACAGTTCAATCGCGTGGGTTTTCTGTGGAAACTTGGAC : 672
*Ae.tauschii*_D : TGCTAATCGCTGTGTGGTGCCGTTTTTTCTCCACGAATTAGTCTGAATTTAGCCCCAATTTTGAGCTGCCATGCGTTTAATCTGCCGTAGCGATCACTGGCTGTAGAATCCTTTCGAATTCGAAACTAGATACAGTTCAATCGCGTGGGTTTTCTGTGGAAACTTGGAC : 674
*T.aestivum*_B : TGCTAATCGCTGTGTGGTGCCGTTTTTTCTCCACGGATTAGTCTGAACTTAGCCCCAATTTTGAGCTGCCATGCCTTTAATCCGCCGTATCGATCACTGGCTGTAGAATCCTGTCGAATTCGAAACTAGATACAGTTCAATCGCGTGGGTTTTCTGTGGAAGCTTGGAC : 672
*T.dicoccoides*_B : TGCTAATCGCTGTGTGGTGCCGTTTTTTCTCCACGGATTAGTCTGAACTTAGCCCCAATTTTGAGCTGCCATGCCTTTAATCCGCCGTATAGATCACTGGCTGTAGAATCCTGTCGAATTCGAAACTAGATACAGTTCAATCGCGTGGGTTTTCTGTGGAAGCTTGGAC : 672
 TGCTAATCGCTGTGTGGTGCCGTTTTTTCTCCACG ATTAGTCTGAA TTAGCCCCAATTTTGAGCTGCCATGC TTTAATC GCCGTA cGATCACTG CTGTAGAATCCT TCGAATTCG ACTAGATACAGTTCAATCGCGTGGGTTTTCTGTGGAA CTTGGAC

 680 * 700 * 720 * 740 * 760 * 780 * 800 * 820 * 840
*T.aestivum*_A : CAATTACGGACGCGTTTTCGTCCACTTCGTGGCGTAGGGTTT-AAGCCTGTCTTCCTCATCTGTTCCACTTTTTTTTTTCTGAATGTTCACCTGTTCTGTTCCATAATTACGGGTAGCTTCCGCGAGTTTAGCAAGTTTACTTTAAACATTTGTCTAGATGTGATCCGC : 841
*T.dicoccoides*_A : CAATTACGGACGCGTTTTCGTCCACTTCGTGGCGTAGGGTTT-AAGCCTGTCTTCCTCATCTGTTCCACTTTTTTTTTTCTGAATGTTCACCTGTTCTGTTCCATAATTACGGGTAGCTTCCGCGAGTTTAGCAAGTTTACTTTAAACATTTGTCTAGATGTGATCCGC : 841
*T.urartu*_A : CAATTACGGACGCGTTTTCGTCCACTTCGTGGCGTAGGGTTT-AAGCCTGTCTTCCTCATCTGTTCCACTTTTTTTTT-CTGAATGTTCACCTGTTCTGTTCCATAATTACGGGTAGCTTCCGCGAGTTTAGCAAGTTTACTTTAAACATTTGTCTAGATGTGATCCGC : 840
*T.aestivum*_D : CAATTACGGACGCGTTTTCCTGCACTTCGAGGCGTAGGGTTTTAAGCCTGTCTTCCTTATCTGTTCCACTTTTTTTTT-CTGAATGTTCACCTGTTATGTTCCATAATTACGGGTAGCTTCCGCGAGTTTAGCAAGTTTACTTTAAACATTTGCCTAGATGTGATCCGC : 840
*Ae.tauschii*_D : CAATTACGGACGCGTTTTCCTGCACTTCGAGGCGTAGGGTTTTAAGCCTGTCTTCCTTATCTGTTCCACTTTTTTTTT-CTGAATGTTCACCTGTTCTGTTCCATAATTACGGGTAGCTTCCGCGAGTTTAGCAAGTTTACTTTAAACATTTGCCTAGATGTGATCCGC : 842
*T.aestivum*_B : CAATTACGGACGCGTTTTCGTGCACTTCGAGGCGTAGGGTTTTAAGCCTGTCGTCCTCATCTGTTCCACTTTTTTTTTTCTGAATGTTCACCTGTTCTGTTCCATAATCACGGGTAGCTTCCGCGAGTTTAGCAAGTTTACTTTAAACATTTGCCTAGATGTGATCCGC : 841
*T.dicoccoides*_B : CAATTACGGACGCGTTTTCGTGCACTTCGAGGCGTAGGGTTTTAAGCCTGTCGTCCTCATCTGTTCCACTTTTTTTTT-CTGAATGTTCACCTGTTCTGTTCCATAATCACGGGTAGCTTCCGCGAGTTTAGCAAGTTTACTTTAAACATTTGCCTAGATGTGATCCGC : 840
 CAATTACGGACGCGTTTTC T CACTTCG GGCGTAGGGTTT AAGCCTGTC TCCT ATCTGTTCCACTTTTTTTTT CTGAATGTTCACCTGTTcTGTTCCATAAT ACGGGTAGCTTCCGCGAGTTTAGCAAGTTTACTTTAAACATTTG CTAGATGTGATCCGC

 * 860 * 880 * 900 * 920 * 940 * 960 * 980 * 1000 *
*T.aestivum*_A : GCTTGTGTTTTGTTTGCGTGATCCAATTGGTTCGTCGTGCACTTTGTTCATTGCCATGAATTTGAATCCTAAGCCGTAACCTTCCTTTTAACGCTGTAACTGATCACTCGTCCAAACAGTGATCTCGCAGGGAATAAACTCAGGAGACGTGAAGAAGCTGCAGGATGCG : 1010
*T.dicoccoides*_A : GCTTGTGTTTTGTTTGCGTGATCCAATTGGTTCGTCGTGCACTTTGTTCATTGCCATGAATTTGAATCCTAAGCCGTAACCTTCCTTTTAACGCTGTAACTGATCACTCGTCCAAACAGTGATCTCGCAGGGAATAAACTCAGGAGACGTGAAGAAGCTGCAGGATGCG : 1010
*T.urartu*_A : GCTTGTGTTTTGTTTGCGTGATCCAATTGGTTCGTCGTGCACTTTGTTCATTGCCATGAATTTGAATCCTAAGCCGTAACCTTCCTTTTAACGCTGTAACTGATCACTCGTCCAAACAGTGATCTCGCAGGGAATAAACTCAGGAGACGTGAAGAAGCTGCAGGATGCG : 1009
*T.aestivum*_D : GCTTGTGTTTTGTTTGCGTGATCCAATTGGTTCGTCGTGCACTTTGTTCATTGCCATGAATTTGAATCCTAAGCCGTAACATTCCTTGTAACGCTGTAACTAATCACTCGTCCAAACAGTGATCTCGCAGGGAATAAACTCAGGAGACGTGAAGAAGCTGCAGGATGCG : 1009
*Ae.tauschii*_D : GCTTGTGTTTTGTTTGCGTGATCCAATTGGTTCGTCGTGCACTTTGTTCATTGCCATGAATTTGAATCCTAAGCCGTAACATTCCTTGTAACGCTGTAACTAATCACTCGTCCAAACAGTGATCTCGCAGGGAATAAACTCAGGAGACGTGAAGAAGCTGCAGGATGCG : 1011
*T.aestivum*_B : GCTTGTGTTTTGTTTGCGTGATCCAATTGGTTCGTCGTGCACTTTGTTCATTGCCATGAATTTGAATCCTAAGCCGTAAC------------GCTGTAACTAATCACTCGTCCAAACAGTGATCTCGCAGGGAATTAACTCAGGAGACGTGAAGAAGCTGCAGGATGCG : 998
*T.dicoccoides*_B : GCTTGTGTTTTGTTTGCGTGATCCAATTGGTTCGTCGTGCACTTTGTTCATTGCCATGAATTTGAATCCTAAGCCGTAAC------------GCTGTAACTAATCACTCGTCCAAACAGTGATCTCGCAGGGAATTAACTCAGGAGACGTGAAGAAGCTGCAGGATGCG : 997
 GCTTGTGTTTTGTTTGCGTGATCCAATTGGTTCGTCGTGCACTTTGTTCATTGCCATGAATTTGAATCCTAAGCCGTAAC GCTGTAACT ATCACTCGTCCAAACAGTGATCTCGCAGGGAAT AACTCAGGAGACGTGAAGAAGCTGCAGGATGCG

 1020 * 1040 * 1060 * 1080 * 1100 * 1120 * 1140 * 1160 * 1180
*T.aestivum*_A : GGGATCTACACTTGCAATGGGCTGATGATGCACACCAAGAAGGTCCCAATCCCGTCAGAGCATAAATCTGCACGCATTCTCCCTAAAATTTGTGGTTGCATACTGAAGCTGATTTCTGGTGCACGACACATGACTGTTATTAGTTGCTTTATGTATTCAAGTTTCAACA : 1179
*T.dicoccoides*_A : GGGATCTACACTTGCAATGGGCTGATGATGCACACCAAGAAGGTCCCAATCCCGTCAGAGCATAAATCTGCACGCATTCTCCCTAAAATTTGTGGTTGCATACTGAAGCTGATTTCTGGTGCACGACACATGACTGTTATTAGTTGCTTTATGTATTCAAGTTTCAACA : 1179
*T.urartu*_A : GGGATCTACACTTGCAATGGGCTGATGATGCACACCAAGAAGGTCCCAATCCCGTCAGAGCATAAATCTGCATGCATTCTCCCTAAAATTTGTGGTTGCATACTGAAGCTGATTTCTGGTGCACGACACATGACTGTTATTAGTTGCTTTATGTATTCAAGTTTCAACA : 1178
*T.aestivum*_D : GGGATCTACACTTGCAATGGGCTGATGATGCACACCAAGAAGGTCAAAATCCCTTCAGAGCCTAAATCTGCACGCATTCTCCCTAAAATTTGTGGTTGCATGCTGAAGTTGATTTCTGGTGCACGACACATGACTGTTATTAGTAGCTTTATGTATTCAAGTTTCAACA : 1178
*Ae.tauschii*_D : GGGATCTACACTTGCAATGGGCTGATGATGCACACCAAGAAGGTCAAAATCCCTTCAGAGCCTAAATCTGCACGCATTCTCCCTAAAATTTGTGGTTGCATGCTGAAGTTGATTTCTGGTGCACGACACATGACTGTTATTAGTAGCTTTATGTATTCAAGTTTCAACA : 1180
*T.aestivum*_B : GGGATCTACACTTGCAATGGCCTGATGATGCACACCAAGAAGGTCAAACTCCCTTCAGAGCCTAAATCTGCATGCATTCTCCCTAAAATTTGTGGTTGCATACTGAAGTTGATTTCTGGTGCACGACACATGACTGTTATTAGTTGTTTTATGTATTCAAGTTTCAACA : 1167
*T.dicoccoides*_B : GGGATCTACACTTGCAATGGCCTGATGATGCACACCAAGAAGGTCAAACTCCCTTCAGAGCCTAAATCTGCATGCATTCTCCCTAAAATTTGTGGTTGCATACTGAAGTTGATTTCTGGTGCACGACACATGACTGTTATTAGTTGTTTTATGTATTCAAGTTTCAACA : 1166
 GGGATCTACACTTGCAATGG CTGATGATGCACACCAAGAAGGTC A TCCC TCAGAGC TAAATCTGCA GCATTCTCCCTAAAATTTGTGGTTGCAT CTGAAG TGATTTCTGGTGCACGACACATGACTGTTATTAGT G TTTATGTATTCAAGTTTCAACA

 * 1200 * 1220 * 1240 * 1260 * 1280 * 1300 * 1320 * 1340 *
*T.aestivum*_A : CATGATTGATGTTGTTTCAGTACCAATGTATGGATTCATCTTGCAGAGCCTTACAGGGATTAAGGGCTTGTCTGAAGCAAAGGTTGATAAGATCTGCGAGGCTGCTGAAAAACTTCTGGTATGATTGTTATCATCTTTGCATTGATTCTGGTTTACAACTTCTGTGCCA : 1348
*T.dicoccoides*_A : CATGATTGATGTTGTTTCAGTACCAATGTATGGATTCATCTTGCAGAGCCTTACAGGGATTAAGGGCTTGTCTGAAGCAAAGGTTGATAAGATCTGCGAGGCTGCTGAAAAACTTCTGGTATGATTGTTATCATCTTTGCATTGATTCTGGTTTACAACTTCTGTGCCA : 1348
*T.urartu*_A : CATGATTGATGTTGTTTCAGTACCAATGTATGGATTCATCTTGCAGAGCCTTACAGGGATTAAGGGCTTGTCTGAAGCAAAGGTTGATAAGATCTGCGAGGCTGCTGAAAAACTTCTGGTATGATTGTTATCATCTTTGCATTGATTCTGGTTTACAACTTCTGTGCCA : 1347
*T.aestivum*_D : CATGATTGATGTTGTTTCAGTATCAATGTACGGATTCATCTTGCAGAGCCTTACAGGGATTAAGGGCTTGTCTGAAGCGAAGGTTGATAAGATCTGTGAGGCTGCTGAAAAACTTCTGGTATGATTGTTATCATCTTTGCATTGATTCTGGTTTAGAACTTCTGTGCCA : 1347
*Ae.tauschii*_D : CATGATTGATGTTGTTTCAGTATCAATGTACGGATTCATCTTGCAGAGCCTTACAGGGATTAAGGGCTTGTCTGAAGCGAAGGTTGATAAGATCTGTGAGGCTGCTGAAAAACTTCTGGTATGATTGTTATCATCTTTGCATTGATTCTGGTTTAGAACTTCTGTGCCA : 1349
*T.aestivum*_B : CATGATTGATGTTGTTTCAGTATCAATGTATGGATTCATCTTGCAGAGCCTTACAGGGATTAAGGGCTTGTCTGAAGCAAAGGTTGATAAGATCTGCGAGGCTGCTGAAAAACTTCTGGTATGATTGTTATCATCTTTGCATTGATTCTGGTTTAGAACTTCTGTGCCA : 1336
*T.dicoccoides*_B : CATGATTGATGTTGTTTCAGTATCAATGTATGGATTCATCTTGCAGAGCCTTACAGGGATTAAGGGCTTGTCTGAAGCAAAGGTTGATAAGATCTGCGAGGCTGCTGAAAAACTTCTGGTATGATTGTTATCATCTTTGCATTGATTCTGGTTTAGAACTTCTGTGCCA : 1335
 CATGATTGATGTTGTTTCAGTA CAATGTA GGATTCATCTTGCAGAGCCTTACAGGGATTAAGGGCTTGTCTGAAGC AAGGTTGATAAGATCTG GAGGCTGCTGAAAAACTTCTGGTATGATTGTTATCATCTTTGCATTGATTCTGGTTTA AACTTCTGTGCCA

 1360 * 1380 * 1400 * 1420 * 1440 * 1460 * 1480 * 1500 * 1520
*T.aestivum*_A : TATTATTCTCTTTGCATTGATTCTGGTTTACAACTTCTGTGCCATATTATTCTCTTTGCATTGATTCTGGTTTACAACTTCTGTGCCAT--------------------------------------------CTGTGCTATGCGATCGAGTCTTCTGAAATTTGTATT : 1473
*T.dicoccoides*_A : TATTATTCTCTTTGCATTGATTCTGGTTTACAACTTCTGTGCCATATTATTCTCTTTGCATTGATTCTGGTTTACAACTTCTGTGCCATATTATTCTCTTTGCATTGATTCTGGTTTACAACTTCTGTGCCATCTGTGCTATGCGATCGAGTCTTCTGAAATTTGTATT : 1517
*T.urartu*_A : TATTATTCTCTTTGCATTGATTCTGGTTTACAACTTCTGTGCCAT----------------------------------------------------------------------------------------CTGTGCTATGCGATCGAGTCTTCTGAAATTTGTATT : 1428
*T.aestivum*_D : TATTATTCTCTTTGCATTGATTCTGGTTTACAACCTCTGTGCCAT----------------------------------------------------------------------------------------CTGTGCTATGCGATAGAATCTTCTGAAGTTTGTGTA : 1428
*Ae.tauschii*_D : TATTATTCTCTTTGCATTGATTCTGGTTTACAACCTCTGTGCCAT----------------------------------------------------------------------------------------CTGTGCTATGCGATAGAATCTTCTGAAGTTTGTGTA : 1430
*T.aestivum*_B : TATTATTCTCTTTGCATTGATTCTGGTTTACAACTTCTGTGACAT----------------------------------------------------------------------------------------CTGTGCTATACGATCGAGTCTTCTGAAATTTGTATT : 1417
*T.dicoccoides*_B : TATTATTCTCTTTGCATTGATTCTGGTTTACAACTTCTGTGACAT----------------------------------------------------------------------------------------CTGTGCTATACGATCGAGTCTTCTGAAATTTGTATT : 1416
 TATTATTCTCTTTGCATTGATTCTGGTTTACAAC TCTGTG CAT CTGTGCTAT CGAT GA TCTTCTGAA TTTGT T

 * 1540 * 1560 * 1580 * 1600 * 1620 * 1640 * 1660 * 1680 *
*T.aestivum*_A : CTATTTGATTTTCAGAGTCAGGGTTTCATGACAGGAAGTGATCTCCTTATTAAGGTAAGGTTTAGGGAGCTAAGCTACTGATGAAGGACGACCATACAGTTTAGTGCTGTTTTTGCAGTAGTGCTACACCGCTACTATGCTAAACTTACTGTAA----TAGTTTGTTTT : 1638
*T.dicoccoides*_A : CTATTTGATTTTCAGAGTCAGGGTTTCATGACAGGAAGTGATCTCCTTATTAAGGTAAGGTTTAGGGAGCTAAGCTACTGATGAAGGACGACCATACAGTTTAGTGCTGTTTTTGCAGTAGTGCTACACCGCTACTATGCTAAACTTACTGTAA----TAGTTTGTTTT : 1682
*T.urartu*_A : CTATTTGATTTTCAGAGTCAGGGTTTCATGACAGGAAGTGATCTCCTTATTAAGGTAAGGTTTAGGGAGCTAAGCTACTGATGAAGGACGACCATACAGTTTAGTGCTGTTTTTGCAGTAGTGCTACACCGCTACTATGCTAAACTTACTGTAA----TAGTTTGTTTT : 1593
*T.aestivum*_D : CTATTTGATTTTCAGAGTCAGGGTTTCATGACAGGAAGTGATCTCCTTATTAAGGTGAGGTTTAGG-AGCTAAGCTACTGATTAAGGACGATCATACAGTTTAGTACTGTTTTGGCCATAGTGCTAC--------TATGCTAAACTTACTGTAAATAATAGTTTGTTTT : 1588
*Ae.tauschii*_D : CTATTTGATTTTCAGAGTCAGGGTTTCATGACAGGAAGTGATCTCCTTATTAAGGTGAGGTTTAGG-AGCTAAGCTACTGATTAAGGACGATCATACAGTTTAGTACTGTTTTGGCCATAGTGCTAC--------TATGCTAAACTTACTGTAAATAATAGTTTGTTTT : 1590
*T.aestivum*_B : CTATTTGATTTTCAGAGTCAGGGTTTCATGACAGGAAGTGATCTCCTTATTAAGGTAAGGTTTAGGGAGCTAAGCTACTGATGAAGGACGATCATACAGTTTAGTGCTGTTTTTGCAGTAGTGCTACACTGCTACTATGCTAAACTTACTGTTA----TAGTTTGTTTT : 1582
*T.dicoccoides*_B : CTATTTGATTTTCAGAGTCAGGGTTTCATGACAGGAAGTGATCTCCTTATTAAGGTAAGGTTTAGGGAGCTAAGCTACTGATGAAGGACGATCATACAGTTTAGTGCTGTTTTTGCAGTAGTGCTACACTGCTACTATGCTAAACTTACTGTAA----TAGTTTGTTTT : 1581
 CTATTTGATTTTCAGAGTCAGGGTTTCATGACAGGAAGTGATCTCCTTATTAAGGT AGGTTTAGG AGCTAAGCTACTGAT AAGGACGA CATACAGTTTAGT CTGTTTT GC TAGTGCTAC TATGCTAAACTTACTGTaA TAGTTTGTTTT

 1700 * 1720 * 1740 * 1760 * 1780 * 1800 * 1820 * 1840 * 186
*T.aestivum*_A : TGGAATCTGCTTGAGTGCTTTCATTACTCCACCTGTGTACTGCCCAGTTGTTTGATTTACTTTTTCGTATATTGAAGCGAAAGTCTGTTGTCCGGATTACCACTGGGAGCCAAGCGCTTGATGAGCTGCTTGGAGGTAACATATGTCGCCCTTGATTCTGTTCTGATTA : 1807
*T.dicoccoides*_A : TGGAATCTGCTTGAGTGCTTTCATTACTCCACCTGTGTACTGCCCAGTTGTTTGATTTACTTTTTCGTATATTGAAGCGAAAGTCTGTTGTCCGGATTACCACTGGGAGCCAAGCGCTTGATGAGCTGCTTGGAGGTAACATATGTCGCCCTTGATTCTGTTCTGATTA : 1851
*T.urartu*_A : TGGAATCTGCTTGAGTGCTTTCATTACTCCACCTGTGTACTGCCCAGTTGTTTGATTTACTTTTTCGTATATTGAAGCGAAAGTCTGTTGTCCGGATTACCACTGGGAGCCAAGCGCTTGATGAGCTGCTTGGAGGTAACATATGTCGCCCTTGATTCTGTTCTGATTA : 1762
*T.aestivum*_D : TGGAATCTGCTTGAGTGCTTTCATTACTCCACCTGTGTACTGCCCGGTTGTTTGATTTACTTTTCCGTATATTGAAGCGAAAGTCTGTTGTCCGGATTACCACTGGGAGCCAAACGCTTGATGAGCTGCTTGGAGGTAACATATGTCATCCTTGATTCTGTTCTGATTA : 1757
*Ae.tauschii*_D : TGGAATCTGCTTGAGTGCTTTCATTACTCCACCTGTGTACTGCCCGGTTGTTTGATTTACTTTTCCGTATATTGAAGCGAAAGTCTGTTGTCCGGATTACCACTGGGAGCCAAACGCTTGATGAGCTGCTTGGAGGTAACATATGTCATCCTTGATTCTGTTCTGATTA : 1759
*T.aestivum*_B : TGGAATCTGCTTGAGTGCTTTCATTACTCCACCTGTGTACTGCCCGGTTGTTTGATTTACTTTTCCGTATATTGAAGCGAAAGTCTGTTGTCCGGATTACCACTGGGAGCCAAGCGCTTGATGAGCTGCTTGGAGGTAACATATGTCGTTCTTGATTCTGTTCTGATTA : 1751
*T.dicoccoides*_B : TGGAATCTGCTTGAGTGCTTTCATTACTCCACCTGTGTACTGCCCGGTTGTTTGATTTACTTTTCCGTATATTGAAGCGAAAGTCTGTTGTCCGGATTACCACTGGGAGCCAAGCGCTTGATGAGCTGCTTGGAGGTAACATATGTCGTCCTTGATTCTGTTCTGATTA : 1750
 TGGAATCTGCTTGAGTGCTTTCATTACTCCACCTGTGTACTGCCC GTTGTTTGATTTACTTTT CGTATATTGAAGCGAAAGTCTGTTGTCCGGATTACCACTGGGAGCCAA CGCTTGATGAGCTGCTTGGAGGTAACATATGTC cCTTGATTCTGTTCTGATTA

 0 * 1880 * 1900 * 1920 * 1940 * 1960 * 1980 * 2000 * 2020
*T.aestivum*_A : TTTCTGATGTTATGCTCTAACCCATTCACATATTTCCATAATTTGAAGGAGGGATTGAAACACTCTGTATCACAGAGGCATTTGGAGAGTTCCGGTCAGTAAATGTTCCAGTTACCATTTTCTTCTGGATTT-----TTTTTTGCAGGGAGCGCCTATTAGTTTTATCT : 1971
*T.dicoccoides*_A : TTTCTGATGTTATGCTCTAACCCATTCACATATTTCCATAATTTGAAGGAGGGATTGAAACACTCTGTATCACAGAGGCATTTGGAGAGTTCCGGTCAGTAAATGTTCCAGTTACCATTTTCTTCTGGATTT-----TTTTTTGCAGGGAGCGCCTATTAGTTTTATCT : 2015
*T.urartu*_A : TTTCTGATGTTATGCTCTAACCCATTCACATATTTCCATAATTTGAAGGAGGGATTGAAACACTCTGTATCACAGAGGCATTTGGAGAGTTCCGGTCAGTAAATGTTCCAGTTACCATTTTCTTCTGGATTT-----TTTTTTGCAGGGAGCGCCTATTAGTTTTATCT : 1926
*T.aestivum*_D : TTCCTGATGTTATGCTCTAACCTATTCAAATATTTGCGTAATTTGAAGGAGGGATTGAAACACTCTGTATCACAGAGGCATTTGGAGAGTTCCGGTGAGTAAATGTTCCCGTTACCATTTTCTTCTTGGTTTCTTCTTTTTTTGCAGGGACCGCCTATTAGTTGTAGCT : 1926
*Ae.tauschii*_D : TTCCTGATGTTATGCTCTAACCTATTCAAATATTTGCGTAATTTGAAGGAGGGATTGAAACACTCTGTATCACAGAGGCATTTGGAGAGTTCCGGTGAGTAAATGTTCCCGTTACCATTTTCTTCTTGGTTTCTTCTTTTTTTGCAGGGACCGCCTATTAGTTGTAGCT : 1928
*T.aestivum*_B : TTTCTGATGTTATGCTCTAACCTATTCACATATTTCCATAATTTGAAGGAGGGATTGAAACACTCTGTATCACAGAGGCATTTGGAGAGTTCCGGTCAGTAAATGTTCCGGTTACCATTTTCTTCTGGATTTTT--TTTTTT-GCAGGGGGCGCCTATTAGTTGTATCT : 1917
*T.dicoccoides*_B : TTTCTGATGTTATGCTCTAACCTATTCACATATTTCCATAATTTGAAGGAGGGATTGAAACACTCTGTATCACAGAGGCATTTGGAGAGTTCCGGTCAGTAAATGTTCCCGTTACCATTTTCTTCTGGATTTTT--TTTTTTTGCAGGGGGCGCCTATTAGTTGTATCT : 1917
 TT CTGATGTTATGCTCTAACC ATTCA ATATTT C TAATTTGAAGGAGGGATTGAAACACTCTGTATCACAGAGGCATTTGGAGAGTTCCGGT AGTAAATGTTCC GTTACCATTTTCTTCT G TTT TTTTTtGCAGGG CGCCTATTAGTT TA CT

 * 2040 * 2060 * 2080 * 2100 * 2120 * 2140 * 2160 * 2180 * 2
*T.aestivum*_A : CTGTTATGTATGATTTGTGCCATATTTCGCAGTAGATGTAGCAATTTTTAGTCCTAGTAATTTTTTAGT------TGGTAAA-CTCTGATAAGCTGCTTTCACCTCCTTTTCTGCTGTCTAGTCTTCCAGAGGTATTGATGATGGTGCATAT----------------- : 2116
*T.dicoccoides*_A : CTGTTATGTATGATTTGTGCCATATTTCGCAGTAGATGTAGCAATTTTTAGTCCTAGTAATTTTTTAGT------TGGTAAAACTCTGATAAGCTGCTTTCACCTCCTTTTCTGCTGTCTAGTCTTCCAGAGGTATTGATGATGGTGCATAT----------------- : 2161
*T.urartu*_A : CTGTTATGTATGATTTGTGCCATATTTCGCAGTAGATGTAGCAATTTTTAGTCCCAGTAATTTTTTAGT------TGGTAAAACTCTGATAAGCTGCTTTCACCTCCTTTTCTGCTGTCTAGTCTTCCAGAGGTATTGATGATGGTGCATAT----------------- : 2072
*T.aestivum*_D : CTGTTATGTATGATTTGTGCCATATTTGGCAGTAG------------CTAGTCCTAGTAATTTTTTAGTACATGGTGCTAAAACTCTCATAAGCTTCTTTCACCTCCTTTTCTACTGTCTAGTCTTCCAGAGGTATTGATGGTAGTGCATATAAGAATTCGAAAGCATG : 2083
*Ae.tauschii*_D : CTGTTATGTATGATTTGTGCCATATTTGGCAGTAG------------CTAGTCCTAGTAATTTTTTAGTACATGGTGCTAAAACTCTCATAAGCTTCTTTCACCTCCTTTTCTACTGTCTAGTCTTCCAGAGGTATTGATGGTAGTGCATATAAGAATTCGAAAGCATG : 2085
*T.aestivum*_B : CTGTTATGTATGATTTGTGCCATATTTGGCAGTAGATGTAACAATTTTTAGTCCTAGTAATTTTTTAGT------TGGTAAAACTCTGATAAGCTTCTTTCACCTCCTTTTCTGCTGTCTAGCCTTCCAGAGGTATTGATGATAGTGCATATAACAACTCCTAAGCATG : 2080
*T.dicoccoides*_B : CTGTTATGTATGATTTGTGCCATATTTGGCAGTAGATGTAACAATTTTTAGTCCTAGTAATTTTTTAGT------TGGTAAAACTCTGATAAGCTTCTTTCACCTCCTTTTCTGCTGTCTAGCCTTCCAGAGGTATTGATGATAGTGCATATAACAACTCCTAAGCATG : 2080
 CTGTTATGTATGATTTGTGCCATATTT GCAGTAG TAGTCCtAGTAATTTTTTAGT TG TAAAaCTCT ATAAGCT CTTTCACCTCCTTTTCT CTGTCTAG CTTCCAGAGGTATTGATG T GTGCATAT

 200 * 2220 * 2240 * 2260 * 2280 * 2300 * 2320 * 2340 * 2360
*T.aestivum*_A : ------------------------------------------------------------------------------------------------------------------------------------------------------------------------- : -
*T.dicoccoides*_A : ------------------------------------------------------------------------------------------------------------------------------------------------------------------------- : -
*T.urartu*_A : ------------------------------------------------------------------------------------------------------------------------------------------------------------------------- : -
*T.aestivum*_D : GTTGCTGTGATGCTAACATTGAATACTGGATACATGTTGCATGCCAAAA-CTCAAACAAATCTACATCAATATTACTAT-CACTATTTCCCTGATGCAACACGCACCTGCTCTTATTATT-------GAGCACAGTTGTAATGTTGATTTCCAAAGCACATTTGTTACT : 2243
*Ae.tauschii*_D : GTTGCTGTGATGCTAACATTGAATACTGGATACATGTTGCATGCCAAAA-CTCAAACAAATCTACATCAATATTACTAT-CACTATTTCCCTGATGCAACACGCACCTGCTCTTATTATT-------GAGCACAGTTGTAATGTTGATTTCCAAAGCACATTTGTTACT : 2245
*T.aestivum*_B : GTTGCTGTGATGCTAACATTGAATACTGGATACCTGTTACATGCCAAAA-TTCAAACATATCTACATCAATATTATTATTCACTATTTCTCTGATGCAACACACACATGCTCTTATTATTATTCAATGAGCACGGTTGTAATGTTGACTTCCAAAGCACATTTGTTACT : 2248
*T.dicoccoides*_B : GTTGCTGTGATGCTAACATTGAATACTGGATACCTGTTACATGCCAAAAATTCAAACATATCTACATCAATATTATTATTCACTATTTCTCTGATGCAACACACACATGCTCTTATTATTATTCAATGAGCACGGTTGTAATGTTGACTTCCAAAGCACATTTGTTACT : 2249


 * 2380 * 2400 * 2420 * 2440 * 2460 * 2480 * 2500 * 2520 *
*T.aestivum*_A : ---------------TCAGTTCCAACTTTCTAGCGCTAAAATCCATATTATTTACTTTGTCTTGCAGGTCAGGGAAGACCCAGTTGGCTCATACTCTTTGTGTCTCCACTCAGGTCCATTTCCTGCCTTGTATATTCTCGGTGAAACCTCACTACATCAGAATCCATGA : 2270
*T.dicoccoides*_A : ---------------TCAGTTCCAACTTTCTAGCGCTAAAATCCATATTATTTACTTTGTCTTGCAGGTCAGGGAAGACCCAGTTGGCTCATACTCTTTGTGTCTCCACTCAGGTCCATTTCCTGCCTTGTATATTCTCGGTGAAACCTCACTACATCAGAATCCATGA : 2315
*T.urartu*_A : ---------------TCAGTTCCAACTTTCTAGCGCTAAAATCCATATTATTTACTTTGTCTTGCAGGTCAGGGAAGACCCAGTTGGCTCATACTCTTTGTGTCTCCACTCAGGTACATTTCCTGCCTTGTATATTCTCGGTGAAACCTCACTACATCAGAATCCATGA : 2226
*T.aestivum*_D : CAACCATTTC-----TCAATTCCAACTTTCTAGCGTTAAAATCCATATTATTTACTTTGTCTTGCAGGTCAGGGAAGACCCAGTTGGCTCATACTCTTTGTGTCTCCACTCAGGTCCATTTCCTGCCTTGTATTTTATCGATGAAACCTCACTAGAACAGAATCCATGA : 2407
*Ae.tauschii*_D : CAACCATTTC-----TCAATTCCAACTTTCTAGCGTTAAAATCCATATTATTTACTTTGTCTTGCAGGTCAGGGAAGACCCAGTTGGCTCATACTCTTTGTGTCTCCACTCAGGTCCATTTCCTGCCTTGTATTTTATCGATGAAACCTCACTAGAACAGAATCCATGA : 2409
*T.aestivum*_B : CAAACATTTCTCAATTCAGTTCCAACTTTCTAGCGCTAAAATCCATATTATTTACTTTGTTTTGCAGGTCAGGGAAGACCCAGTTGGCTCATACTCTTTGTGTCTCCACTCAGGTCCATTTCCTGCCTTGTAT-TTCTCGGTGAAACCTCACTACATCAGAATCCATGA : 2416
*T.dicoccoides*_B : CAAACATTTCTCAATTCAGTTCCAACTTTCTAGCGCTAAAATCCATATTATTTACTTTGTTTTGCAGGTCAGGGAAGACCCAGTTGGCTCATACTCTTTGTGTCTCCACTCAGGTCCATTTCCTGCCTTGTAT-TTCTCGGTGAAACCTCACTACATCAGAATCCATGA : 2417
 TCA TTCCAACTTTCTAGCG TAAAATCCATATTATTTACTTTGT TTGCAGGTCAGGGAAGACCCAGTTGGCTCATACTCTTTGTGTCTCCACTCAGGTcCATTTCCTGCCTTGTAT TT TCG TGAAACCTCACTA A CAGAATCCATGA

 2540 * 2560 * 2580 * 2600 * 2620 * 2640 * 2660 * 2680 * 2700
*T.aestivum*_A : ATAACTCTGCTTGTTTAATCAAA-------------------------------------------------------------------------------------------------------------------------------------------------- : 2293
*T.dicoccoides*_A : ATAACTCTGCTTGTTTAATCAAA-------------------------------------------------------------------------------------------------------------------------------------------------- : 2338
*T.urartu*_A : ATAACTCTGCTTGTTTAATCAAA-------------------------------------------------------------------------------------------------------------------------------------------------- : 2249
*T.aestivum*_D : ATAACTCTCCATGTTTAATCAAA-------------------------------------------------------------------------------------------------------------------------------------------------- : 2430
*Ae.tauschii*_D : ATAACTCTCCATGTTTAATCAAA-------------------------------------------------------------------------------------------------------------------------------------------------- : 2432
*T.aestivum*_B : ATAACTCTGCTTGTTTAATCAAAGAGTGAAGTGCACCCTAGGTCCTCGAACTATTTTGAAGGTGTCATATAGGTCCTCGAATTATGAAAAGTGTCATCCAGGTCCTCAAAAATCCTTGAAGTGCAATAAGTGTGTACGTATGTGACACATTTGAAATAGTTTAAGGACC : 2585
*T.dicoccoides*_B : ATAACTCTGCTTGTTTAATCAAA-------------------------------------------------------------------------------------------------------------------------------------------------- : 2440
 ATAACTCT C TGTTTAATCAAA

 * 2720 * 2740 * 2760 * 2780 * 2800 * 2820 * 2840 * 2860 *
*T.aestivum*_A : ----------------------------------------------------------------------------------------------------------------------------------------------------TGTATAACAGCTTCCACTCCA : 2314
*T.dicoccoides*_A : ----------------------------------------------------------------------------------------------------------------------------------------------------TGTATAACAGCTTCCACTCCA : 2359
*T.urartu*_A : ----------------------------------------------------------------------------------------------------------------------------------------------------TGTATAACAGCTTCCACTCCA : 2270
*T.aestivum*_D : ----------------------------------------------------------------------------------------------------------------------------------------------------TGTATAACAGCTTCCACTCCA : 2451
*Ae.tauschii*_D : ----------------------------------------------------------------------------------------------------------------------------------------------------TGTATAACAGCTTCCACTCCA : 2453
*T.aestivum*_B : TACATGACACCGCTAAAATAGTTCGAGGACTTGGATGACACGCATATTGCACTTTGAGGACCTGAATGACCCTTTTCATAGTTCGAGGACCTACGTAACACCTTTTAAATAGTTTGAGGACCTTTGATGCACTTCACTCTTAATCAAATGTATAACAGCTTCCACTCCA : 2754
*T.dicoccoides*_B : ----------------------------------------------------------------------------------------------------------------------------------------------------TGTATAACAGCTTCCACTCCA : 2461
 TGTATAACAGCTTCCACTCCA

 2880 * 2900 * 2920 * 2940 * 2960 * 2980 * 3000 * 3020 * 3040
*T.aestivum*_A : CATGCATGGTGGGAACGGGAAGGTTGCCTACATTGACACTGAGGGAACATTGTATCCTTTGAATTCCTTAGTAATACCTATAGTCGATTTGTTCAATGAATTCATTTACATTCCTGTGCTTATCGAAACTATTCCCTTAAAGGAGTTATCAGCCGGCCTGAACGCATTG : 2483
*T.dicoccoides*_A : CATGCATGGTGGGAACGGGAAGGTTGCCTACATTGACAGTGAGGGAACATTGTATCCTTTGAATTCCTTAGTAATACCTATAGTCGATTTGTTCAATGAATTCATTTACATTCCTGTGCTTATCGAAACTATTCCCTTAACGGAGTTATCAGCCGGCCTGAACGCATTG : 2528
*T.urartu*_A : CATGCATGGTGGGAACGGGAAGGTTGCCTACATTGACACTGAGGGAACATTGTATCCTTTGAATTCCTTAGTAATACCTATAGTCGATTTGTTCAATGAATTCATTTACATTCCTGTGCTTATCGAAACTATTCCCTTAACGGAGTTATCAGCCGGCCTGAACGCATTG : 2439
*T.aestivum*_D : CATGCATGGTGGGAACGGGAAGGTTGCCTACATTGACACTGAGGGAACATTGTATCCTTTGAATTCCTTAGTAATACCTATAGCTGATTTGTTCGATGAATTCATTTACATTCCTGTATTTCTCGAAACTGTTCCCTTAACGGAGTTATCAGCCGGCCTGAACGCATTG : 2620
*Ae.tauschii*_D : CATGCATGGTGGGAACGGGAAGGTTGCCTACATTGACACTGAGGGAACATTGTATCCTTTGAATTCCTTAGTAATACCTATAGCTGATTTGTTCGATGAATTCATTTACATTCCTGTATTTCTCGAAACTGTTCCCTTAACGGAGTTATCAGCCGGCCTGAACGCATTG : 2622
*T.aestivum*_B : TATGCATGGTGGGAACGGGAAGGTTGCCTACATTGGCACTGAGGGAACGTTGTATCCTTTGAATTCCTTAGTAATACCTAT-----------TTAATGAATTCATTTACATTCCTGTATTTATCGAAACTGTTCCCTTAACGGAGTTATCAGCCGGCCTGAACGCATTG : 2912
*T.dicoccoides*_B : TATGCATGGTGGGAACGGGAAGGTTGCCTACATTGACACTGAGGGAACGTTGTATCCTTTGAATTCCTTAGTAATACCTAT-----------TTAATGAATTCATTTACATTCCTGTATTTATCGAAACTGTTCCCTTAACGGAGTTATCAGCCGGCCTGAACGCATTG : 2619
 ATGCATGGTGGGAACGGGAAGGTTGCCTACATTGaCAcTGAGGGAAC TTGTATCCTTTGAATTCCTTAGTAATACCTAT T ATGAATTCATTTACATTCCTGT TT TCGAAACT TTCCCTTAAcGGAGTTATCAGCCGGCCTGAACGCATTG

 * 3060 * 3080 * 3100 * 3120 * 3140 * 3160 * 3180 * 3200 *
*T.aestivum*_A : TGCCAATTGCTGAGAGATTTGGGATGGATGCCAATGCTGTTCTTGACAATGTATGGCTCCTTTTACATCTCTCTTA--ACCCATTTAAGGAAGAATAGATCAAGATCTTTGTTTAATTCGTGATCTTTCTGTTTTAGATCATATACGCTCGCGCATACACCTATGAGCA : 2650
*T.dicoccoides*_A : TGCCAATTGCTGAGAGATTTGGGATGGATGCCAATGCTGTTCTTGACAATGTATGGCTCCTTTTACATCTCTCTTA--ACCCATTTAAGGAAGAATAGATCAAGATCTTTGTTTAATTCGTGATCTTTCTGTTTTAGATCATATACGCTCGCGCATACACCTATGAGCA : 2695
*T.urartu*_A : TGCCAATTGCTGAGAGATTTGGGATGGATGCCAATGCTGTTCTTGACAATGTATGGCTCCTTTTACATCTCTCTTA--ACCCATTTAAGGAAGAATAGATCAAGATCTTTGTTTAATTCGTGATCTTTCTGTTTTAGATCATATACGCTCGCGCATACACCTATGAGCA : 2606
*T.aestivum*_D : TGCCAATTGCTGAGAGATTTGGGATGGATGCCAATGCTGTTCTTGACAATGTATGGGTCCTTTTACATCTCTCTTATAACCCATTTAAGGAAGAATAGATCGAGATCTTTGTTTAATTTGTGATCTTTCTGTTTTAGATCATATATGCTCGTGCCTACACCTATGAGCA : 2789
*Ae.tauschii*_D : TGCCAATTGCTGAGAGATTTGGGATGGATGCCAATGCTGTTCTTGACAATGTATGGGTCCTTTTACATCTCTCTTATAACCCATTTAAGGAAGAATAGATCGAGATCTTTGTTTAATTTGTGATCTTTCTGTTTTAGATCATATATGCTCGTGCCTACACCTATGAGCA : 2791
*T.aestivum*_B : TGCCAATTGCTGAGAGATTTGGGATGGATGCCAATGCTGTTCTTGACAATGCATGGCTCCTTTTACATCTCTCTTA--ACCCATTTAAGGAAGAATAGATAAAGATCTTTGTTTAATTCGTGATCTTTCTGTTTTAGATCATATACGCTCGTGCATACACCTATGAGCA : 3079
*T.dicoccoides*_B : TGCCAATTGCTGAGAGATTTGGGATGGATGCCAATGCTGTTCTTGACAATGCATGGCTCCTTTTACATCTCTCTTA--ACCCATTTAAGGAAGAATAGATAAAGATCTTTGTTTAATTCGTGATCTTTCTGTTTTAGATCATATACGCTCGTGCATACACCTATGAGCA : 2786
 TGCCAATTGCTGAGAGATTTGGGATGGATGCCAATGCTGTTCTTGACAATG ATGG TCCTTTTACATCTCTCTTA ACCCATTTAAGGAAGAATAGAT AGATCTTTGTTTAATT GTGATCTTTCTGTTTTAGATCATATA GCTCG GC TACACCTATGAGCA

 3220 * 3240 * 3260 * 3280 * 3300 * 3320 * 3340 * 3360 * 3380
*T.aestivum*_A : CCAGTACAACTTACTCCTGGGCCTTGCTGCCAAGATGGCCGAAGAGCCTTTCAGGCTTCTGGTACGCATGACTTTGCTGACATGTACTATCAAACTTACAAGTTGATAGATCTCAACTGTGCTCATGTGATCTTTGTTTGGCTTGGAAATGATAGATCGTGGATTCTGT : 2819
*T.dicoccoides*_A : CCAGTACAACTTACTCCTGGGCCTTGCTGCCAAGATGGCCGAAGAGCCTTTCAGGCTTCTGGTACGCATGACTTTGCTGACATGTACTATCAAACTTACAAGTTGATAGATCTCAACTGTGCTCATGTGATCTTTGTTTGGCTTGGAAATGATAGATCGTGGATTCTGT : 2864
*T.urartu*_A : CCAGTACAACTTACTCCTGGGCCTTGCTGCCAAGATGGCCGAAGAGCCTTTCAGGCTTCTGGTACGCATGACTTTGCTGACATGTACTATCAAACTTACAAGTTGATAGATCTCAACTGTGCTCATGTGATCTTTGTTTGGCTTGGAAATGATAGATCGTGGATTCTGT : 2775
*T.aestivum*_D : CCAGTACAACTTACTCCTGGGCCTTGCTGCCAAGATGGCTGAAGAGCCTTTCAGGCTTCTGGTACGCATGACTTTGCTGCCATGTA------AATTTACAAGTTGATAGATCTCAACTGTGCTCATGTGTTCTTTGTTTGACTTGGAAATGATAGATCGTGGATTCTGT : 2952
*Ae.tauschii*_D : CCAGTACAACTTACTCCTGGGCCTTGCTGCCAAGATGGCTGAAGAGCCTTTCAGGCTTCTGGTACGCATGACTTTGCTGCCATGTA------AATTTACAAGTTGATAGATCTCAACTGTGCTCATGTGTTCTTTGTTTGACTTGGAAATGATAGATCGTGGATTCTGT : 2954
*T.aestivum*_B : CCAGTACAACTTACTCCTGGGCCTTGTTGCCAAGATGGCTGAAGAGCCTTTCAGGCTTCTGGTACGCATGACTTTGCTGACATGTAGTGTTAAACTTACAAGTTGATAGATCTCAACTGTGCTCATGTAATCTTTGTTTGGCTTGGAAATGATAGATCGTGGATTCTGT : 3248
*T.dicoccoides*_B : CCAGTACAACTTACTCCTGGGCCTTGCTGCCAAGATGGCTGAAGAGCCTTTCAGGCTTCTGGTACGCATGACTTTGCTGACATGTAGTATTAAACTTACAAGTTGATAGATCTCAACTGTGCTCATGTGATCTTTGTTTGGCTTGGAAATGATAGATCGTGGATTCTGT : 2955
 CCAGTACAACTTACTCCTGGGCCTTGcTGCCAAGATGGC GAAGAGCCTTTCAGGCTTCTGGTACGCATGACTTTGCTG CATGTA AA TTACAAGTTGATAGATCTCAACTGTGCTCATGTg TCTTTGTTTG CTTGGAAATGATAGATCGTGGATTCTGT

 * 3400 * 3420 * 3440 * 3460 * 3480 * 3500 * 3520 * 3540
*T.aestivum*_A : GATTGCGCTATTCCGTGTTGATTTCAGTGGTAGGGGTGAACTTGCAGAGCGTCAGGTATTCTACTGTAACTAGCTAACTATGTGAAAAAATCAAGCAACTCATGATGTAGTCGAATGCT---TGCATTTTATACACTTGCTCTAAGTGATGTGCTCTGGAACTGCAGCA : 2985
*T.dicoccoides*_A : GATTGCGCTATTCCGTGTTGATTTCAGTGGTAGGGGTGAACTTGCAGAGCGTCAGGTATTCTACTGTAACTAGCTAACTATGTGAAAAAATCAAGCAACTCATGATGTAGTCGAATGCT---TGCATTTTATACACTTGCTCTAAGTGATGTGCTCTGGAACTGCAGCA : 3030
*T.urartu*_A : GATTGCGCTATTCCGTGTTGATTTCAGTGGTAGGGGTGAACTTGCAGAGCGTCAGGTATTCTACTGTAACTAGCTAACTATGTGAAAAAATCAAGCAACTCATGATGTAGTCGAATGCT---TGCATTTTATACACTTGCTCTAAGTGATGTGCTCTGGAACTGCAGCA : 2941
*T.aestivum*_D : GATTGCGCTGTTCCGTGTTGATTTCAGTGGTAGGGGTGAACTTGCAGAGCGTCAGGTATTCTACTGTAACTAGCTAACTACATGAAAAAATCAAGCAACTCATGAAGTAGTCGAATGCTGCTTGCATTTTATACACTTGCTTTAAGTGATGTGCTCTGGAACTGCAGCA : 3121
*Ae.tauschii*_D : GATTGCGCTGTTCCGTGTTGATTTCAGTGGTAGGGGTGAACTTGCAGAGCGTCAGGTATTCTACTGTAACTAGCTAACTACATGAAAAAATCAAGCAACTCATGAAGTAGTCGAATGCTGCTTGCATTTTATACACTTGCTTTAAGTGATGTGCTCTGGAACTGCAGCA : 3123
*T.aestivum*_B : GATTGCGCTATTCCGTGTTGATTTCAGTGGCAGGGGTGAACTTGCAGAGCGTCAGGTATTCTACTGTAACTAGCTAACTATGTGAAAAAATCAAGCAACTCATGATGTAGTCGAATGCT---TGCATTTTATACACTTGCTCTAAGTGATGTGCTCTGGAACTGCAGCA : 3414
*T.dicoccoides*_B : GATTGCGCTATTCCGTGTTGATTTCAGTGGTAGGGGTGAACTTGCAGAGCGTCAGGTATTCTACTGTAACTAGCTAACTATGTGAAAAAATCAAGCAACTCATGATGTAGTCGAATGCT---TGCATTTTATACACTTGCTCTAAGTGATGTGCTCTGGAAATGCAGCA : 3121
 GATTGCGCT TTCCGTGTTGATTTCAGTGGtAGGGGTGAACTTGCAGAGCGTCAGGTATTCTACTGTAACTAGCTAACTA TGAAAAAATCAAGCAACTCATGA GTAGTCGAATGCT TGCATTTTATACACTTGCT TAAGTGATGTGCTCTGGAAcTGCAGCA

 * 3560 * 3580 * 3600 * 3620 * 3640 * 3660 * 3680 * 3700 * 37
*T.aestivum*_A : AAAACTGGCACAAATGCTGTCCCGCCTTACAAAGATTGCTGAGGAGTTCAATGTTGCAGTGTACATCACCAACCAAGGTGTGCTTT-CCAATCTATCCTGTCTGTTCCAAGAAAGAGCTCCTTATATTCGTGGATCTCAAATCATATAAGTTCTTTCCTTGTTCCAGTG : 3153
*T.dicoccoides*_A : AAAACTGGCACAAATGCTGTCCCGCCTTACAAAGATTGCTGAGGAGTTCAATGTTGCAGTGTACATCACCAACCAAGGTGTGCTTT-CCAATCTATCCTGTCTGTTCCAAGAAAGAGCTCCTTATATTCGTGGATCTCAAATCATATAAGTTCTTTCCTTGTTCCAGTG : 3198
*T.urartu*_A : AAAACTGGCACAAATGCTGTCCCGCCTTACAAAGATTGCTGAGGAGTTCAATGTTGCAGTGTACATCACCAACCAAGGTGTGCTTT-CCAATCTATCCTGTCTGTTCCAAGAAAGAGCTCCTTATATTCGTGGATCTCAAATCATATAAGTTCTTTCCTTGTTCCAGTG : 3109
*T.aestivum*_D : AAAGCTGGCACAAATGCTGTCCCGCCTTACAAAGATTGCTGAGGAGTTCAATGTTGCAGTGTACATCACCAACCAAGGTGTGCTTT-CCAATCTATCCTGTCTGTTCCAAGAAAGAGCTCCTTATATTCGTGGATCTCAAATCATATAAGTTCTTTCCTTGTTCCAGTG : 3289
*Ae.tauschii*_D : AAAGCTGGCACAAATGCTGTCCCGCCTTACAAAGATTGCTGAGGAGTTCAATGTTGCAGTGTACATCACCAACCAAGGTGTGCTTT-CCAATCTATCCTGTCTGTTCCAAGAAAGAGCTCCTTATATTCGTGGATCTCAAATCATATAAGTTCTTTCCTTGTTCCAGTG : 3291
*T.aestivum*_B : AAAACTGGCACAAATGCTTTCCCGCCTTACAAAGATTGCTGAGGAGTTCAATGTTGCAGTGTACATCACCAACCAAGGTGTGCTTTTCCAATCTATCCTGTCTGTTCCAAGAAAGAGCTCCTTATATTCGTGGATCTCAAATCATACAAGTTCTTTCCTTGTTCCAGTG : 3583
*T.dicoccoides*_B : AAAACTGGCACAAATGCTTTCCCGCCTTACAAAGATTGCTGAGGAGTTCAATGTTGCAGTGTACATCACCAACCAAGGTGTGCTTTTCCAATCTATCCTGTCTGTTCCAAGAAAGAGCTCCTTATATTCGTGGATCTCAAATCATACAAGTTCTTTCCTTGTTCCAGTG : 3290
 AAA CTGGCACAAATGCT TCCCGCCTTACAAAGATTGCTGAGGAGTTCAATGTTGCAGTGTACATCACCAACCAAGGTGTGCTTT CCAATCTATCCTGTCTGTTCCAAGAAAGAGCTCCTTATATTCGTGGATCTCAAATCATA AAGTTCTTTCCTTGTTCCAGTG

 20 * 3740 * 3760 * 3780 * 3800 * 3820 * 3840 * 3860 * 3880
*T.aestivum*_A : ATTGCGGACCCAGGTGGTGGTATGTTCATCACTGACCCCAAAAAGCCGGCAGGAGGCCACGTGCTGGCGCATGCAGCCACCATCCGGTTGATGCTGAGGAAAAGCAAAGGCGAGCAGCGTGTCTGCAAGATCTTTGACGCCCCTAACCTTCCCGAGGGAGAAGCTATAT : 3322
*T.dicoccoides*_A : ATTGCGGACCCAGGTGGTGGTATGTTCATCACTGACCCCAAAAAGCCGGCAGGAGGCCACGTGCTGGCGCATGCAACCACCATCCGGTTGATGCTGAGGAAAAGCAAAGGCGAGCAGCGTGTCTGCAAGATCTTTGACGCCCCTAACCTTCCCGAGGGAGAAGCTATAT : 3367
*T.urartu*_A : ATTGCGGACCCAGGTGGTGGTATGTTCATCACTGACCCCAAAAAGCCGGCAGGAGGCCACGTGCTGGCGCATGCAGCCACCATCCGGTTGATGCTGAGGAAAAGCAAAGGCGAGCAGCGTGTCTGCAAGATCTTTGACGCCCCTAACCTTCCCGAGGGAGAAGCTATAT : 3278
*T.aestivum*_D : ATTGCGGACCCAGGTGGTGGTATGTTCATCACTGACCCCAAAAAGCCGGCGGGAGGCCACGTGCTGGCGCATGCAGCCACCATCCGGTTGATGCTGAGGAAAGGCAAAGGCGAGCAGCGTGTCTGCAAGATCTTTGACGCCCCTAACCTTCCCGAGGGAGAAGCTATAT : 3458
*Ae.tauschii*_D : ATTGCGGACCCAGGTGGTGGTATGTTCATCACTGACCCCAAAAAGCCGGCGGGAGGCCACGTGCTGGCGCATGCAGCCACCATCCGGTTGATGCTGAGGAAAGGCAAAGGCGAGCAGCGTGTCTGCAAGATCTTTGACGCCCCTAACCTTCCCGAGGGAGAAGCTATAT : 3460
*T.aestivum*_B : ATTGCGGACCCAGGTGGTGGTATGTTCATCACTGACCCCAAAAAGCCGGCGGGAGGCCACGTGCTGGCGCATGCAGCCACCATCCGGTTGATGCTGAGGAAAGGCAAAGGCGAGCAGCGTATCTGCAAGATCTTTGACGCCCCTAACCTTCCCGAGGGAGAAGCTATAT : 3752
*T.dicoccoides*_B : ATTGCGGACCCAGGTGGTGGTATGTTCATCACTGACCCCAAAAAGCCGGCGGGAGGCCACGTGCTGGCGCATGCAGCCACCATCCGGTTGATGCTGAGGAAAGGCAAAGGCGAGCAGCGTGTCTGCAAGATCTTTGACGCCCCTAACCTTCCCGAGGGAGAAGCTATAT : 3459
 ATTGCGGACCCAGGTGGTGGTATGTTCATCACTGACCCCAAAAAGCCGGC GGAGGCCACGTGCTGGCGCATGCAgCCACCATCCGGTTGATGCTGAGGAAA GCAAAGGCGAGCAGCGTgTCTGCAAGATCTTTGACGCCCCTAACCTTCCCGAGGGAGAAGCTATAT

 * 3900 * 3920 * 3940 * 3960 * 3980 * 4000 * 4020 * 4040 *
*T.aestivum*_A : CCTTTTGCTTACTA---------CTTGTTTACTGCTTGTGC--TATTCATTGCTTTAATTTGTTTGGTTGCTGAACTCTTGATAGGATTGTTTTGCTCAGACTTGCACAGC----TTTATGATTTAGTTCTTAGTTGTTAACCTCCTTTGCTGAAGCTTCACGTAGCAC : 3476
*T.dicoccoides*_A : CCTTTTGCTTACTA---------CTTGTTTACTGCTTGTGC--TATTCATTGCTTTAATTTGTTTGGTTGCTGAACTCTTGATAGGATTGTTTTGCTCAGACTTGCACAGC----TTTATGATTTAGTTCTTAGTTGTTAACCTCCTTTACTGAAGCTTCACGTAGCAC : 3521
*T.urartu*_A : CCTTTTGCTTACTAGTA------CTTGTTTACTGCTTGTGC--TATTCATTGCTTTAATTTGTTTGGTTGCTGAACTCTTGATAGGATTGTTTTGCTCAGACTTGCACAGC----TTTATGATTTAGTTCTTAGTTGTTAACCTCCTTTACTGAAGCTTCACGTAGCAC : 3435
*T.aestivum*_D : CCTTTTGCTCTATACTA------CTTGTTTACTGCTTGTGCGCTATTCATTGCTTTAATTTGTTTGGTTGTTGAACTCTTGATAGGATTGTTTTGCTCAGACTTGCACAGCAAAGTTTATGATTTAGTTCT-AGTTGTTAACCTCCTTTACTGAAGCTTCACGTAGCAC : 3620
*Ae.tauschii*_D : CCTTTTGCTCTATACTA------CTTGTTTACTGCTTGTGCGCTATTCATTGCTTTAATTTGTTTGGTTGTTGAACTCTTGATAGGATTGTTTTG-TCAGACTTGCACAGCAAAGTTTATGATTTAGTTCT-AGTTGTTAACCTCCTTTACTGAAGCTTCACGTAGCAC : 3621
*T.aestivum*_B : CCTTTTGCTCTATACTAGGAGTACTTGTTTACTGCTTGTGC--TATTCATTCCATTAATTTGTTTGGTTGCTGAACTCTTGATAGGATTGTTTTGCTCAGACTTGCGCAGCAAAGTTTATGATTTAGTTCT-------------------------------------- : 3881
*T.dicoccoides*_B : CCTTTTGCTCTATACTAGGAGTACTTGTTTACTGCTTGTGC--TATTCATTCCATTAATTTGTTTGGTTGCTGAACTCTTGATAGGATTGTTTTGCTCAGACTTGCGCAGCAAAGTTTATGATTTAGTTCT-------------------------------------- : 3588
 CCTTTTGCT TA CTTGTTTACTGCTTGTGC TATTCATT C TTAATTTGTTTGGTTG TGAACTCTTGATAGGATTGTTTTGcTCAGACTTGC CAGC TTTATGATTTAGTTCT

 4060 * 4080 * 4100 * 4120 * 4140 *
*T.aestivum*_A : AAATTAGCAATGATCAGATGTCGAGAAATTGTTTCCCTTGACCACCAACACGTTTTCCAGATTACAACAGGCGGATTGATGGATGTGAAAGACTGA : 3572
*T.dicoccoides*_A : AAATTAGCAATGATCAGATGTCGAGAAATTGTTTCCCTTGACCACCAACACGTTTTCCAGATTACAACAGGCGGATTGATGGATGTGAAAGACTGA : 3617
*T.urartu*_A : AAATTAGCAATGATCAGATGTCGAGAAATTGTTTCCCTTGACCACCAACACGTTTTCCAGATTACAACAGGCGGATTGATGGATGTGAAAGACTGA : 3531
*T.aestivum*_D : AAATTAGCGACGATCAGATGTCGAGAAATTGTTTCCCTTGACCACCAACACGTTTTCCAGATCACAACAGGCGGATTGATGGATGTGAAAGACTGA : 3716
*Ae.tauschii*_D : AAATTAGCGACGATCAGATGTCGAGAAATTGTTTCCCTTGACCACCAACACGTTTTCCAGATCACAACAGGCGGATTGATGGATGTGAAAGACTGA : 3717
*T.aestivum*_B : ---------------AGTTGTTGAGAAATTGTTTCGCTTGACCACCAACACGTTTTCCAGATTACAACAGGTGGATTGATGGATGTGAAAGACTGA : 3962
*T.dicoccoides*_B : ---------------AGTTGTTGAGAAATTGTTTCGCTTGACCACCAACACGTTTTCCAGATTACAACAGGTGGATTGATGGATGTGAAAGACTGA : 3669
 AG TGT GAGAAATTGTTTC CTTGACCACCAACACGTTTTCCAGAT ACAACAGG GGATTGATGGATGTGAAAGACTGA
