# Supplementary material for: Dmc1 is a candidate for temperature tolerance during wheat meiosis
Source: Theor Appl Genet. 2019 Dec 18;133(3):809–28. doi: 10.1007/s00122-019-03508-9 (PMC7021665; doi:10.1007/s00122-019-03508-9)

***T. aestivum A copy* (TraesCS5A02G133000)**


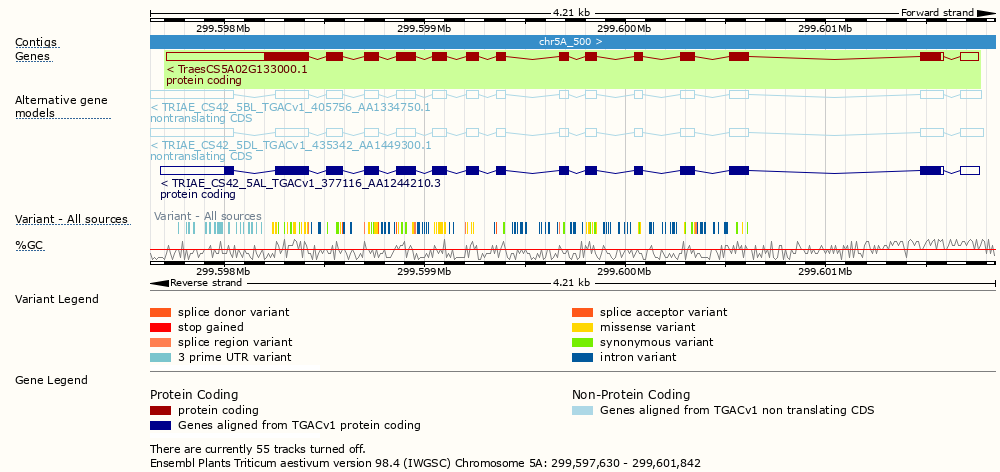


***T. urartu* *A copy* (TRIUR3_13472)**


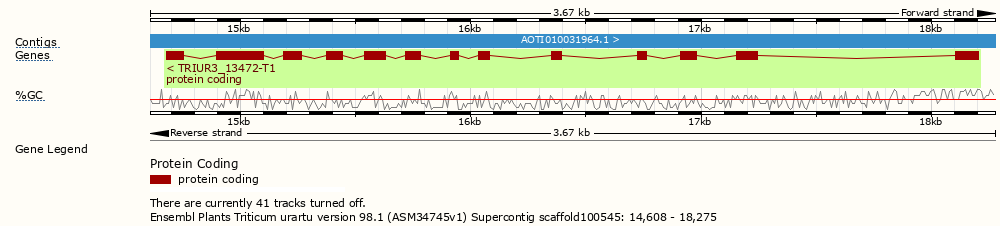


***T. dicoccoides* *A copy* (TRIDC5AG022500)**


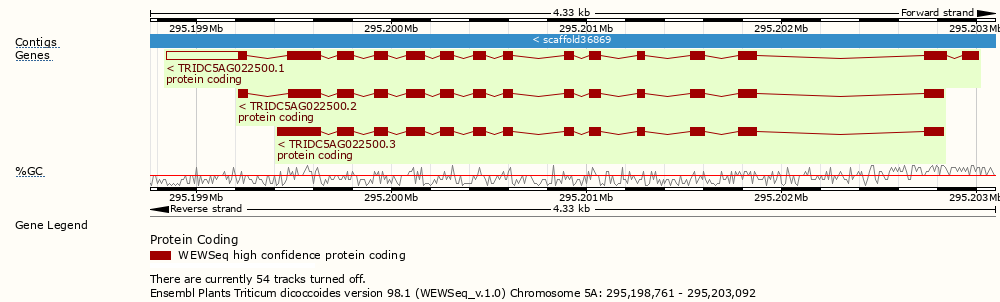


***T. aestivum* *B copy* (TraesCS5B02G131900)**


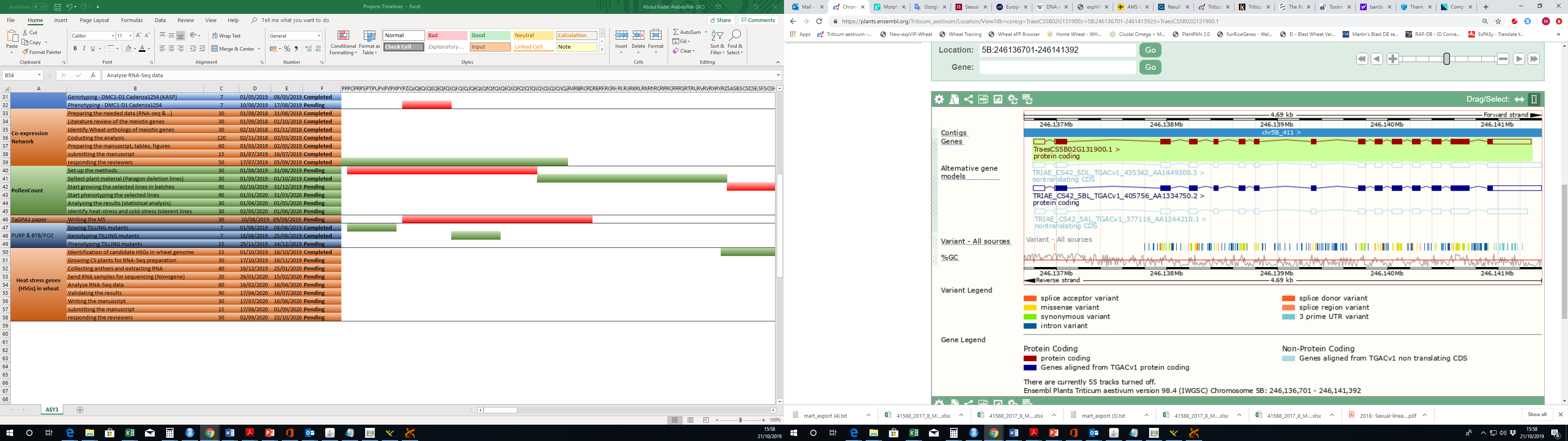


***T. dicoccoides* *B copy* (TRIDC5BG023380)**


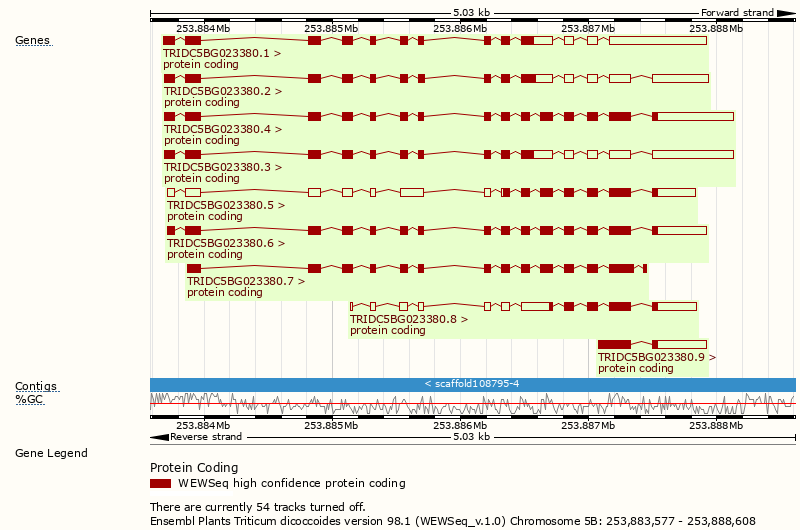


***T. aestivum* *D copy* (TraesCS5D02G141200)**


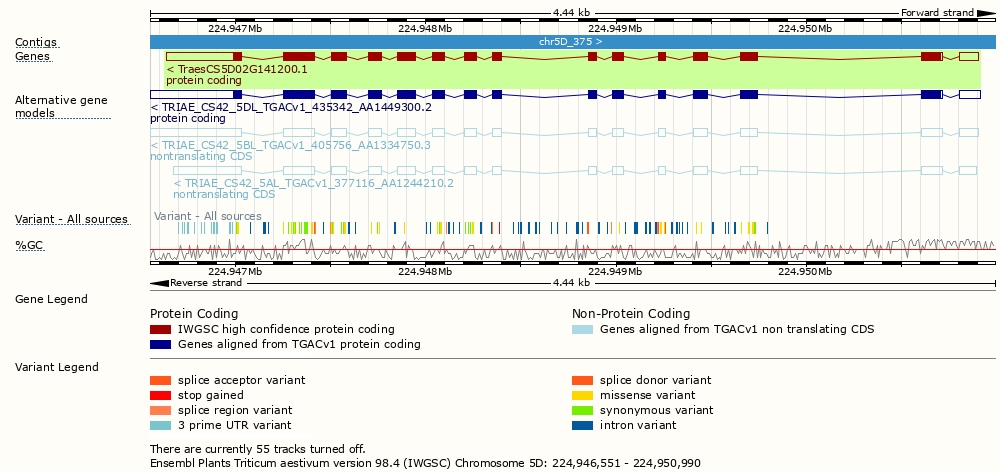


***Ae. tauschii* *D copy* (AET5Gv20357200)**


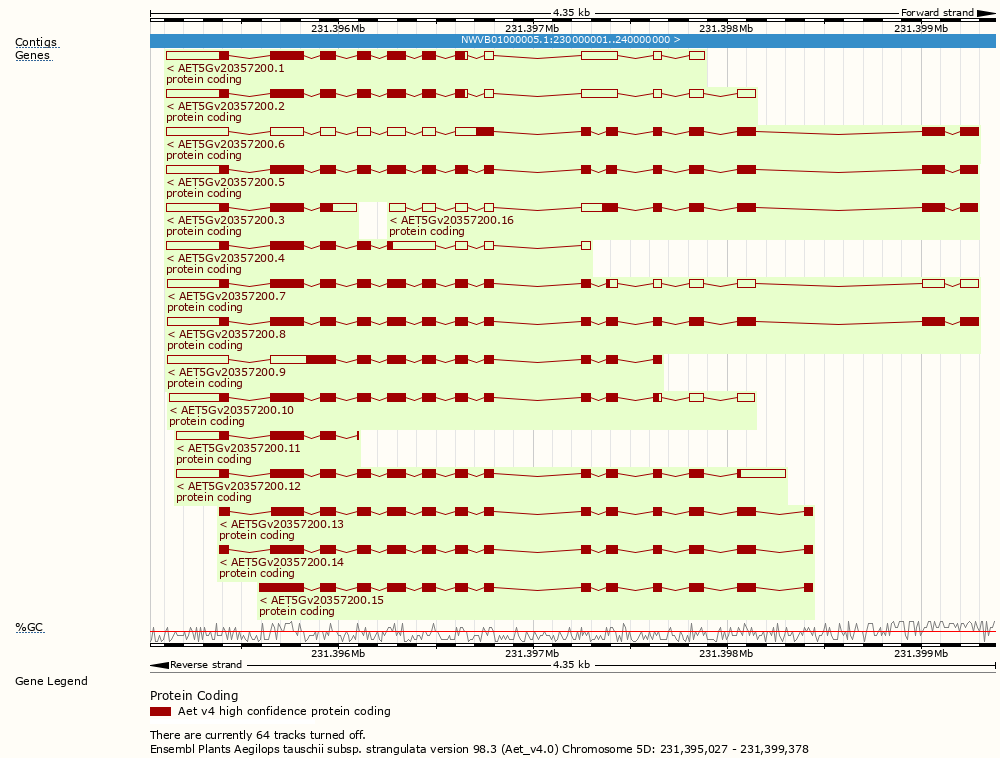

Supplement: Supplementary file 6 — DMC1 transcripts in T. aestivum and its diploid and tetraploid ancestors (as predicted in Ensembl Plants) (DOCX 982 kb) [file 122_2019_3508_MOESM6_ESM.docx]
