# Supplementary material for: Dmc1 is a candidate for temperature tolerance during wheat meiosis
Source: Theor Appl Genet. 2019 Dec 18;133(3):809–28. doi: 10.1007/s00122-019-03508-9 (PMC7021665; doi:10.1007/s00122-019-03508-9)
Supplement: Supplementary file 7 — Multiple alignment of DMC1 amino-acid sequences from T. aestivum (Ta) and its diploid and tetraploid ancestors: the A-genome donor, T. urartu (Tu_A), the D-genome donor, Ae. tauschii (Aet_D) and the AABB genome progenitor, T. dicoccoides (Td_A and Td_B). Ae tauschii and T. dicoccoides have multiple transcripts of their Dmc1 genes due to alternative splicing variants, but this alignment only includes transcripts that are most similar to those of T. aestivum. Numbers after the dots refer to the transcript number. Large red triangle indicates the single amino-acid substitution in TaDMC1-D1 that may confer low temperature tolerance in wheat. Note the conservation of this substitution in the ancestral D-genome of Ae. tauschii. Small blue triangles show the positions of three other amino-acid substitutions in TaDMC1-B1, and a small green triangle shows the position of a substitution in TaDMC1-A1 that is also conserved in the ancestral copies (RTF 101 kb) [file 122_2019_3508_MOESM7_ESM.rtf]

                                                                                                                                                                      
                   *        20         *        40         *        60         *        80         *       100         *       120         *       140         *      
Td_B.4  : -----ASQTSPLPQPWPHFLSSSSTRSSASY-RRARGMAPSKQYDEGGQLQLMEADRVEEEEECFESIDKLISQGINSGDVKKLQDAGIYTCNGLMMHTKKSLTGIKGLSEAKVDKICEAAEKLLSQGFMTGSDLLIKRKSVVRITTGSQ : 144
Td_B.6  : --------------PWPHFLSSSSTRSSASYSRRARGMAPSKQYDEGGQLQLMEADRVEEEEECFESIDKLISQGINSGDVKKLQDAGIYTCNGLMMHTKKSLTGIKGLSEAKVDKICEAAEKLLSQGFMTGSDLLIKRKSVVRITTGSQ : 136
Ta_B.1  : -------------------------------------MAPSKQYDEGGQLQLMEADRVEEEEECFESIDKLISQGINSGDVKKLQDAGIYTCNGLMMHTKKSLTGIKGLSEAKVDKICEAAEKLLSQGFMTGSDLLIKRKSVVRITTGSQ : 113
Aet_D.8 : STRTCASQTSPLPQPPPHF-SSSSTRTSASY-RRTRGMAPSKQYDEGGQLQLMEADRVEEEEECFESIDKLISQGINSGDVKKLQDAGIYTCNGLMMHTKKSLTGIKGLSEAKVDKICEAAEKLLSQGFMTGSDLLIKRKSVVRITTGSQ : 148
Aet_D.5 : --RTCASQTSPLPQPPPHF-SSSSTRTSASY-RRTRGMAPSKQYDEGGQLQLMEADRVEEEEECFESIDKLISQGINSGDVKKLQDAGIYTCNGLMMHTKKSLTGIKGLSEAKVDKICEAAEKLLSQGFMTGSDLLIK--SVVRITTGSQ : 144
Ta_D.1  : -------------------------------------MAPSKQYDEGGQLQLMEADRVEEEEECFESIDKLISQGINSGDVKKLQDAGIYTCNGLMMHTKKSLTGIKGLSEAKVDKICEAAEKLLSQGFMTGSDLLIKRKSVVRITTGSQ : 113
Ta_A.1  : -------------------------------------MAPSKQYDEGGQLQLMEADRVEEEEECFESIDKLISQGINSGDVKKLQDAGIYTCNGLMMHTKKSLTGIKGLSEAKVDKICEAAEKLLSQGFMTGSDLLIKRKSVVRITTGSQ : 113
Td_A.2  : -------------------------------------MAPSKQYDEGGQLQLMEADRVEEEEECFESIDKLISQGINSGDVKKLQDAGIYTCNGLMMHTKKSLTGIKGLSEAKVDKICEAAEKLLSQGFMTGSDLLIKRKSVVRITTGSQ : 113
Td_A.1  : -----LPKPPLSPSLRPTSFSSSSTRSSGSY-RRARGMAPSKQYDEGGQLQLMEADRVEEEEECFESIDKLISQGINSGDVKKLQDAGIYTCNGLMMHTKKSLTGIKGLSEAKVDKICEAAEKLLSQGFMTGSDLLIKRKSVVRITTGSQ : 144
Tu_A.1  : -------------------------------------MAPSKQYDEGGQLQLMEADRVEEEEECFESIDKLISQGINSGDVKKLQDAGIYTCNGLMMHTKKSLTGIKGLSEAKVDKICEAAEKLLSQGFMTGSDLLIK--EGLKHSVSQR : 111
                                               MAPSKQYDEGGQLQLMEADRVEEEEECFESIDKLISQGINSGDVKKLQDAGIYTCNGLMMHTKKSLTGIKGLSEAKVDKICEAAEKLLSQGFMTGSDLLIKrksv64i3tgsq      
                                                                                                                                                                      
                 160         *       180         *       200         *       220         *       240         *       260         *       280         *       300      
Td_B.4  : ALDELLGGGIETLCITEAFGEFRSGKTQLAHTLCVSTQLPLHMHGGNGKVAYIDTEGTFRPERIVPIAERFGMDANAVLDNIIYARAYTYEHQYNLLLGLAAKMAEEPFRLLIVDSVIALFRVDFSGRGELAERQQKLAQMLSRLTKIAE : 294
Td_B.6  : ALDELLGGGIETLCITEAFGEFRSGKTQLAHTLCVSTQLPLHMHGGNGKVAYIDTEGTFRPERIVPIAERFGMDANAVLDNIIYARAYTYEHQYNLLLGLAAKMAEEPFRLLIVDSVIALFRVDFSGRGELAERQQKLAQMLSRLTKIAE : 286
Ta_B.1  : ALDELLGGGIETLCITEAFGEFRSGKTQLAHTLCVSTQLPLHMHGGNGKVAYIGTEGTFRPERIVPIAERFGMDANAVLDNIIYARAYTYEHQYNLLLGLVAKMAEEPFRLLIVDSVIALFRVDFSGRGELAERQQKLAQMLSRLTKIAE : 263
Aet_D.8 : TLDELLGGGIETLCITEAFGEFRSGKTQLAHTLCVSTQLPLHMHGGNGKVAYIDTEGTFRPERIVPIAERFGMDANAVLDNIIYARAYTYEHQYNLLLGLAAKMAEEPFRLLIVDSVIALFRVDFSGRGELAERQQKLAQMLSRLTKIAE : 298
Aet_D.5 : TLDELLGGGIETLCITEAFGEFRSGKTQLAHTLCVSTQLPLHMHGGNGKVAYIDTEGTFRPERIVPIAERFGMDANAVLDNIIYARAYTYEHQYNLLLGLAAKMAEEPFRLLIVDSVIALFRVDFSGRGELAERQQKLAQMLSRLTKIAE : 294
Ta_D.1  : TLDELLGGGIETLCITEAFGEFRSGKTQLAHTLCVSTQLPLHMHGGNGKVAYIDTEGTFRPERIVPIAERFGMDANAVLDNIIYARAYTYEHQYNLLLGLAAKMAEEPFRLLIVDSVIALFRVDFSGRGELAERQQKLAQMLSRLTKIAE : 263
Ta_A.1  : ALDELLGGGIETLCITEAFGEFRSGKTQLAHTLCVSTQLPLHMHGGNGKVAYIDTEGTFRPERIVPIAERFGMDANAVLDNIIYARAYTYEHQYNLLLGLAAKMAEEPFRLLIVDSVIALFRVDFSGRGELAERQQKLAQMLSRLTKIAE : 263
Td_A.2  : ALDELLGGGIETLCITEAFGEFRSGKTQLAHTLCVSTQLPLHMHGGNGKVAYIDSEGTFRPERIVPIAERFGMDANAVLDNIIYARAYTYEHQYNLLLGLAAKMAEEPFRLLIVDSVIALFRVDFSGRGELAERQQKLAQMLSRLTKIAE : 263
Td_A.1  : ALDELLGGGIETLCITEAFGEFRSGKTQLAHTLCVSTQLPLHMHGGNGKVAYIDSEGTFRPERIVPIAERFGMDANAVLDNIIYARAYTYEHQYNLLLGLAAKMAEEPFRLLIVDSVIALFRVDFSGRGELAERQQKLAQMLSRLTKIAE : 294
Tu_A.1  : HLES-------------------SGQGRPSWLILFVSPLSFHST------CMVGTGS--RPERIVPIAERFGMDANAVLDNIIYARAYTYEHQYNLLLGLAAKMAEEPFRLLIVDSVIALFRVDFSGRGELAERQQKLAQMLSRLTKIAE : 234
           LdellgggietlciteafgefrSGktqlaht6cvs3qLplHmhggngkvay6d3egtfRPERIVPIAERFGMDANAVLDNIIYARAYTYEHQYNLLLGLaAKMAEEPFRLLIVDSVIALFRVDFSGRGELAERQQKLAQMLSRLTKIAE      
                                                                                                                        
                   *       320         *       340         *       360         *       380         *       400          
Td_B.4  : EFNVAVYITNQVIADPGGGMFITDPKKPAGGHVLAHAATIRLMLRKGKGEQRVCKIFDAPNLPEGEAV-----------------------FQITTGGLMDVKD : 375
Td_B.6  : EFNVAVYITNQVIADPGGGMFITDPKKPAGGHVLAHAATIRLMLRKGKGEQRVCKIFDAPNLPEGEAV-----------------------FQITTGGLMDVKD : 367
Ta_B.1  : EFNVAVYITNQVIADPGGGMFITDPKKPAGGHVLAHAATIRLMLRKGKGEQRICKIFDAPNLPEGEAV-----------------------FQITTGGLMDVKD : 344
Aet_D.8 : EFNVAVYITNQVIADPGGGMFITDPKKPAGGHVLAHAATIRLMLRKGKGEQRVCKIFDAPNLPEGEAV-----------------------FQITTGGLMDVKD : 379
Aet_D.5 : EFNVAVYITNQVIADPGGGMFITDPKKPAGGHVLAHAATIRLMLRKGKGEQRVCKIFDAPNLPEGEAV-----------------------FQITTGGLMDVKD : 375
Ta_D.1  : EFNVAVYITNQVIADPGGGMFITDPKKPAGGHVLAHAATIRLMLRKGKGEQRVCKIFDAPNLPEGEAV-----------------------FQITTGGLMDVKD : 344
Ta_A.1  : EFNVAVYITNQVIADPGGGMFITDPKKPAGGHVLAHAATIRLMLRKSKGEQRVCKIFDAPNLPEGEAV-----------------------FQITTGGLMDVKD : 344
Td_A.2  : EFNVAVYITNQVIADPGGGMFITDPKKPAGGHVLAHATTIRLMLRKSKGEQRVCKIFDAPNLPEGEHV-----------------------FQITTGGLMDVKD : 344
Td_A.1  : EFNVAVYITNQVIADPGGGMFITDPKKPAGGHVLAHATTIRLMLRKSKGEQRVCKIFDAPNLPEGEAV-----------------------FQITTGGLMDVKD : 375
Tu_A.1  : EFNVAVYITNQVIADPGGGMFITDPKKPAGGHVLAHAATIRLMLRKSKGEQRVCKIFDAPNLPEGEAISFCLLVLVYCLCREIVSLDHQHVFQITTGGLMDVKD : 338
          EFNVAVYITNQVIADPGGGMFITDPKKPAGGHVLAHAaTIRLMLRK KGEQR6CKIFDAPNLPEGEa6                       FQITTGGLMDVKD      
