# Supplementary material for: Dmc1 is a candidate for temperature tolerance during wheat meiosis
Source: Theor Appl Genet. 2019 Dec 18;133(3):809–28. doi: 10.1007/s00122-019-03508-9 (PMC7021665; doi:10.1007/s00122-019-03508-9)
Supplement: Supplementary file 8 — The effects of three different temperature treatments on meiotic metaphase I chromosomes of individual Chinese Spring (CS) wild-type and 22-F5 (ttmei1) mutant plants. The mean numbers of univalents, ring and rod bivalents, trivalents, tetravalents and pentavalents were scored along with chiasma frequency scored as single or double crossovers. Standard deviation (SD) and standard error (SE) are shown, as well as the maximum (max) and minimum (min) number of chromosomes with a particular conformation (PDF 675 kb) [file 122_2019_3508_MOESM8_ESM.pdf]

Plant	Temp.		Univalents	Bivalent (rod)	Bivalent (ring)	Trivalent	Tetravalent	Pentavalent	Chiasma frequency (Single crossovers)	Chiasma frequency (Double crossovers)
**CS-1**	20°C	mean	0.10	0.90	20.05	0.00	0.00	0.00	41.00	43.95
		SD	0.45	0.64	0.60	0.00	0.00	0.00	0.65	1.50
		SE	0.10	0.14	0.14	0.00	0.00	0.00	0.15	0.34
		min	0	0	19	0	0	0	40	41
		max	2	2	21	0	0	0	42	47
**CS-2**	20°C	mean	0.08	0.96	20.00	0.00	0.00	0.00	40.96	43.92
		SD	0.40	0.93	1.00	0.00	0.00	0.00	1.10	1.19
		SE	0.08	0.19	0.20	0.00	0.00	0.00	0.22	0.24
		min	0	0	18	0	0	0	38	42
		max	2	3	21	0	0	0	42	46
**CS-3**	20°C	mean	0.25	1.38	19.50	0.00	0.00	0.00	40.38	43.00
		SD	0.68	1.17	1.32	0.00	0.00	0.00	1.53	1.50
		SE	0.14	0.24	0.27	0.00	0.00	0.00	0.31	0.31
		min	0	0	16	0	0	0	36	39
		max	2	4	21	0	0	0	42	45
**CS-4**	20°C	mean	0.26	1.48	19.39	0.00	0.00	0.00	40.30	43.91
		SD	0.69	0.95	1.08	0.00	0.00	0.00	1.33	1.93
		SE	0.14	0.20	0.22	0.00	0.00	0.00	0.28	0.40
		min	0	0	17	0	0	0	37	38
		max	2	3	21	0	0	0	42	46
**CS-5**	20°C	mean	0.18	1.18	19.73	0.00	0.00	0.00	40.64	43.45
		SD	0.59	0.85	0.94	0.00	0.00	0.00	1.09	1.60
		SE	0.13	0.18	0.20	0.00	0.00	0.00	0.23	0.34
		min	0	0	18	0	0	0	38	41
		max	2	3	21	0	0	0	42	46
ttmei1-1	20°C	mean	4.04	7.13	11.78	0.04	0.00	0.00	30.78	33.35
		SD	2.84	1.91	1.91	0.21	0.00	0.00	2.70	3.02
		SE	0.59	0.40	0.40	0.04	0.00	0.00	0.56	0.63
		min	0	4	8	0	0	0	25	26
		max	10	12	15	1	0	0	35	38
ttmei1-2	20°C	mean	1.90	5.35	14.00	0.30	0.00	0.10	34.35	35.05

		SD	1.68	1.76	1.97	0.57	0.00	0.31	1.95	2.31
		SE	0.38	0.39	0.44	0.13	0.00	0.07	0.44	0.52
		min	0	3	9	0	0	0	30	30
		max	6	9	17	2	0	1	38	40
ttmei1-3	20°C	mean	0.70	2.95	17.05	0.15	0.15	0.05	38.00	38.85
		SD	0.98	1.36	1.64	0.37	0.37	0.22	1.62	1.84
		SE	0.22	0.30	0.37	0.08	0.08	0.05	0.36	0.41
		min	0	1	15	0	0	0	36	36
		max	3	6	20	1	1	1	41	42
ttmei1-4	20°C	mean	1.83	3.78	16.30	0.00	0.00	0.00	36.39	39.61
		SD	1.70	1.76	2.05	0.00	0.00	0.00	2.61	3.19
		SE	0.35	0.37	0.43	0.00	0.00	0.00	0.54	0.66
		min	0	1	12	0	0	0	30	33
		max	6	7	20	0	0	0	41	45
ttmei1-5	20°C	mean	1.75	2.70	16.05	0.65	0.20	0.00	36.75	39.35
		SD	1.68	1.78	2.50	0.59	0.41	0.00	2.95	3.31
		SE	0.38	0.40	0.56	0.13	0.09	0.00	0.66	0.74
		min	0	0	12	0	0	0	29	34
		max	8	5	20	2	1	0	41	47
**CS-6**	13°C	mean	0.00	1.05	19.95	0.00	0.00	0.00	40.95	43.27
		SD	0.00	1.40	1.40	0.00	0.00	0.00	1.40	2.07
		SE	0.00	0.30	0.30	0.00	0.00	0.00	0.30	0.44
		min	0	0	16	0	0	0	37	38
		max	0	5	21	0	0	0	42	47
**CS-7**	13°C	mean	0.58	1.00	19.71	0.00	0.00	0.00	40.46	42.88
		SD	1.10	0.93	1.04	0.00	0.00	0.00	1.32	2.07
		SE	0.22	0.19	0.21	0.00	0.00	0.00	0.27	0.42
		min	0	0	18	0	0	0	37	39
		max	4	3	21	0	0	0	42	46
**CS-8**	13°C	mean	0.25	1.79	19.08	0.00	0.00	0.00	39.96	43.13
		SD	0.68	1.72	1.82	0.00	0.00	0.00	1.97	1.98
		SE	0.14	0.35	0.37	0.00	0.00	0.00	0.40	0.41
		min	0	0	13	0	0	0	33	37

		max	2	7	21	0	0	0	42	45
**CS-9**	13°C	mean	0.08	1.00	19.96	0.00	0.00	0.00	40.92	44.96
		SD	0.39	1.17	1.18	0.00	0.00	0.00	1.23	1.64
		SE	0.08	0.23	0.23	0.00	0.00	0.00	0.24	0.32
		min	0	0	17	0	0	0	38	42
		max	2	4	21	0	0	0	42	48
**CS-10**	13°C	mean	0.07	0.97	20.00	0.00	0.00	0.00	40.97	43.86
		SD	0.37	0.94	0.96	0.00	0.00	0.00	1.02	1.60
		SE	0.07	0.18	0.18	0.00	0.00	0.00	0.19	0.30
		min	0	0	18	0	0	0	39	41
		max	2	3	21	0	0	0	42	46
ttmei1-6	13°C	mean	32.87	4.30	0.26	0.00	0.00	0.00	4.83	5.00
		SD	3.45	1.61	0.45	0.00	0.00	0.00	1.95	2.13
		SE	0.72	0.34	0.09	0.00	0.00	0.00	0.41	0.44
		min	24	2	0	0	0	0	2	2
		max	38	9	1	0	0	0	9	10
ttmei1-7	13°C	mean	40.96	0.52	0.00	0.00	0.00	0.00	0.52	0.52
		SD	1.46	0.73	0.00	0.00	0.00	0.00	0.73	0.73
		SE	0.30	0.15	0.00	0.00	0.00	0.00	0.15	0.15
		min	38	0	0	0	0	0	0	0
		max	42	2	0	0	0	0	2	2
ttmei1-8	13°C	mean	40.79	0.61	0.00	0.00	0.00	0.00	0.61	0.68
		SD	1.26	0.63	0.00	0.00	0.00	0.00	0.63	0.72
		SE	0.24	0.12	0.00	0.00	0.00	0.00	0.12	0.14
		min	38	0	0	0	0	0	0	0
		max	42	2	0	0	0	0	2	2
ttmei1-9	13°C	mean	41.33	0.33	0.00	0.00	0.00	0.00	0.33	0.38
		SD	1.13	0.56	0.00	0.00	0.00	0.00	0.56	0.65
		SE	0.23	0.12	0.00	0.00	0.00	0.00	0.12	0.13
		min	38	0	0	0	0	0	0	0
		max	42	2	0	0	0	0	2	2
ttmei1-10	13°C	mean	41.48	0.26	0.00	0.00	0.00	0.00	0.26	0.26
		SD	1.08	0.54	0.00	0.00	0.00	0.00	0.54	0.54

		SE	0.23	0.11	0.00	0.00	0.00	0.00	0.11	0.11
		min	38	0	0	0	0	0	0	0
		max	42	2	0	0	0	0	2	2
**CS-11**	30°C	mean	0.57	2.48	18.24	0.00	0.00	0.00	38.95	42.00
		SD	1.12	1.25	1.34	0.00	0.00	0.00	1.63	2.12
		SE	0.24	0.27	0.29	0.00	0.00	0.00	0.35	0.45
		min	0	0	16	0	0	0	35	37
		max	4	5	21	0	0	0	42	46
**CS-12**	30°C	mean	0.29	2.38	18.48	0.00	0.00	0.00	39.33	41.57
		SD	0.72	1.16	1.17	0.00	0.00	0.00	1.28	1.75
		SE	0.16	0.25	0.25	0.00	0.00	0.00	0.28	0.38
		min	0	0	16	0	0	0	37	39
		max	2	5	21	0	0	0	42	45
**CS-13**	30°C	mean	0.20	1.45	19.45	0.00	0.00	0.00	40.35	42.90
		SD	0.62	1.05	1.15	0.00	0.00	0.00	1.31	1.12
		SE	0.13	0.23	0.25	0.00	0.00	0.00	0.29	0.24
		min	0	0	17	0	0	0	37	40
		max	2	3	21	0	0	0	42	45
**CS-14**	30°C	mean	0.00	1.79	19.21	0.00	0.00	0.00	40.21	43.04
		SD	0.00	1.22	1.22	0.00	0.00	0.00	1.22	1.27
		SE	0.00	0.25	0.25	0.00	0.00	0.00	0.25	0.26
		min	0	0	17	0	0	0	38	40
		max	0	4	21	0	0	0	42	45
**CS-15**	30°C	mean	0.00	1.88	19.12	0.00	0.00	0.00	40.12	42.68
		SD	0.00	1.36	1.36	0.00	0.00	0.00	1.36	1.84
		SE	0.00	0.27	0.27	0.00	0.00	0.00	0.27	0.36
		min	0	0	17	0	0	0	38	39
		max	0	4	21	0	0	0	42	46
ttmei1-11	30°C	mean	3.43	5.05	14.24	0.00	0.00	0.00	33.52	36.10
		SD	2.11	1.96	1.97	0.00	0.00	0.00	2.48	3.00
		SE	0.46	0.43	0.43	0.00	0.00	0.00	0.54	0.65
		min	2	2	11	0	0	0	28	29
		max	10	9	17	0	0	0	37	40

ttmei1-12	30°C	mean	4.23	6.14	12.09	0.32	0.09	0.00	31.32	34.32
		SD	2.37	1.64	1.23	0.48	0.29	0.00	1.91	2.28
		SE	0.51	0.35	0.26	0.10	0.06	0.00	0.41	0.49
		min	1	4	10	0	0	0	28	30
		max	10	9	14	1	1	0	35	39
ttmei1-13	30°C	mean	25.60	5.40	2.40	0.30	0.05	0.00	10.65	12.50
		SD	6.56	1.85	2.19	0.57	0.22	0.00	5.13	5.91
		SE	1.47	0.41	0.49	0.13	0.05	0.00	1.15	1.32
		min	15	1	0	0	0	0	1	1
		max	40	9	8	2	1	0	20	23
ttmei1-14	30°C	mean	10.54	8.67	6.46	0.29	0.08	0.00	22.42	25.54
		SD	4.75	2.87	3.05	0.46	0.28	0.00	4.71	5.45
		SE	0.97	0.59	0.62	0.09	0.06	0.00	0.96	1.11
		min	4	4	1	0	0	0	13	16
		max	23	14	12	1	1	0	30	34
ttmei1-15	30°C	mean	17.73	8.00	3.45	0.45	0.00	0.00	15.82	17.95
		SD	3.73	2.35	1.10	0.67	0.00	0.00	2.34	3.12
		SE	0.80	0.50	0.23	0.14	0.00	0.00	0.50	0.67
		min	10	4	1	0	0	0	11	12
		max	23	13	5	2	0	0	20	23
ttmei1-16	30°C	mean	5.43	7.48	10.24	0.38	0.00	0.00	28.71	30.33
		SD	2.71	2.02	2.23	0.74	0.00	0.00	3.07	2.83
		SE	0.55	0.41	0.46	0.15	0.00	0.00	0.63	0.58
		min	0	3	8	0	0	0	25	26
		max	10	10	16	2	0	0	37	36
ttmei1-17	30°C	mean	3.86	5.73	13.27	0.05	0.00	0.00	32.05	34.27
		SD	1.96	2.07	2.14	0.21	0.00	0.00	2.54	2.57
		SE	0.42	0.44	0.46	0.05	0.00	0.00	0.54	0.55
		min	0	4	8	0	0	0	27	29
		max	6	11	16	1	0	0	37	39
ttmei1-18	30°C	mean	3.77	7.05	10.50	0.50	0.23	0.09	30.32	32.68
		SD	2.35	2.24	1.87	0.67	0.43	0.29	1.99	2.38
		SE	0.50	0.48	0.40	0.14	0.09	0.06	0.42	0.51

		min	1	3	8	0	0	0	25	27
		max	10	11	14	2	1	1	34	36
ttmei1-19	30°C	mean	3.52	5.83	12.57	0.57	0.00	0.00	32.09	34.48
		SD	1.95	2.31	2.15	0.66	0.00	0.00	2.68	2.61
		SE	0.41	0.48	0.45	0.14	0.00	0.00	0.56	0.54
		min	1	2	8	0	0	0	27	30
		max	7	11	16	2	0	0	37	40
ttmei1-20	30°C	mean	2.72	6.00	13.60	0.00	0.00	0.00	33.20	35.44
		SD	1.99	2.25	2.35	0.00	0.00	0.00	2.78	2.71
		SE	0.40	0.45	0.47	0.00	0.00	0.00	0.56	0.54
		min	0	2	9	0	0	0	26	28
		max	8	10	18	0	0	0	38	40
ttmei1-21	30°C	mean	5.50	7.03	10.97	0.17	0.00	0.00	29.30	30.70
		SD	2.73	2.01	2.28	0.46	0.00	0.00	3.31	3.52
		SE	0.50	0.37	0.42	0.08	0.00	0.00	0.60	0.64
		min	2	2	6	0	0	0	20	22
		max	14	10	15	2	0	0	35	37
